# Neurovascular coupling in the basolateral amygdala modulates negative emotions

**DOI:** 10.1038/s41422-026-01256-2

**Published:** 2026-05-27

**Authors:** Jiayu Ruan, Xinghua Quan, Huiqi Xie, Yiyi Zhang, Dongdong Zhang, Wentao Wang, Bingrui Zhao, Danping Lu, Yuxiao Niu, Jie-Min Jia

**Affiliations:** 1 College of Life Sciences, Zhejiang University, Hangzhou, Zhejiang China; 2 Westlake Laboratory of Life Sciences and Biomedicine, Hangzhou, Zhejiang China; 3 Laboratory of Neurovascular Biology, School of Life Sciences, Westlake University, Hangzhou, Zhejiang China; 4 Laboratory of Neurovascular Biology, Institute of Basic Medical Sciences, Westlake Institute for Advanced Study, Hangzhou, Zhejiang China; 5 Laboratory of Computational and Functional Genomics, School of Life Sciences, Westlake University, Hangzhou, Zhejiang China; 6 State Key Laboratory of Gene Expression, School of Life Sciences, Westlake University, Hangzhou, Zhejiang China

**Keywords:** Cell biology, Molecular biology

## Abstract

Emotion induces changes in regional cerebral blood flow, a manifestation of neurovascular coupling (NVC). However, whether NVC provides feedback to actively modulate emotion remains unexplored. Here, we demonstrate that NVC actively and bidirectionally modulates stress-induced negative emotions. We established bidirectional manipulations of NVC in freely moving mice by employing integrated pharmacological, genetic, and arteriolar optogenetic approaches. Our results showed that both systemic and region-specific NVC deficiencies in the basolateral amygdala (BLA) heightened emotional responses when mice transitioned from a safe, familiar environment to anxiogenic environments and that local restoration of NVC in the BLA normalized these responses. Mechanistically, NVC dysfunction impaired the capacity of BLA neuronal scaling during state transitions, manifesting as a characteristic biphasic pattern of c-Fos topology. NVC-deficient animals aberrantly adopted high-stress configurations under mild stress but regressed to low-stress templates during high-demand survival threats, thereby compromising defensive sustainability. Notably, the genetic NVC-enhancement model counteracted NVC impairments caused by chronic stress, thereby alleviating stress-driven emotional distress. These findings establish NVC in the BLA as an allostatic program that fine-tunes neural circuit activity during emotional responses, with implications for understanding and treating emotional disorders.

## Introduction

Emotion is a complex, integrative response to changes in internal and external environments.^1^ Emotional flexibility — the capacity to dynamically regulate and adapt these responses — is fundamental for mental health and survival.^2^ Conversely, deficits in this adaptive capacity result in emotional rigidity, a hallmark of dysregulation that is strongly associated with psychiatric disorders such as depression and post-traumatic stress disorder (PTSD).^3,4^ Therefore, delineating the molecular and cellular mechanisms that govern the balance between emotional flexibility and rigidity is critical for elucidating the biological basis of resilience.^5,6^

Neurovascular coupling (NVC), the core physiological foundation of blood oxygen level-dependent functional MRI (BOLD fMRI) signals, has long been categorized as a passive neural proxy.^7–10^ Clinical observations in the basolateral amygdala (BLA) — a pivotal hub for processing negative affect — reveal pervasive neurovascular dysregulation across diverse psychiatric landscapes.^11^ Specifically, exaggerated BOLD responses occur in PTSD, bipolar disorder, high body mass index (BMI), and cohorts exposed to early-life stress.^8,12–15^ In contrast, autosomal recessive Urbach-Wiethe disease, stemming from mutations in the *ECM1* gene, is marked by a lack of hemodynamic responsiveness; such patients exhibit BLA vascular perturbations leading to localized calcification, alongside a selective deficit in fear perception when confronted with threat.^16,17^

Consistent with this clinical phenotype, NVC impairments are recapitulated in animal models, where acute, chronic, and neuroendocrine stressors significantly impair NVC fidelity within stress-sensitive circuits.^18–20^ However, a fundamental question of causality remains: do these deficits merely reflect secondary consequences of neuronal dysfunction, or do they actively drive emotional pathology?

Emerging evidence indicates that vascular-derived cues actively modulate diverse neural processes. For instance, nitric oxide generated during NVC promotes adult hippocampal neurogenesis,^21^ while vascular-derived nitric oxide depolarizes the membrane potential of the optic nerve.^22^ In the hypothalamus, salt-loading-induced vasoconstriction during NVC exerts positive feedback that enhances local vasopressin neuronal activity.^23^ Additionally, whole-cell patch recordings in brain slices demonstrate that altered parenchymal arteriolar tone modulates nearby neuronal firing, decreasing pyramidal neuron activity and increasing interneuron activity, with effects depending on a mechanosensitive cation channel.^24^ These observations imply that NVC possesses an intrinsic capacity, likely via chemical and/or mechanical cues, to actively regulate neural circuits, extending beyond its traditional role in metabolic support.^25^

However, its potential as a real-time gatekeeper of emotional circuitry remains unexplored. To address this gap, we investigated NVC within the BLA as a model system. We hypothesized that BLA NVC functions as a critical regulatory checkpoint that calibrates the intensity of negative emotions, thereby maintaining emotional flexibility.^26^

In this study, we employed a multidimensional emotion assessment framework combined with cell type-specific genetic manipulations and arteriolar optogenetics to demonstrate that NVC in the BLA actively and bidirectionally modulates emotional reactivity. In contrast to its lack of contribution in the somatosensory barrel cortex (S1BF), we found that neurovascular decoupling in the BLA precipitated emotional rigidity — a maladaptive state defined by a collapse in the dynamic range of emotional responses — manifesting as a distinctive biphasic dysregulation in which physiological and behavioral outputs were pathologically exaggerated under mild stress yet paradoxically blunted under intense stress. Mechanistically, we showed that this behavioral rigidity was underpinned by corresponding biphasic shifts in BLA neural activity across graded stressors. Notably, the enhanced NVC model conferred emotional resilience against chronic stress by normalizing BLA neural circuit activity. These findings establish NVC as an intrinsic regulatory gain controller in emotional processing and suggest that targeting circuit-specific neurovascular communication offers a novel therapeutic strategy for psychiatric dysregulation.

## Results

### Validation of a quantitative behavioral framework linking graded BLA activation to escalating spatial stress levels

**Fig. 1:**
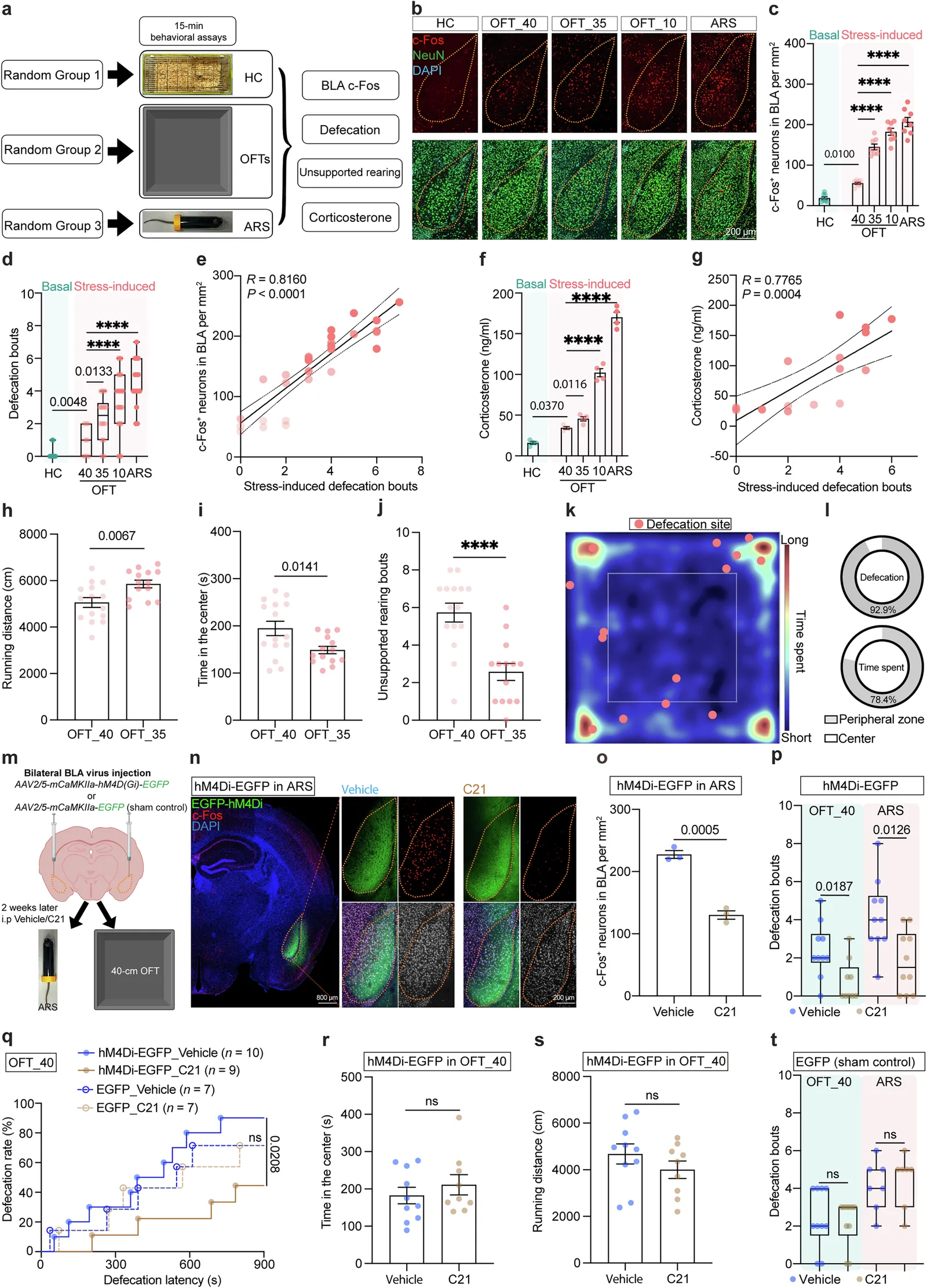
Validation of defecation bouts as a robust quantitative index of negative emotional states. **a**–**c** Experimental design for HC, OFT, and ARS assays. Representative images (**b**) and quantification (**c**) of BLA neuronal activation across the different stress paradigms shown in **a**. One-way ANOVA followed by Tukey’s multiple comparisons test (*P* < 0.0001). **d** Quantification of defecation bouts under the paradigms shown in **a**. Kruskal-Wallis test followed by Dunn’s multiple comparisons test (*P* < 0.0001). **e** Linear correlation between BLA c-Fos-positive neuron counts and defecation bouts. Data were pooled from **c**, **d**. Dotted lines represent SEM. **f**, **g** Serum corticosterone concentrations (**f**) under the paradigms shown in **a** and their linear correlation with defecation bouts (**g**). Data in **g** were pooled from **d**, **f**. One-way ANOVA followed by Tukey’s multiple comparisons test (*P* < 0.0001). Dotted lines represent SEM in **g**. **h**–**j** Total running distance (**h**), time spent in the center zone (**i**), and unsupported rearing bouts (**j**) in the 40-cm and 35-cm OFT. Student’s *t-*test (**h,** **i**) and Mann-Whitney tes*t* (**j**). **k** Representative heatmap of zone preference for a single mouse and the spatial distribution of defecation locations from 15 mice in the 40-cm OFT. Each dot indicates one defecation location. **l** Statistical quantification of the spatial distribution data in **k**. **m** Experimental schematic of chemogenetic inhibition of BLA neurons using AAV2/5-mCaMKIIa-hM4D(Gi)-EGFP or AAV2/5-mCaMKIIa-EGFP (sham control). **n**, **o** Representative images (**n**) and quantification (**o**) of BLA neuronal activation under chemogenetic inhibition during ARS. Student’s *t-*test. **p** Quantification of defeca*t*ion bouts in 40-cm OFT and ARS following BLA chemogenetic inhibition. Mann-Whitney test. **q** Defecation rate and defecation latency in the 40-cm OFT. Sample sizes (*n*) are indicated. Gehan-Breslow-Wilcoxon test. **r**–**s** Time spent in the center zone (**r**) and total running distance (**s**) following chemogenetic inhibition in the 40-cm OFT. Student’s *t-*test. **t** Defecation bouts in the 40-cm OFT and ARS following BLA injection of the control virus (EGFP). Mann-Whitney test. Except for **k**, each dot represents one mouse. ****P < 0.0001. Data are presented as mean ± SEM.

To dissect the neurovascular mechanisms actively modulating emotion, we first established a robust, quantifiable behavioral paradigm that scales linearly with stress intensity. We exposed male age-matched wild-type (WT) mice to contexts with graded anxiogenic potential for 15 min: the home cage (HC; minimal stress); the 40-cm, 35-cm, and 10-cm open field test (OFT; mild-to-moderate stress); and acute restraint stress (ARS; intense stress) (Fig. 1a).^27–31^ We verified BLA neuronal activation 90 min post-assay by assessing c-Fos expression (Fig. 1b). Immunofluorescence (IF) mapping revealed that c-Fos intensity increased in a stress-dependent manner (Fig. 1c). Crucially, this graded BLA activation was mirrored by a stepwise increase in defecation bouts (Fig. 1d), showing a robust linear correlation with BLA c-Fos intensity (*R* = 0.8160, *P* < 0.0001; Fig. 1e). Furthermore, we quantified serum corticosterone levels, the primary stress-responsive glucocorticoid in rodents, in a parallel cohort of mice at 5 min post-assay (Fig. 1a). Serum corticosterone concentrations exhibited a stress-dependent trend mirroring that of BLA neural c-Fos expression (Fig. 1f). Likewise, defecation bouts also showed a strong positive correlation with serum corticosterone concentrations (*R* = 0.7765, *P* = 0.0004; Fig. 1g). Collectively, these results establish defecation as a reliable, robust, and noninvasive marker that accurately tracks systemic and spatially graded stress levels in freely moving live mice.^31–33^

Reducing the arena size from 40 cm to 35 cm markedly increased defecation bouts (Fig. 1d). Although the shift in linear dimensions from 40 cm to 35 cm appears modest, this modification represents a ~33% reduction in total cubic volume, substantially intensifying the degree of spatial confinement.^34^ This compressed environment provoked a more potent stress state than the 40-cm OFT, characterized by a multi-dimensional escalation in BLA c-Fos density (Fig. 1c), serum corticosterone levels (Fig. 1f), and locomotor activity (Fig. 1h), coupled with reductions in classic readouts of exploration, specifically center time (Fig. 1i) and unsupported rearing bouts (Fig. 1j). Spatial mapping further revealed that more than 90% of defecation behavior in the 40-cm OFT occurred in the peripheral zone, consistent with thigmotaxis behavior (Fig. 1k, l).^27,35^ Together, the converging behavioral, neural, and hormonal readouts confirm that the 35-cm OFT elicits a stronger stress response than the standard 40-cm OFT.^36^

To determine whether BLA neuronal activity is necessary for this response, we chemogenetically silenced BLA excitatory neurons using the inhibitory Designer Receptors Exclusively Activated by Designer Drugs (DREADD) hM4Di (Fig. 1m).^37^ In mice expressing hM4Di in BLA excitatory neurons and treated with the agonist compound 21 (C21), stress-induced BLA c-Fos expression under ARS was effectively blunted (Fig. 1n, o). Intriguingly, this treatment also suppressed defecation bouts in both the 40-cm OFT and ARS contexts compared with vehicle-treated control mice (Fig. 1p). Furthermore, BLA silencing significantly reduced the defecation rate (i.e., incidence of defecation) and prolonged defecation latency (time elapsed before production of the first fecal pellet) in the 40-cm OFT (Fig. 1q). Interestingly, neither center preference nor locomotor activity was altered in the 40-cm OFT following chemogenetic inhibition of the BLA (Fig. 1r, s). Control mice expressing enhanced green fluorescent protein (EGFP) alone (sham group) exhibited no alterations in defecation metrics (Fig. 1q, t).

Collectively, these data establish a validated, BLA-dependent quantitative framework — the “negative emotion indices” — encompassing BLA neural activation, multi-dimensional defecation metrics (bouts, rate, and latency), unsupported rearing, and serum corticosterone concentration to dynamically assess emotional states.

### A genetic model of cerebral SMC-specific GluN2D deficiency exhibits BLA neurovascular decoupling

**Fig. 2:**
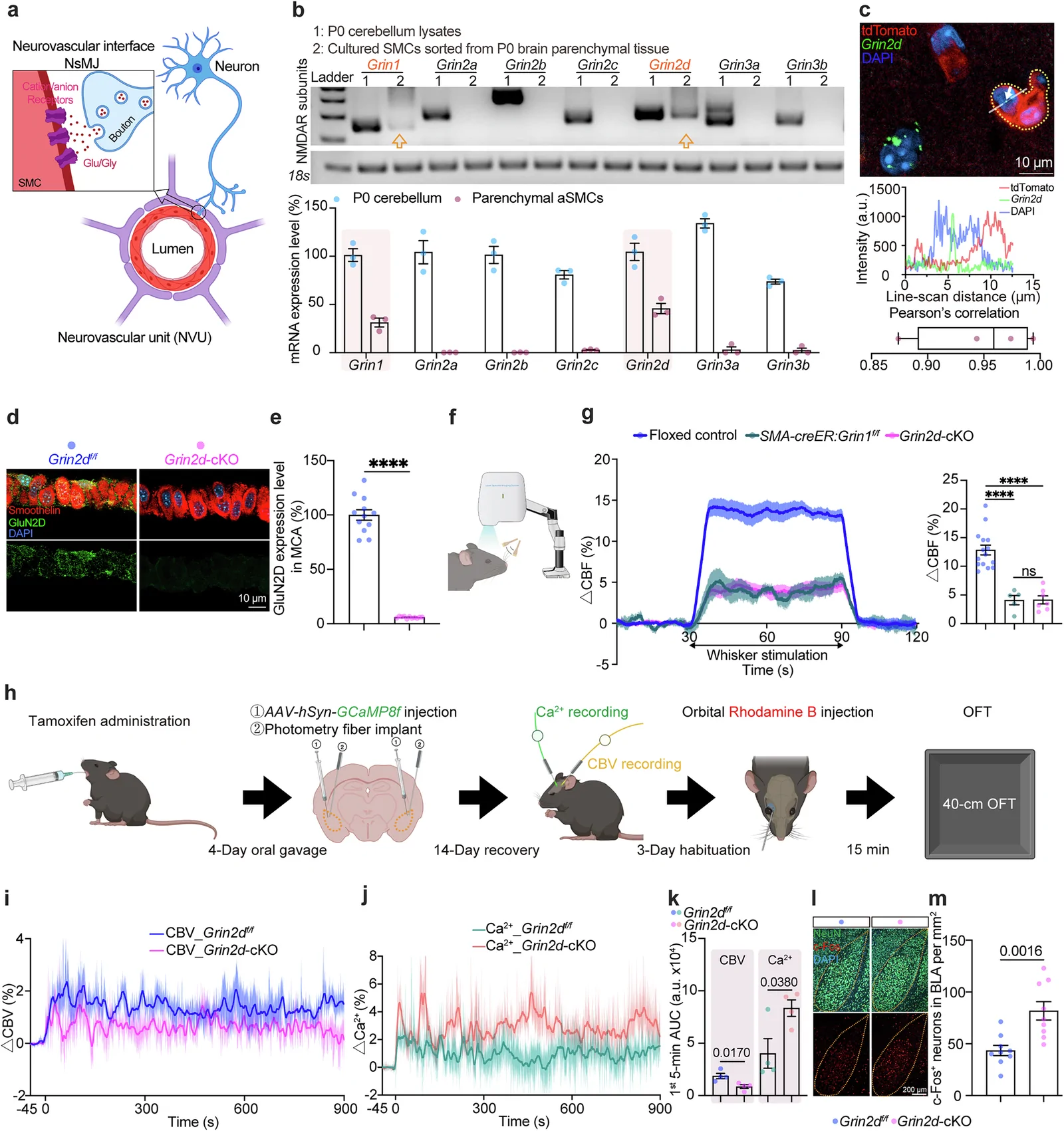
GluN2D in arteriolar SMCs is essential for NVC in S1BF and BLA. **a** Schematic representation of the neuro–smooth muscle junction (NsMJ) within the NVU. **b** Electrophoretogram (top) and quantification (bottom) of mRNA levels of NMDA receptor subunits detected by RT-PCR in cultured SMCs sorted from P0 brain parenchymal tissue. P0 cerebellum lysates were used as a benchmark. Arrows indicate band locations. **c** Validation of *Grin2d* expression by RNAscope. Top: representative image from the cortex of a brain slice (yellow dotted line indicates the arteriolar SMC boundary). Middle: representative fluorescence intensity line scan of the region indicated by white arrows. Bottom: Pearson’s correlation coefficient analysis. Each dot represents one line-scan region from one mouse. **d**, **e** Representative images (**d**) and quantification (**e**) of GluN2D IF staining in the MCA. Each dot represents one analysis region of the MCA from a single mouse. Student’s *t-*test. **f**, **g** Experimental schematic (**f**) and whisker-evoked functional hyperemia in the S1BF (**g**). In **g**, the left panel shows time-series traces of functional hyperemia, and the shaded areas represent SEM across mice; the right panel shows quantification of the functional hyperemia plateau phase. One-way ANOVA followed by Tukey’s multiple comparisons test (*P* < 0.0001). Floxed control mice included *Grin2d*^*f/f*^ mice (*n* = 8) and *Grin1*^*f/f*^ mice (*n* = 7). **h** Schematic for concurrent recording of CBV (via Rhodamine B) and neuronal Ca^2+^ (via AAV2/9-hSyn-GCaMP8f) signals in the BLA. **i**, **j** Time-series recordings of BLA CBV (**i**) and Ca^2+^ signals (**j**) during the 15-min (900-s) 40-cm OFT session. Shaded areas represent SEM across mice. **k** Area under the curve (AUC) quantification of the first 5 min of CBV and Ca^2+^ responses from the data in **i**, **j**. Student’s *t-*test. **l**, **m** Representative images (**l**) and quantification (**m**) of BLA neuronal activation following the 40-cm OFT. Student’s *t-*test. For all panels except **c**, **e**, each dot represents one mouse. ****P < 0.0001. Data are presented as the mean ± SEM. See also Supplementary information, Figs. S1–S8.

Equipped with this multi-dimensional assessment framework, we next sought to identify the specific molecular substrates within the BLA NVU that govern these emotional responses. We previously identified that the core N-methyl-D-aspartate (NMDA) receptor subunit GluN1 (encoded by *Grin1*) in smooth muscle cells (SMCs) is essential for NVC at the neuro-smooth muscle junction (NsMJ; Fig. 2a),^38^ but the other regulatory NMDA subunits in SMCs remained to be characterized. To address this, we screened mRNA expression of all NMDA receptor subunits in cultured SMCs sorted from postnatal day 0 (P0) brain parenchymal tissue and identified *Grin2d* (encoding GluN2D) as the sole regulatory subunit that was detectably expressed (Fig. 2b). High-resolution RNAscope further confirmed that *Grin2d* colocalized specifically with SMC markers in cerebral arterioles with high colocalization coefficients (visualized via *SMA-creER;Ai14* reporter mice; Fig. 2c). To rigorously validate the specificity of our RNAscope probes, we leveraged the well-documented developmental divergence of NMDA subunits in the cerebellum as an internal biological control. We exploited the distinct trajectories in which *Grin1* expression increases from P0 to P60, whereas *Grin2d* expression follows a reciprocal decline.^39,40^ Our probes accurately recapitulated these age-dependent patterns, thereby confirming their high target specificity (Supplementary information, Fig. S1a, b). This biological validation was further extended to the protein level; our anti-GluN2D antibody successfully distinguished differential GluN2D protein abundance between the P0 and P60 stages in cerebellar tissue, mirroring the transcriptomic data (Supplementary information, Fig. S1c).

Using this validated antibody, IF analysis of isolated middle cerebral arteries (MCAs) (Fig. 2d, e) and cultured SMCs sorted from P0 brain parenchymal tissue (Supplementary information, Fig. S1d) confirmed the specific ablation of GluN2D in cerebral SMCs in *SMA-creER; Grin2d*^*f/f*^ (*Grin2d*-cKO) mice. Furthermore, western blot analysis of whole-cortical lysates revealed that *Grin2d*-cKO did not affect GluN2D expression in other non-SMC populations (Supplementary information, Fig. S1e), confirming the cell-type specificity of our genetic model. To assess the global impact of this genetic depletion, we examined GluN2D expression in SMCs of peripheral organs, including the heart, liver, spleen, lung, kidney, intestine, and bladder (Supplementary information, Fig. S2a). Notably, GluN2D expression appeared to be restricted to cerebral SMCs and absent from SMCs of these peripheral tissues, suggesting that our *Grin2d*-cKO model exerts a brain-restricted impact (Supplementary information, Fig. S2b, c). Furthermore, we systematically verified the physiological status of *Grin2d*-cKO mice; no differences were observed in systemic metabolic homeostasis (Supplementary information, Fig. S3a–g), blood pressure (Supplementary information, Fig. S3h), gastrointestinal function (Supplementary information, Fig. S3i–n), or blood–brain barrier (BBB) integrity^41,42^ (Supplementary information, Fig. S3o–p) compared with littermate controls. Finally, we assessed key contractile proteins, finding that α-SMA and smoothelin expression remained unaltered in *Grin2d*-cKO mice (Supplementary information, Fig. S4). These data demonstrate that basic metabolic functions, the gastrointestinal system, and the contractile structural integrity of SMCs are preserved in *Grin2d*-cKO mice.

To assess the functional impact of SMC-specific *Grin2d* deletion and facilitate a direct, one-to-one comparison with our previously reported *Grin1*-cKO model, we employed the same classic paradigm: measuring whisker stimulation-evoked hemodynamic responses in the S1BF (Fig. 2f).^38^ Consistently, *Grin2d*-cKO mice exhibited a ~66% attenuation in functional hyperemia upon whisker stimulation (Fig. 2g), confirming that GluN2D is functionally comparable to GluN1 in regulating cerebral NVC. Control experiments confirmed that tamoxifen administration alone in *Grin2d*^*f/f*^ mice did not alter NVC responses (Supplementary information, Fig. S5).

Having established the NVC deficit in the cortex, we sought to determine whether this NVC dysfunction extends to the BLA. To this end, we developed a de novo system based on previous strategies^43,44^ to simultaneously record neuronal activity and blood supply within the BLA of freely moving mice, thereby enabling direct assessment of NVC. First, we mapped the BLA arteriolar topology by superimposing 3D-reconstructed arterioles in the tissue-cleared brain on a schematic cerebral vasculature. This analysis showed that the corticoamygdaloid artery (coamg) penetrates the BLA, making it suitable for photometric monitoring (Supplementary information, Fig. S6, Video S1). Second, we validated the fidelity of our cerebral blood volume (CBV) recording method: fiber photometry-based recording of blood plasma fluorescence intensity in the BLA showed a robust linear correlation with orbital Rhodamine B injection concentration (Supplementary information, Fig. S7a, b; *R* = 0.8946, *P* < 0.0001). Signal stability was achieved 15 min post-injection (Supplementary information, Fig. S7c), and this time point was designated as the initiation time for the subsequent behavioral assay.

Using this optimized paradigm, after tamoxifen administration, we injected AAV2/9-hSyn-GCaMP8f into the BLA and implanted optic fibers at the injection site 2 weeks before the recordings. After three days of habituation (one trial per day for a continuous 3-day period), we monitored NVC during the 40-cm OFT (Fig. 2h–k). Consistent with our earlier c-Fos data (Fig. 1c), the 40-cm OFT elicited robust BLA Ca^2+^ transients that were temporally matched by CBV elevations, indicating tight local NVC in littermate control mice (Fig. 2i–k). In contrast, *Grin2d*-cKO mice exhibited a dissociated neurovascular phenotype, in which augmented BLA neuronal Ca^2+^ signals were paradoxically paired with a blunted hemodynamic response (Fig. 2i–k). This inverse pattern — hyperactive neurons coupled with hypoperfused blood supply — was further corroborated by increased c-Fos expression in the BLA 90 min post-assay (Fig. 2l, m). Furthermore, we confirmed that the observed attenuation in functional hyperemia was not attributable to differences in BLA basal perfusion, locomotor activity, or spatial preference during the 40-cm OFT (Supplementary information, Fig. S8)

Collectively, these findings demonstrate robust NVC within the BLA during the 40-cm OFT and validate that SMC-specific *Grin2d*-cKO mice exhibit an NVC deficit in this region.

### BLA-targeted pharmacological restoration of NVC reversed emotional hyperreactivity in *Grin2d*-cKO mice under mild stress

**Fig. 3:**
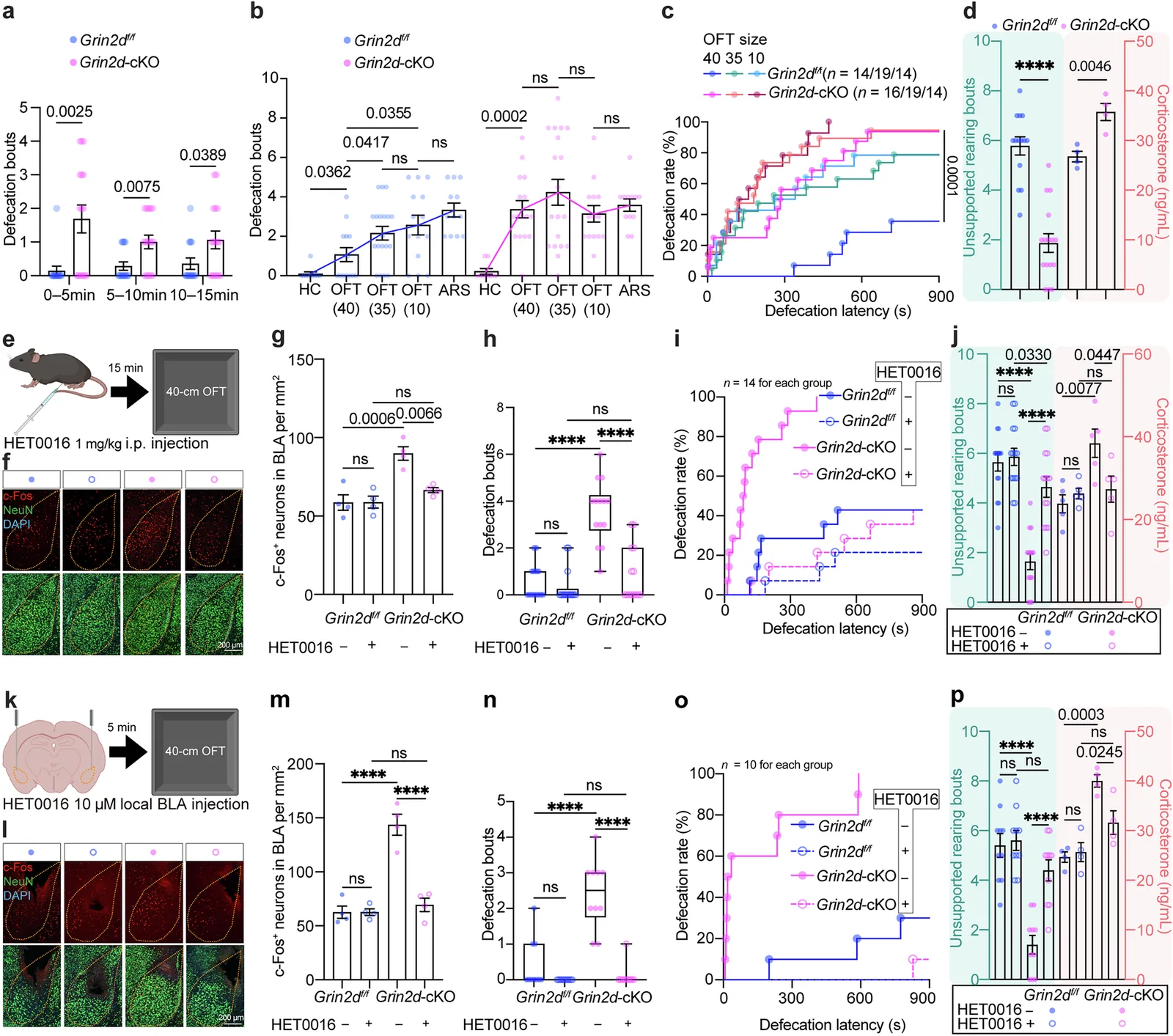
Systemic SMC-specific GluN2D deficiency-driven negative emotionality is pharmacologically rescued by local BLA intervention. **a** Defecation bouts in the 40-cm OFT shown in 5-min intervals. Mann-Whitney test. **b** Comparison of defecation bouts across HC, OFT, and ARS paradigms. Kruskal-Wallis test followed by Dunn’s multiple comparisons test (*P* = 0.0365). **c** Defecation rate and defecation latency in the OFT. Sample sizes (*n*) are indicated. Gehan-Breslow-Wilcoxon test. **d** Unsupported rearing bouts (left) and serum corticosterone concentrations (right) in the 40-cm OFT. Mann-Whitney test (left) and Student’s *t*-test (right). **e** Experimental schematic of systemic HET0016 administration. **f**, **g** Representative images (**f**) and quantification (**g**) of BLA neuronal activation following systemic HET0016 treatment in the 40-cm OFT. Two-way ANOVA followed by Tukey’s multiple comparisons test (*P* = 0.0104). **h**–**j** Quantification of defecation bouts (**h**), defecation rate and latency (**i**), and unsupported rearing bouts and serum corticosterone concentrations (**j**) in the 40-cm OFT following systemic HET0016 administration. Two-way ANOVA on rank-transformed data followed by Sidak’s multiple comparisons test (**h**, *P* < 0.0001; **j** left, *P* = 0.00003), Gehan-Breslow-Wilcoxon test (**i**), and two-way ANOVA followed by Tukey’s multiple comparisons test (**j** right, *P* = 0.0212). Sample sizes (*n*) are indicated in **i**. **k** Experimental schematic of local BLA microinjection of HET0016. **l**, **m** Representative images (**l**) and quantification (**m**) of BLA neuronal activation following local BLA HET0016 treatment in the 40-cm OFT. Two-way ANOVA followed by Tukey’s multiple comparisons test (*P* < 0.0001). **o**–**p** Quantification of defecation bouts (**n**), defecation rate and latency (**o**), and unsupported rearing bouts and serum corticosterone concentrations (**p**) in the 40-cm OFT following local BLA HET0016 administration. Two-way ANOVA on rank-transformed data followed by Sidak’s multiple comparisons test (**n**, *P* < 0.0001; **p** left, *P* = 0.0020), Gehan-Breslow-Wilcoxon test (**o**), and two-way ANOVA followed by Tukey’s multiple comparisons test (**p** right, *P* = 0.0190). Sample sizes (*n*) are indicated in **o**. Each dot represents one mouse. ****P < 0.0001. Data are presented as mean ± SEM. Detailed statistical data for **c**, **i**, **o** are shown in Supplementary information, Table S1, sub-tables 1–3. See also Supplementary information, Figs. S9–S13.

Building on the established role of the BLA as one of the central hubs for stress responses, we next utilized our negative emotion indices (Fig. 1) to evaluate whether the BLA neurovascular decoupling observed in *Grin2d*-cKO mice (Fig. 2) results in behavioral and physiological impairments. Notably, we observed that *Grin2d*-cKO mice exhibited a significant increase in defecation bouts during the first 5 min of the 40-cm OFT, a trend sustained throughout the session (Fig. 3a). This phenomenon was not observed in the home cage context (Supplementary information, Fig. S3c–e, n). Littermate control mice (*Grin2d*^*f/f*^) displayed a graded increase in defecation bouts in response to escalating spatial stress (Fig. 3b), consistent with the pattern observed in WT mice (Fig. 1d). In contrast, *Grin2d-*cKO mice failed to show this graded response; instead, they exhibited an abrupt increase to a maximal plateau level of defecation bouts, a response typically elicited by intense ARS stress (Fig. 3b). This hyperreactivity was further evidenced by an increased defecation rate, a reduced latency (Fig. 3c), diminished unsupported rearing, and elevated serum corticosterone levels following the 40-cm OFT (Fig. 3d). Importantly, basal stress levels in the home cage remained indistinguishable between genotypes (Supplementary information, Fig. S9a). Overall, *Grin2d*-cKO mice displayed a unified shift across multi-dimensional stress metrics, indicating a robust, exaggerated negative emotional response to mild stressors, such as those encountered in OFT.

Given that *Grin2d*-cKO mice showed NVC dysfunction beyond the BLA, we sought to determine the contribution of local BLA NVC to hyperreactivity by administering HET0016, a selective inhibitor of CYP450ω-hydroxylases that suppresses the synthesis of 20-HETE (a potent vasoconstrictor) and thereby facilitates vasodilation to compensate for the NVC deficit.^45,46^

To strictly validate the pharmacological efficacy of HET0016 before its site-specific delivery to the BLA, we first replicated the systemic administration protocol described in our previous study.^38^ Systemic HET0016 administration dose-dependently reduced ARS-induced defecation bouts (Supplementary Information, Fig. S10a).

This observed behavioral change provided a clear rationale for selecting 1 mg/kg as the optimal dose of HET0016 for subsequent investigations. At this dose, systemic HET0016 administration elicited a moderate elevation in the basal perfusion of *Grin2d*^*f/f*^ mice (Supplementary information, Fig. S11c), consistent with previous reports in both renal^47^ and cerebral^48^ vasculature. Furthermore, HET0016 treatment produced a robust and sustained enhancement in the amplitude of whisker-evoked functional hyperemia in the S1BF of *Grin2d*^*f/f*^ mice compared with saline-treated controls (Supplementary information, Fig. S10b, c).

Notably, HET0016 effectively rescued the hyperreactive phenotype in *Grin2d*-cKO mice following both systemic (Fig. 3e–j) and local BLA (Fig. 3k–p) administration in the 40-cm OFT. Both administration routes effectively normalized BLA neural activation and multi-dimensional defecation metrics — including bouts, rate, and latency — to littermate control levels (Fig. 3f–i and l–o). Importantly, this vascular intervention also significantly normalized unsupported rearing bouts and reduced serum corticosterone concentrations in Grin2d-cKO mice in the 40-cm OFT (Fig. 3j, p). In addition, systemic administration of HET0016 elevated basal perfusion (Supplementary information, Fig. S11c) and enhanced functional hyperemia in *Grin2d*-cKO mice (Supplementary information, Fig. S10b, c). In contrast, neither systemic nor local BLA HET0016 administration impacted serum corticosterone levels in the home cage (Supplementary information, Fig. S9b, c).

Collectively, these findings show that local pharmacological compensation in the BLA sufficiently normalized the hyperreactivity features in the *Grin2d*-cKO genetic model, despite NVC deficits in other brain regions, and thereby establish that intact NVC in the BLA is essential for maintaining appropriate emotional responses to mild stress.

We observed consistent behavioral phenotypes in the 40-cm OFT across various mechanistically distinct models of NVC dysfunction, each targeting different nodes of the NVU (Supplementary information, Figs. S12–S13). Specifically, either perturbation of upstream neural signaling (*Emx1-cre;Ptgs2*^*f/f*^) (Supplementary information, Figs. S12a–h and S13o–p)^49–51^ or disruption of the neurovascular interface — achieved via pan-mural cell-specific *Grin2d*-cKO (*Pdgfrβ-creER;Grin2d*^*f/f*^) or SMC-specific *Grin1-Grin2d* double-cKO (*SMA-creER;Grin1*^*f/f*^*;Grin2d*^*f/f*^) (Supplementary information, Figs. S12i–r and S13o, p) — consistently recapitulated the NVC deficits and behavioral phenotypes observed in the primary *Grin2d*-cKO mice in the 40-cm OFT (Figs. 2–3).

To disturb contractile protein machinery within SMCs, we targeted the *Acta2* gene (encoding α-SMA).^52,53^ This model (*Pdgfrβ-creER;Acta2*^*f/f*^ mice, hereafter referred to as *Acta2*-cKO) indistinguishably mirrored the hyperreactive phenotypes of the signaling-deficient models in the 40-cm OFT (Supplementary information, Figs. S12s–v, x–aii and S13o, p). Surprisingly, a high-fat diet (HFD) — known to diminish NVC in both animals and humans via multiple factors^14,54 ^— produced a phenotype identical to that of *Grin2d*-cKO mice in the 40-cm OFT (Supplementary information, Fig. S13a–d, g, h, j–p). These findings indicate that this emotional hyperreactivity was not restricted to the *Grin2d*-cKO model but represented a universal consequence of neurovascular dysfunction in the 40-cm OFT, reinforcing the universality of NVC-driven emotional dysregulation.

Although local HET0016 delivery to the BLA successfully restored emotional metrics in other models (Fig. 3k–p; Supplementary information, Fig. S13i–n) in the 40-cm OFT, it failed to produce similar effects in *Acta2*-cKO mice (Supplementary information, Fig. S12w–aii) in the same context. This indicates that once the downstream contractile apparatus is disrupted, upstream regulators are unable to exert their functions. Finally, we confirmed that these convergent effects did not involve BBB leakage (Supplementary information, Figs. S3o, p and S13e, f).^55^ Together, these findings establish that NVC integrity, per se, is indispensable for emotional homeostasis.

### Caldesmon acts as a downstream effector of GluN2D in NVC, modulating emotional reactivity

**Fig. 4:**
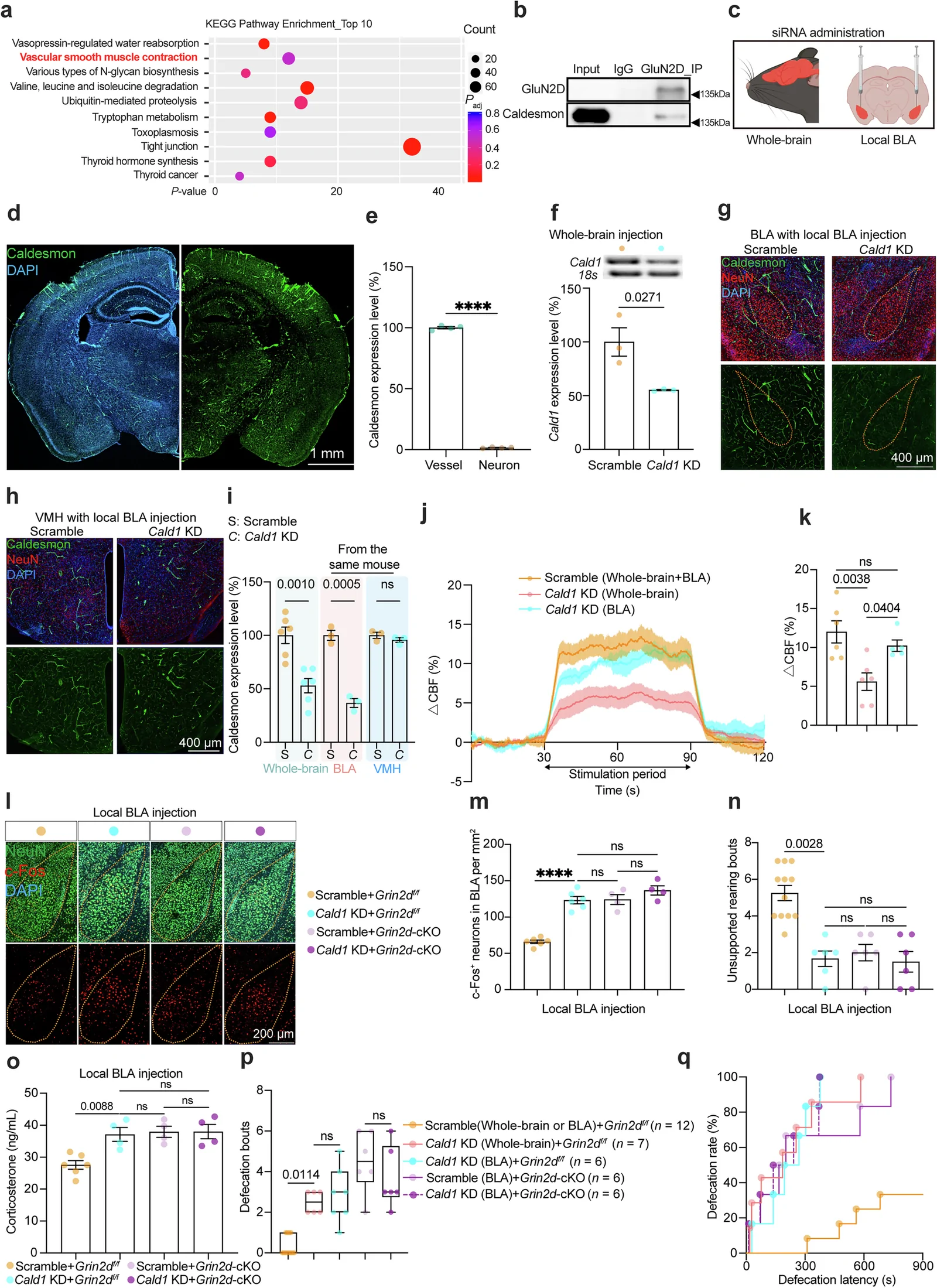
Caldesmon interacts with GluN2D in SMCs to regulate neurovascular coupling and modulate emotional states. **a** KEGG pathway analysis of GluN2D-interacting proteins identified by proteomic analysis of cultured SMCs sorted from P0 brain parenchymal tissue. **b** Immunoprecipitation validation of the interaction between GluN2D and caldesmon in cultured SMCs sorted from P0 brain parenchymal tissue. **c** Experimental schematic for whole-brain (left) and local BLA (right) *Cald1* KD using siRNA. **d**, **e** Representative images (**d**) and quantification (**e**) of caldesmon IF staining. Student’s *t-*test. **f** Electrophoretogram (top) and quantification (bottom) of *Cald1* mRNA expression in cortical lysates following whole-brain *Cald1* siRNA injection. Student’s *t-*test. **g**, **h** Representative IF images of caldesmon in the BLA (**g**) and ventromedial hypothalamus (VMH, **h**) of mice after local BLA *Cald1* siRNA injection. **i** Quantification of caldesmon expression levels from the experiments shown in **g**, **h**. Student’s *t-*test. **j**, **k** Functional hyperemia in the S1BF during whisker stimulation (**j**) and quantification of the plateau phase (**k**). Shaded areas represent SEM across mice. One-way ANOVA followed by Tukey’s multiple comparisons test (*P* = 0.0041). Floxed control mice included mice with cisterna magna scramble siRNA injection (*n* = 3) and mice with local BLA scramble siRNA injection (*n* = 3). **l**, **m** Representative images (**l**) and quantification (**m**) of BLA neuronal activation in the 40-cm OFT. One-way ANOVA followed by Tukey’s multiple comparisons test (*P* < 0.0001). **n** Unsupported rearing bouts in the 40-cm OFT. Kruskal-Wallis test followed by Dunn’s multiple comparisons test (*P* = 0.0001). **o** Serum corticosterone concentrations in the 40-cm OFT. One-way ANOVA followed by Tukey’s multiple comparisons test (*P* = 0.0013). **p**, **q** Defecation bouts (**p**) and defecation rate and latency (**q**) in the 40-cm OFT. Kruskal-Wallis test followed by Dunn’s multiple comparisons test (**p**, *P* < 0.0001**)** and Gehan-Breslow-Wilcoxon test (**q**). Sample sizes (*n*) are indicated between **p**, **q**. Each dot represents one mouse. ****P < 0.0001. Data are presented as mean ± SEM. Detailed statistical data for **q** are shown in Supplementary information, Table S1, sub-table 4. See also Supplementary information, Figs. S14–S15.

Given that direct disruption of the vascular contractile apparatus (*Acta2*-cKO) phenocopies the emotional hyperreactivity of *Grin2d*-cKO mice (Fig. 3; Supplementary information, Figs. S12,S13), we hypothesized that GluN2D couples to the SMC cytoskeletal machinery. To this end, we performed co-immunoprecipitation (Co-IP) followed by mass spectrometry to map the GluN2D interactome in cerebral vascular SMCs. This unbiased screen identified caldesmon (encoded by *Cald1*), an actin-binding protein critical for regulating actomyosin contractility, as a top candidate enriched in the vascular smooth muscle contraction pathway (Fig. 4a).^56,57^ This physical association between GluN2D and caldesmon was further confirmed by western blot analysis of the immunoprecipitated complex (Fig. 4b).

Caldesmon acts as a “blocker” of smooth muscle cell contraction; its disruption is predicted to impair cross-bridge cycling and thereby compromise NVC. To test this, we employed siRNA-mediated knockdown (KD). Unlike GluN2D, caldesmon expression was restricted exclusively to vascular mural cells (Fig. 4c–e),^58^ allowing vascular-specific manipulation without the risk of direct neuronal off-target effects. We validated the efficacy and spatial specificity of this approach: both whole-brain and local BLA siRNA delivery robustly reduced caldesmon expression in background-matched groups (both established in *Grin2d*^*f/f*^ mice) (Fig. 4f–i). Crucially, gene silencing was spatially restricted to the injection site with anatomical precision: local BLA *Cald1* KD did not impact caldesmon levels in the adjacent ventromedial hypothalamus (VMH) (Fig. 4g–i), nor did S1BF-restricted *Cald1* KD alter expression in the BLA (Supplementary information, Fig. S14).

Functionally, local S1BF *Cald1* KD — but not local BLA *Cald1* KD — attenuated whisker-evoked functional hyperemia in the barrel cortex (Fig. 4j, k), confirming *Cald1* KD as an effective tool for disrupting NVC with regional precision. We then focused on the emotional circuit. Behaviorally, both whole-brain and local BLA *Cald1* KD recapitulated the hyperreactive phenotype of *Grin2d*-cKO mice in the 40-cm OFT: *Cald1* KD mice exhibited exaggerated negative emotional responses, characterized by BLA neuronal hyperactivation (c-Fos), reduced unsupported rearing, elevated serum corticosterone concentrations, and intensified defecation dynamics (increased bouts/rate and decreased latency) (Fig. 4l–q). In contrast, local S1BF *Cald1* KD did not alter the above parameters, confirming regional specificity (Supplementary information, Fig. S15).

To elucidate the functional relationship between GluN2D and caldesmon, we applied a local BLA *Cald1* KD approach in *Grin2d*-cKO mice. This epistasis analysis showed no additive phenotype effect (Fig. 4l–q), suggesting that caldesmon likely functions downstream of GluN2D within a hierarchical vascular signaling cascade. Together, these results identify caldesmon as a critical effector linking GluN2D to SMC contractility and demonstrate that localized NVC deficits in the BLA — despite intact NVC in other brain regions — are sufficient to impair emotional responses to mild stress.

### Optogenetic suppression of NVC by BLA arteriolar SMCs precipitates negative emotional states under conditions of mild and negligible stress

To achieve acute suppression of NVC within a millisecond timescale, which is not achievable through genetic or pharmacological manipulations, we employed an SMC-optogenetic approach to reversibly “clamp” vascular diameter.^59^

**Fig. 5:**
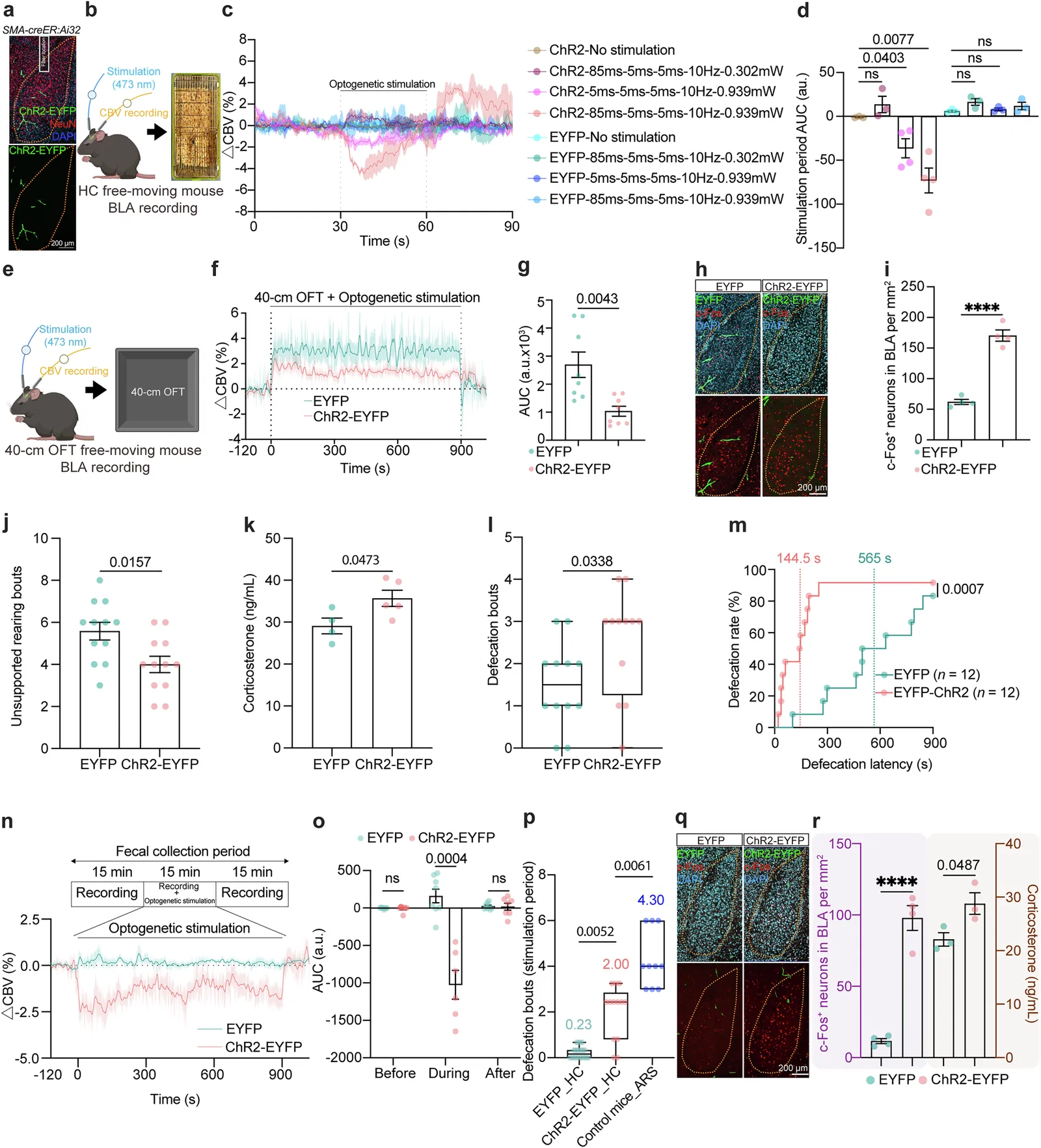
Optogenetic vascular clamping in the BLA is sufficient to drive negative emotionality. **a**, **b** Experimental schematic showing the fiber-optic implantation site (**a**) and the setup for optogenetic stimulation in a freely moving mouse within the home cage (**b**). **c**, **d** Representative CBV traces showing optogenetically induced CBV changes under various stimulation protocols (**c**) and the corresponding quantification (**d**). Each dot represents the area under the curve (AUC) change from one mouse during the stimulation period. Shaded areas represent SEM across mice. One-way ANOVA followed by Tukey’s multiple comparisons test (*P* < 0.0001). **e**–**g** Experimental schematic (**e**), representative CBV traces (**f**), and quantification (**g**) in the 40-cm OFT under optogenetic vascular clamping. Each dot represents the AUC change during optogenetic stimulation from one mouse in **g**. Shaded areas represent SEM across mice in **f**. Dotted lines in **f** indicate *X* = 0 and *Y* = 0. Student’s *t-*test. **h**, **i** Representative images (**h**) and quantification (**i**) of BLA neuronal activation following optogenetic stimulation in the 40-cm OFT. Student’s *t*-test. **j**–**m** Quantification of unsupported rearing bouts (**j**), serum corticosterone concentrations (**k**), defecation bouts (**l**), and defecation rate and latency (**m**) in the 40-cm OFT during optogenetic stimulation. Colored dotted lines in **m** indicate the median defecation latency for each group. Mann-Whitney test (**j**, **l**), Student’s *t-*test (**k**), and Gehan-Breslow-Wilcoxon test (**m**). Sample sizes (*n*) are indicated in **m**. **n**, **o** Experimental schematic of 15-min home cage optogenetic stimulation (**n**, top), the corresponding BLA CBV response (**n**, bottom), and quantification (**o**). Note the extended time scale (15 min) for the duration of optogenetic stimulation. Each dot represents the AUC change during the optogenetic stimulation from one mouse in the home cage. Shaded areas represent SEM across mice. Student’s *t-*test. **p** Quantification of defecation bouts during home cage optogenetic stimulation. Colored numbers indicate the mean number of defecation bouts for each group. Kruskal-Wallis test followed by Dunn’s multiple comparisons test (*P* < 0.0001). **q**, **r** Representative images (**q**) and quantification (**r**) of BLA neuronal activation (**r**, left) and serum corticosterone concentrations (**r**, right) during home cage optogenetic stimulation. Student’s *t*-test. Each dot represents one mouse. ****P < 0.0001. Data are presented as the mean ± SEM. See also Supplementary information, Figs. S16–S17.

We generated SMC-specific ChR2-expressing mice (*SMA-creER;Ai32;* Fig. 5a) and optimized a fiber-optic setup to locally activate BLA SMCs while simultaneously monitoring regional CBV (Fig. 5b). In the home cage under minimal external stress, we optimized a protocol that reliably induced a consistent ~3% CBV decrease (Fig. 5c, red line). This hemodynamic manipulation was rapid and reversible (Fig. 5d).

Consistent with earlier data obtained from *Grin2d*^*f/f*^ mice (Fig. 2i, blue line), stress during the 40-cm OFT elicited functional hyperemia in an independent cohort of *SMA-creER; Ai47* (EYFP) mice (Fig. 5e–g, green line). Optogenetic clamping of BLA arterioles partially blocked this hemodynamic response (Fig. 5e–g, red line). Furthermore, power spectral density analysis of the hemodynamic traces revealed a significant reduction in blood flow power across low-frequency bands — typically associated with vascular vasomotion — during optogenetic stimulation (Supplementary information, Fig. S16). This suppression of spectral energy confirms that optogenetic clamping effectively stabilized vascular diameter.

This acute NVC suppression precipitated an emotional shift: ChR2-EYFP mice exhibited exacerbated negative emotional responses, characterized by markedly increased BLA neural activation (Fig. 5h, i), reduced unsupported rearing (Fig. 5j), and elevated serum corticosterone concentrations (Fig. 5k), as well as altered defecation metrics (Fig. 5l, m) in the 40-cm OFT. These alterations were consistent with the *Grin2d*-cKO phenotype (Fig. 3a–d) in the 40-cm OFT. Locomotor activity and center preference remained unaffected, ruling out motor confounds (Supplementary information, Fig. S17a, b) in the 40-cm OFT. This optogenetically driven, temporally controlled suppression of NVC reveals that preventing the stress-induced vascular surge removes a critical physiological component, thereby amplifying negative emotion.

Next, we investigated whether a BLA-targeted vascular clamp, in the absence of actual external stress, is sufficient to drive stress-like behaviors. We applied the same optogenetic stimulation protocol ( ~3% CBV decrease) to mice in the safe home cage environment for 15 min (Fig. 5n, o). Strikingly, this hemodynamic mismatch alone was sufficient to induce a stress-like state: while basal defecation was negligible in the home cage (0.23 pellets/15 min), optogenetic stimulation induced a cumulative CBV change (Fig. 5o) coupled with a surge in defecation bouts (Fig. 5p). These changes were associated with increased BLA neural activation and elevated serum corticosterone levels (Fig. 5q, r).

Crucially, we verified that this optogenetic manipulation did not induce hypoxic injury. Unlike the Stroke Induced by Magnetic Particles (SIMPLE) model,^60–62^ which triggers robust hypoxia-inducible factor 1-alpha (HIF-1α) upregulation, our protocol elicited no obvious HIF-1α response after optogenetic stimulation in the home cage (Supplementary information, Fig. S17c, d). This confirms that the observed behavioral shifts stem from functional neurovascular modulation rather than ischemic stroke or tissue hypoxia.

### NVC dysfunction, conversely, exhibited hyporeactivity under high-intensity stress

Our findings from the standard 40-cm OFT established that NVC dysfunction precipitates a hyperreactive, anxiety-like state under mild stress (Fig. 3). We next characterized how the NVC-deficient BLA responds to imminent, life-threatening, high-intensity stress.^63^ To this end, we employed two locomotion-triggered paradigms: a looming assay^64^ and a virtual reality (VR_eagle_) simulation of a diving eagle,^65^ both of which mimic an approaching aerial predator.

**Fig. 6:**
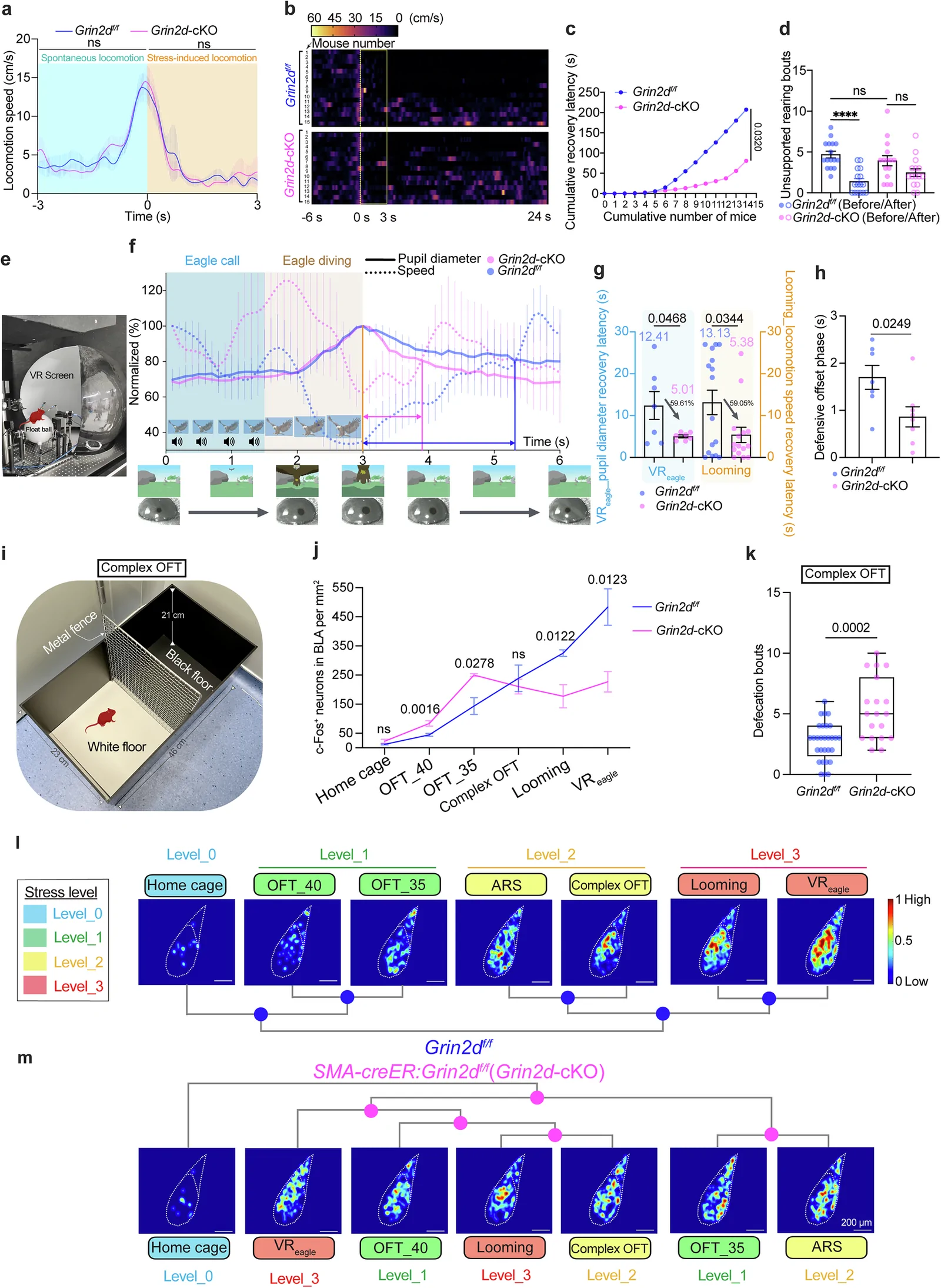
Compromised NVC impairs defensive sustainability and alters BLA neural topological scaling. **a**, **b** Time-series plots (**a**) and heatmaps (**b**) profiling locomotion speed dynamics before (**a**, blue region) and during (**a**, yellow region) looming stimulation. Shaded areas represent SEM across mice in **a**. The yellow dotted box in **b** indicates the 3-s looming duration. **c**, **d** Quantification of cumulative recovery latency from freezing (**c**) and unsupported rearing bouts 5 min before and after looming stimulation (**d**). *n* = 15 mice per group. Kolmogorov-Smirnov test (**c**) and two-way ANOVA on rank-transformed data followed by Sidak’s multiple comparisons test (**d**, *P* < 0.0001). **e** Experimental schematic of the virtual reality facility for visual threat stimulation. **f** Time-series traces showing normalized pupil diameter and locomotion speed during (0–3 s) and after (3–6 s) VR_eagle_ stimulation. The blue region (first 1.5 s) indicates the eagle call, and the yellow region (1.5–3.0 s) indicates the eagle-diving phase. Representative images of the VR_eagle_ stimulus and the mouse eye with a clearly visualized pupil are shown at the bottom. Shaded areas represent SEM across mice. **g**, **h** Quantification of recovery latencies for pupil diameter and locomotion (**g**) from the data in **b**, **f** and defensive offset-phase dynamics (**h**) from the data in **f**. *n* = 7 mice per group in the VR_eagle_ assay and *n* = 15 mice per group in the looming assay. Student’s *t-*test. **i** Experimental schematic of the c-OFT arena. **j** Quantification of BLA neuronal activation in the home cage, 40-cm OFT, 35-cm OFT, c-OFT, looming, and VR_eagle_ contexts. Data from the 40-cm OFT groups are shared with Fig. 2m. Student’s *t-*test. **k** Defecation bouts of mice in the c-OFT. Mann-Whitney test. **l**, **m** SSIM-based unbiased classification of BLA c-Fos topological patterns across seven standard stress contexts in littermate control mice (**l**) and *Grin2d*-cKO mice (**m**). Heatmaps indicate modified c-Fos intensity in the BLA (processing details are provided in Supplementary information, Fig. S19a). ****P < 0.0001. Data are presented as the mean ± SEM. See also Supplementary information, Figs. S18–S19.

Prior to locomotion-triggered looming stimulation, basal locomotor activity was comparable between groups (Fig. 6a, blue region; Supplementary information, Fig. S18a). Upon stimulus presentation, both groups exhibited immediate and robust freezing (Fig. 6a, yellow region), confirming that NVC dysfunction did not impair sensory detection, threat evaluation, or the initiation of defensive strategies.^66^

However, a striking divergence emerged during the maintenance phase: whereas control mice sustained a prolonged freezing state, *Grin2d*-cKO mice exhibited a “premature exit” phenotype (Fig. 6b). Velocity heatmaps confirmed that *Grin2d*-cKO mice resumed locomotor activity significantly earlier than controls (Fig. 6b, c). This behavioral collapse was mirrored by an inappropriate increase in unsupported rearing bouts — a high-risk behavior exposing the animal to recurring predation — immediately following stimulus termination (Fig. 6d). This dissociation between intact initiation and shortened maintenance in freely moving mice underscores the critical role of NVC in sustaining defensive states.

The second life-threatening paradigm, the VR_eagle_ simulation, allowed head-fixed monitoring of pupil diameter in awake mice — a robust index of autonomic arousal^67 ^— alongside locomotor activity on a floating ball (Fig. 6e). Baseline metrics (pupil diameter and locomotor activity) were comparable between genotypes (Supplementary information, Fig. S18b, c). Upon exposure to the eagle call (0–1.5 s; Fig. 6f, blue region) and visual dive (1.5–3 s; Fig. 6f, yellow region), both groups exhibited robust defensive engagement, characterized by immediate freezing and sharp pupil dilation sustained throughout the stimulus (0–3 s; Fig. 6f; Supplementary information, Fig. S18d and Video S2). This confirms that sensory processing and fear initiation remain preserved in *Grin2d*-cKO mice, consistent with observations made in the looming assay.

However, the recovery time for pupil diameter to return to baseline was shortened by ~59% in *Grin2d*-cKO mice (Fig. 6g, left), consistent with the accelerated recovery time of locomotion speed observed in the looming assay (Fig. 6g, right). Collectively, these two independent paradigms demonstrate that NVC-deficient mice cannot physiologically sustain high-load fear states. Moreover, a shortened offset phase emerged in the *Grin2d*-cKO group during the “defensive offset phase”, defined as the interval from stimulus termination to the intersection of pupil diameter and locomotion speed in tracing plots for the recovery period (Fig. 6f, double-arrow lines). While littermate control mice maintained high arousal, *Grin2d*-cKO mice exhibited a rapid collapse of the defensive state (Fig. 6h), with a trend similar to that of the classic recovery time for pupil diameter (Fig. 6g, left).

To establish an intermediate stress condition between the OFT and life-threatening predator scenarios (looming and VR_eagle_), we modified the standard OFT to create a complex OFT (c-OFT). This modification involved the addition of a metal fence barrier that separated an aversive start zone (white floor) from a safe zone (black floor) (Fig. 6i).^29^ c-Fos mapping of littermate controls revealed that the c-OFT paradigm indeed represented an intermediate level of BLA activation, falling between the mild stress elicited by the OFT and the intense stress induced by predator simulation (Fig. 6j, blue line). In contrast, c-Fos mapping in *Grin2d*-cKO mice demonstrated exaggerated neural activation under mild stress yet blunted activation under intense stress, matching their corresponding behavioral phenotypes (Fig. 6j, magenta line).

Notably, under the intermediate c-OFT condition, one-dimensional analysis of c-Fos density failed to correlate with the observed heightened defecation bouts in *Grin2d*-cKO mice (Fig. 6j, k). This discrepancy prompted us to move beyond simple intensity quantification and conduct further analyses of the topological distribution patterns of c-Fos-positive neurons within the BLA (Fig. 6l, m; Supplementary information, Fig. S19).

We therefore decoded c-Fos topology using the structural similarity index measure (SSIM) and unbiased clustering to dissect BLA ensemble configurations (Fig. 6l, m). In littermate controls, BLA topology exhibited dynamic “stress scaling”: activation patterns evolved distinctly from minimal (Level 0) to high-threat (Level 3) states, forming a precise structural hierarchy (Fig. 6l). In contrast, *Grin2d*-cKO ensembles displayed topological disorganization and a lack of hierarchy (Fig. 6m).

Mapping *Grin2d*-cKO neural SSIM signatures against the structural hierarchy of littermate control mice revealed a striking inversion of emotional scaling (Supplementary information, Fig. S19b–g): under mild stress (OFT), *Grin2d*-cKO patterns aberrantly clustered with high-threat control templates (Levels 2 and 3), providing a topological explanation for their hyperreactivity (Supplementary information, Fig. S19d, e). Conversely, under high-load (looming and VR_eagle_) contexts, these patterns failed to scale appropriately and instead regressed to low-stress templates (Level 1), underpinning their functional collapse (Supplementary information, Fig. S19f, g). Specifically, hierarchical clustering revealed that the *Grin2d*-cKO topological signature under c-OFT aberrantly converged with that of control mice under ARS, rather than that of their stage-matched controls. This pathological shift was absent in the home cage, where the topologies of both genotypes remained tightly clustered (Supplementary information, Fig. S19b, c). These findings reveal that BLA NVC deficiency decouples behavior from c-Fos intensity by inducing a nuanced reconfiguration of BLA neural topology.

Collectively, these data reveal a stress load-dependent biphasic pattern (Supplementary information, Fig. S19h): NVC dysfunction precipitates topological hyperreactivity under low-stress-load conditions (OFT) but leads to hyposustainability under high-stress-load conditions (looming and VR_eagle_), demonstrating that functional NVC is indispensable for the topological scaling of neural networks during state transitions.

### Genetic NVC-enhancement model of *Glrb-*cKO mice

Our preceding findings established that the cation-permeable NMDA receptor GluN2D acts as the essential “accelerator” for NVC. Its deficiency disrupts NVC, locking the brain into a state of behavioral rigidity — manifested as hyperreactivity under mild stress yet functional collapse under high-load contexts. Guided by the fundamental principle of electrochemical balance, we hypothesized that if cation influx drives NVC, a converse mechanism mediated by anion channels might functionally restrict hemodynamic gain. Consequently, inhibiting such anionic “brakes” could theoretically potentiate NVC and confer emotional resilience.

**Fig. 7:**
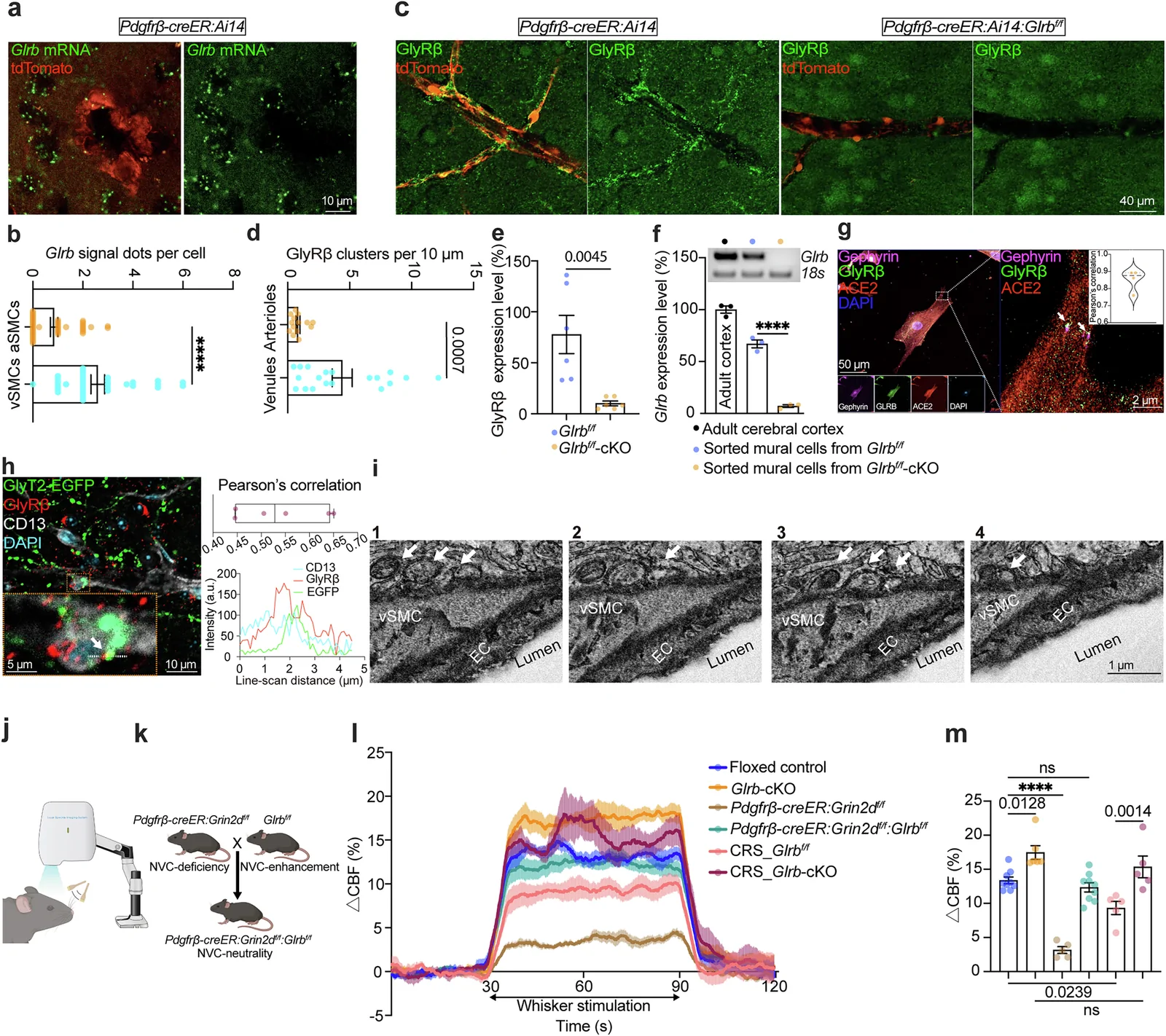
Mural cell-specific GlyRβ deficiency establishes an enhancement model for NVC. **a**, **b** Representative RNAscope images (**a**) and quantification (**b**) of *Glrb* mRNA expression in brain slices. Each dot represents one mural cell; *n* = 4 mice per group. Mann-Whitney test. **c**–**e** Representative images of GlyRβ IF in brain slices (**c**) and quantification in littermate control mice (**d**) and between littermate control and *Glrb*-cKO mice (**e**). In **d**, each dot represents the number of clusters per 10 μm of vessel; *n* = 4 mice per group. In **e**, each dot represents one analysis region of vascular mural cells from a single mouse. Mann-Whitney test (**d**) and Student’s *t-*test (**e**). **f** Electrophoretogram (top) and quantification (bottom) of *Glrb* mRNA expression in acutely sorted Pdgfrβ-positive parenchymal mural cells compared with P60 medullary reticular formation lysates as a positive control. Each dot represents one mouse. One-way ANOVA followed by Tukey’s multiple comparisons test (*P* < 0.0001). **g** Colocalization of GlyRβ with gephyrin in cultured human umbilical venous SMCs (HU-vSMCs). The panel on the right shows Pearson’s correlation analysis. Each dot represents the mean correlation coefficient of ten cells from an independent biological replicate. **h** Colocalization of GlyRβ with glycinergic axon terminals in the cortex of a brain slice. The orange dotted box indicates the magnified area. Top right: Pearson’s correlation analysis. Bottom right: fluorescence intensity line scan across the region indicated by the white arrow. Each dot represents one line-scan region from one mouse. **i** Sequential EM sections of an ascending venule in the mouse cerebral cortex. White arrows indicate structural contacts between axonal terminal and venous SMCs. EC, endothelial cell; vSMC, venous smooth muscle cell. **j**, **k** Experimental schematics of the whisker stimulation paradigm (**j**) and the transgenic mouse mating strategy (**k**). **l**, **m** Functional hyperemia in the S1BF during whisker stimulation (**l**) and quantification of the corresponding plateau phase (**m**). Note that CRS modeling data are also presented here. Shaded areas represent SEM across mice in **l**. Each dot represents one mouse. One-way ANOVA followed by Tukey’s multiple comparisons test (*P* < 0.0001). Control data for *Grin2d*^*f/f*^ and *Pdgfrβ-creER;Grin2d*^*f/f*^ mice are shared with Supplementary information, Fig. S12j. Floxed control mice included comparable *Grin2d*^*f/f*^ mice (*n* = 3), *Glrb*^*f/f*^ mice (*n* = 3), and *Grin2d*^*f/f*^;*Glrb*^*f/f*^ mice (*n* = 3). ****P < 0.0001. Data are presented as the mean ± SEM. See also Supplementary information, Figs. S20–S21.

To identify potential regulators, we screened transcriptomic databases for mural cell-enriched anion channels. This unbiased approach identified *Glrb* (encoding the chloride-permeable glycine receptor subunit beta, GlyRβ) as a top candidate (Supplementary information, Fig. S20).^58^ Spatial mapping via RNAscope and IF confirmed that *Glrb* expression was predominantly enriched in non-arteriolar mural cells (Fig. 7a–d). We validated this expression pattern using *Pdgfrβ-CreER;Glrb*^*f/f*^ (*Glrb*-cKO) mice, which exhibited a significant reduction in *Glrb* (GlyRβ) expression in mural cells (Fig. 7c, e, f). To determine whether these receptors are functionally innervated, we examined their molecular and ultrastructural organization. Molecularly, GlyRβ puncta closely colocalized with gephyrin (Fig. 7g), a scaffolding protein essential for clustering inhibitory receptors,^68^ suggesting the presence of postsynaptic-like machinery. Structurally, confocal imaging and serial electron microscopy (EM) revealed frequent direct physical contacts between neuronal axons and venous mural cells (Fig. 7h, i). Collectively, these molecular and ultrastructural features provide the morphological substrate for potential neuro-mural communication across non-arteriolar segments.

In contrast to the NVC deficit caused by *Grin2d* ablation, *Glrb* deletion induced a “super-coupled” state: *Glrb*-cKO mice exhibited significantly enhanced functional hyperemia upon whisker stimulation (Fig. 7j–m). To pinpoint the cellular origin of this enhancement, we performed a genetic dissection: deletion restricted to arteriolar SMCs (driven by *SMA-creER*) failed to recapitulate the phenotype (Supplementary information, Fig. S21), indicating that the enhanced NVC originates from non-arteriolar mural cell segments.

Given that pharmacological vasodilation with HET0016 successfully rescued the *Grin2d*-cKO phenotype, we hypothesized that genetic removal of *Glrb* could serve as a genetic rescue strategy to counterbalance the *Grin2d* deficit. To test this genetic disinhibition hypothesis, we generated double-knockout mice (*Pdgfrβ-CreER;Grin2d*^*f/f*^*;Glrb*^*f/f*^; Fig. 7k). Remarkably, the concomitant deletion of *Glrb* effectively reversed the NVC deficiency triggered by *Grin2d* loss. Whisker stimulation in *Pdgfrβ-CreER;Grin2d*^*f/f*^*;Glrb*^*f/f*^ mice elicited functional hyperemia responses comparable to those of littermate controls (Fig. 7l, m). This result suggests that *Glrb*-cKO mimics the effect of HET0016. This finding establishes a genetic paradigm of hemodynamic rescue, demonstrating that disinhibition of non-arteriolar mural cells can effectively counterbalance the loss of the arteriolar accelerator. We next asked the fundamental translational question: does this enhancement of neurovascular coupling confer behavioral resilience against stress?

### NVC enhancement confers resilience against both acute and chronic stress

**Fig. 8:**
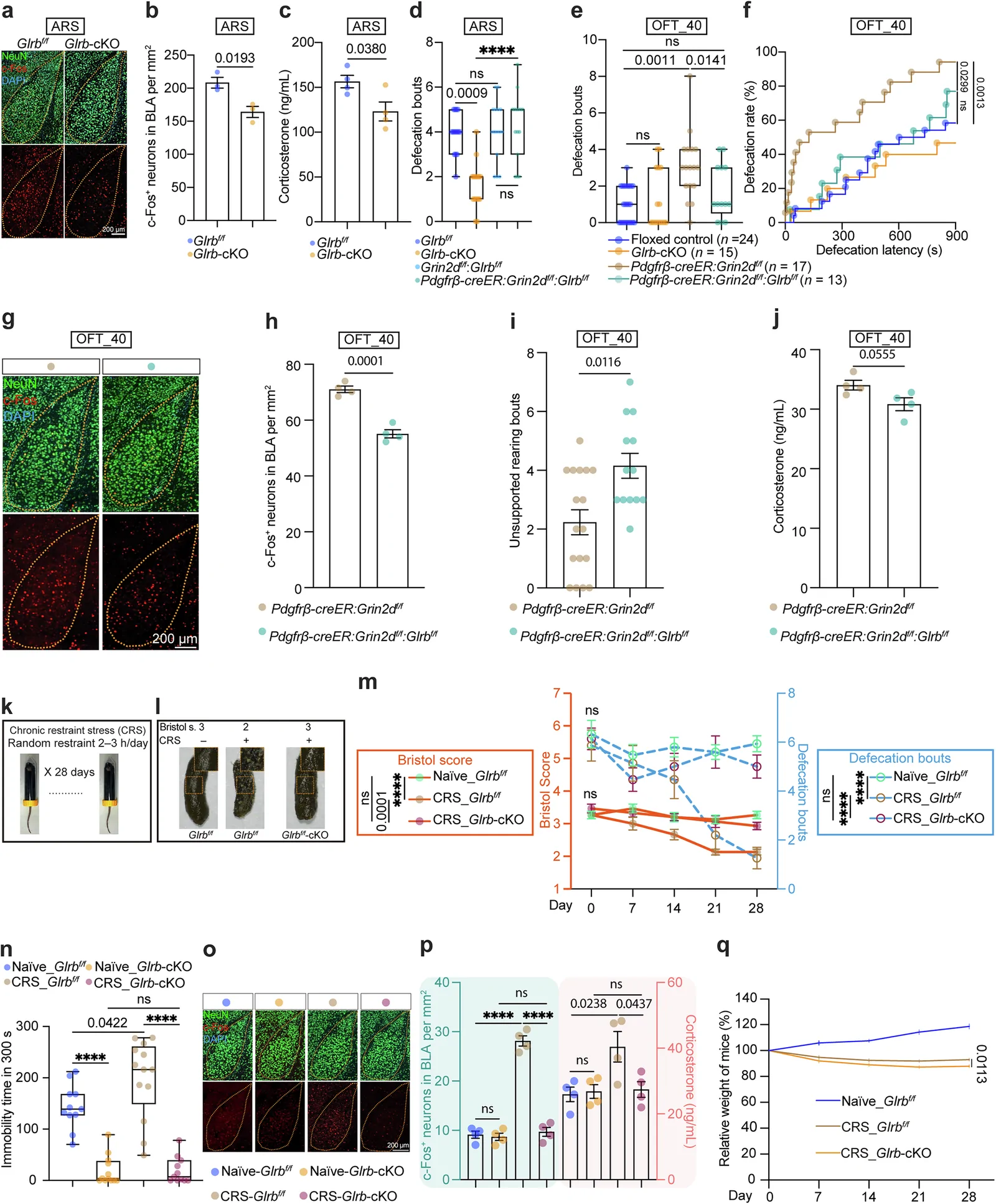
An enhancement model of NVC promotes resilience against acute and chronic emotional distress. **a**, **b** Representative images (**a**) and quantification (**b**) of BLA neuronal activation in ARS. Student’s *t*-test. **c** Serum corticosterone concentrations in ARS. Student’s *t*-test. **d**–**f** Defecation bouts in ARS (**d**) and the 40-cm OFT (**e**) and defecation rate and latency in the 40-cm OFT (**f**). Kruskal-Wallis test followed by Dunn’s multiple comparisons test (*P* < 0.0001 for **d** and *P* = 0.0014 for **e**). Sample sizes (*n*) are indicated in **f**. Gehan-Breslow-Wilcoxon test for **f**. Floxed control data in **e** and **f** (*Grin2d*^*f/f*^ mice (*n* = 14) and *Pdgfrβ-creER;Grin2d*^*f/f*^ mice (*n* = 17)) are shared with Fig. 3b, c and Supplementary information, Fig. S13o, p. Floxed control group in **e** and **f** contains comparable *Grin2d*^*f/f*^ mice (*n* = 14), *Glrb*^*f/f*^ mice (*n* = 5), and *Grin2d*^*f/f*^*;Glrb*^*f/f*^ mice (*n* = 5). **g**, **h** Representative images (**g**) and quantification (**h**) of BLA neuronal activation in the 40-cm OFT. Student’s *t*-test. **i**, **j** Unsupported rearing bouts (**i**) and serum corticosterone concentrations (**j**) in the 40-cm OFT. Mann-Whitney test (**i**) and Student’s *t*-test (**j**). **k** Experimental schematic of the CRS paradigm. **l**, **m** Representative fecal appearance (**l**) and statistical analysis of Bristol scores and defecation bouts during the CRS session (**m**). All fecal scoring was performed by an independent observer blinded to the experimental groups. *n* = 15 mice per group. Two-way ANOVA on rank-transformed data followed by Sidak’s multiple comparisons test (*P* < 0.0001). Bristol s.: Bristol score. **n** Immobility time in the FST after CRS modeling. One-way ANOVA followed by Tukey’s multiple comparisons test (*P* < 0.0001). **o**, **p** Representative images (**o**) and quantification (**p**) of BLA neuronal activation (**p**, left) and serum corticosterone concentrations (**p**, right) after CRS. Quantification was performed by an independent observer blinded to the experimental groups. One-way ANOVA followed by Tukey’s multiple comparisons test (*P* < 0.0001 for **p** left; *P* = 0.0400 for **p** right). **q** Body weight changes during the CRS paradigm. *n* = 15 mice per group. Student’s *t*-test. Each dot represents one mouse. ****P < 0.0001. Data are presented as mean ± SEM. Detailed statistical data for **f** are shown in Supplementary information, Table S1, sub-table 5. Naïve indicates mice without CRS modeling as controls. See also Supplementary information, Figs. S22–S24.

Given that *Glrb* deletion unlocks a “super-coupled” hemodynamic state (Fig. 7), we first asked whether this vascular enhancement mediated by non-arteriolar mural cells translates into immediate behavioral resilience against acute stress. We subjected mice to ARS. While littermate controls exhibited characteristic stress-induced physiological and behavioral shifts, *Glrb*-cKO mice showed markedly stress-buffered responses under ARS: significantly attenuated BLA neuronal activation (Fig. 8a, b) and blunted serum corticosterone concentrations (Fig. 8c). Behaviorally, this physiological resilience was mirrored by stabilized defecation dynamics, with *Glrb*-cKO mice exhibiting significantly fewer defecation bouts under ARS (Fig. 8d).

The observation that *Glrb* removal in mural cells confers stress resilience prompted us to investigate whether this NVC-enhancement model could counteract the deficits caused by *Grin2d*-cKO. We found that *Pdgfrβ-CreER;Grin2d*^*f/f*^*;Glrb*^*f/f*^ mice rescued the core hyper-anxious phenotype observed in *Grin2d-cKO* mutants (Fig. 8d–j). Specifically, *Pdgfrβ-CreER;Grin2d*^*f/f*^*;Glrb*^*f/f*^ mice exhibited defecation metrics comparable to those of NVC-intact littermate controls under ARS and the 40-cm OFT (Fig. 8d–f), accompanied by attenuated BLA neural activation (Fig. 8g, h) and a significant increase in unsupported rearing bouts (Fig. 8i) in the 40-cm OFT. Collectively, the observation that *Pdgfrβ-CreER;Grin2d*^*f/f*^*;Glrb*^*f/f*^ mice closely resemble NVC-intact mice indicates that emotional reactivity is dynamically calibrated by the precise homeostatic balance between opposing vasoconstrictive and vasodilatory signaling pathways within the BLA.

To determine whether the stress resilience observed in systemic mural cell-specific *Glrb*-cKO mice is driven by localized NVC within the BLA, we targeted BLA NVC disruption via local delivery of *Cald1* siRNA. Under intense ARS, local BLA NVC disruption abolished the resilience-conferring effects typically seen in *Glrb*-cKO mice, effectively reverting neural, behavioral, and hormonal stress responses to littermate control levels (Supplementary information, Fig. S22a–d). Furthermore, laser speckle contrast imaging (LSCI) revealed that whole-brain knockdown of *Cald1* via cisterna magna delivery did not alter basal cerebral blood flow (CBF) at the cortical surface (Supplementary information, Fig. S22e). This indicates that *Cald1* deficiency did not compromise basal vascular integrity but specifically impaired the vascular response to neuronal activation.

Although *Glrb* deletion effectively buffers the immediate impact of acute stress, a more rigorous test of neurovascular integrity is whether this protection can be sustained against chronic adversity. We thus escalated our paradigm to chronic restraint stress (CRS, 2–3 h/day for 28 days) (Fig. 8k).^19,69^ We first validated the efficacy of the CRS model in littermate controls, which exhibited a comprehensive state of emotional and physiological exhaustion. Specifically, CRS-treated littermate control mice exhibited significantly attenuated functional hyperemia in the S1BF during whisker stimulation (Fig. 7l, m; floxed control, blue vs CRS_*Glrb*^*f/f*^, red).^19^ Concurrently, these mice developed a distinct constipation-like phenotype, characterized by progressive reductions in both defecation frequency and Bristol scores over the 28-day stress period (Fig. 8l, m). This was accompanied by classic anxiety- and depression-like behaviors, including significantly reduced time in the open arms of the elevated plus maze (EPM; Supplementary information, Fig. S23a), heightened neural activation in the lateral habenula (LHb; Supplementary information, Fig. S23b, c) — a critical node in depression-related circuitry^70 ^— and prolonged immobility in the forced swim test (FST) after CRS modeling (Fig. 8n). Furthermore, we verified that CRS exposure did not compromise BBB integrity (Supplementary information, Fig. S24). This confirms that the observed impairments in NVC and subsequent emotional and behavioral dysregulation after CRS were not confounded by nonspecific vascular barrier disruption.^42,71^

Under this sustained adversity, *Glrb*-cKO mice displayed profound resilience. Specifically, they preserved whisker-evoked functional hyperemia (Fig. 7l, m; *Glrb*-cKO, yellow vs CRS_*Glrb*-cKO, deep purple) and remained resistant to the stress-induced constipation-like phenotype (Fig. 8l, m), maintaining normal defecation dynamics throughout the 28-day CRS period. Furthermore, *Glrb*-cKO mice displayed high mobility in the FST, independent of CRS exposure (Fig. 8n). At the cellular and endocrine levels, *Glrb*-cKO mice were protected from the maladaptive sensitization of stress circuits, showing no significant increase in baseline BLA neural activity or serum corticosterone concentrations compared with CRS-treated littermate controls at the end of CRS (Fig. 8o, p).

Importantly, *Glrb*-cKO mice exhibited normal locomotion and spatial preference in the 40-cm OFT (Supplementary information, Fig. S23d, e), ruling out hyperactivity or altered exploratory drive as confounding factors for the observed behavioral improvements. Finally, it is important to note that *Glrb*-cKO-mediated protection may be brain-region specific. Although emotional and neurovascular homeostasis was preserved, *Glrb*-cKO mice still experienced stress-induced weight loss comparable to that of CRS-treated littermate controls (Fig. 8q). This suggests that while enhanced NVC effectively shielded the brain’s emotional circuits, it did not negate the systemic physiological costs of chronic stress.

## Discussion

In this study, we demonstrate that bidirectional control of NVC enables modulation of negative emotion states. Far from being a passive metabolic read-out of neuronal activity, NVC within the BLA — but not in the primary somatosensory cortex — operates as an indispensable active regulator of the negative affective network. By combining genetic, pharmacological, and optogenetic approaches, we found that the vascular interface acts as a gatekeeper, tuning stress signals to be either amplified into anxiety-like responses under mild stress or dampened to promote resilience under strong stress. These findings redefine BLA NVC as a proactive component of affective circuitry.

More than a century after the first measurements of regional CBF laid the foundation for modern NVC research, the field has come to a new door — what next?^72^ We knock on this door by initially addressing the role of BLA NVC in emotion modulation, offering a conceptual framework for exploring the regulatory capacity of NVC across functionally distinct brain regions and their respective outputs.

During optogenetic manipulations, the ~3% hemodynamic changes we detected (Fig. 5n) are highly consistent with recent work by Hauglund et al.^59^ showing that analogous ~3% CBV changes are sufficient to drive cerebrospinal fluid flow. This concordance across two separate research groups verifies that subtle, persistent shifts in basal blood volume can be sufficient to elicit notable functional effects, for instance, elevated neural activity in the BLA (Fig. 5h, i, q, r, left).

Our arteriolar optogenetic data provide genuine in vivo validation that the NVU acts as a critical regulator of proximal neural activity (Fig. 5e–m). Even under the minimal-stress home cage context with negligible external stress, arteriolar contraction potently activated neighboring neurons (Fig. 5q, r, left), defining an in vivo form of vasculo-neuronal coupling (VNC) that mirrors earlier ex vivo observations.^24^ These findings imply that NVC and VNC operate as two arms of a fast, closed loop, enabling real-time, bidirectional crosstalk between vascular and neuronal compartments.

NVC is a widely recognized, evolutionarily conserved biological process.^73^ The “premature exit” observed in NVC-deficient mice under predator-like stress (Fig. 6) suggests that NVC is innately encoded to optimize the kinetics underlying the predatory fear response cycle. As a survival-critical program, this cycle and its regulatory properties may explain the evolutionary conservation of NVC across species.

The specific mechanism by which *Glrb* deficiency enhances NVC remains to be investigated. In mural cells, the intracellular chloride concentration (Cl^−^_*i*_) is typically higher than the extracellular concentration (Cl^−^_*e*_), creating an electrochemical gradient in which the opening of chloride-permeable glycine receptors leads to Cl^−^ outflow — a sharp contrast to the Cl^−^ influx observed in mature neurons.^74–77^ This efflux is predicted to depolarize mural cells, thereby promoting vascular contraction. Consequently, *Glrb* ablation likely attenuates the contractile tone of non-arteriolar mural cells. Given that *Glrb* is selectively enriched in capillary pericytes and venous smooth muscle cells (vSMCs), but not in arteriolar smooth muscle cells (aSMCs) (Supplementary information, Fig. S20), and that its deletion in aSMCs fails to recapitulate the “super-coupled” phenotype (Supplementary information, Fig. S21), we reasoned that the enhanced NVC in *Pdgfrβ*-targeted mutants may be driven by reduced vasoconstrictor tone normally maintained by pericytes and/or vSMCs. This interpretation aligns with the emerging role of pericytes in modulating capillary diameter and initiating dilatory electrical signals that propagate to upstream arterioles.^78–83^

Although *Grin2d* and *Glrb* primarily modulate distinct vascular segments — the arteriolar and non-arteriolar compartments, respectively — their functional counteraction reveals a sophisticated hemodynamic synergy (Figs. 7–8). These findings imply that non-arteriolar mural cells possess a regulatory capacity within the microvascular network that is functionally comparable to that of arteriolar smooth muscle cells. Our data suggest that NVC operates as an integrated network property in the vascular system, where enhanced non-arteriolar hemodynamics functionally offset arteriolar deficits to maintain an apparent partial equilibrium (Figs. 7, 8).

At the molecular level, we propose that GluN2D activates a signaling cascade in arteriolar SMCs — specifically, a GluN2D-Ca^2+^-bound calmodulin-caldesmon-α-SMA axis. Caldesmon typically binds to α-SMA filaments and sterically blocks their interaction with myosin, thereby suppressing cross-bridge formation.^57^ Ca^2+^-bound calmodulin, generated following Ca^2+^ entry through GluN2D-containing NMDARs, is known to relieve this inhibition.^57^ Consequently, GluN2D activation is predicted to release the caldesmon “brake”, modulating α-SMA-myosin cross-bridge cycling to increase CBF. Although this pathway warrants further direct validation, our prediction is supported by genetic epistasis analysis: *Cald1* KD effectively phenocopies the *Grin2d*-cKO phenotype (Fig. 4l–q), and the lack of an additive effect when combining these two perturbations (*Grin2d*-cKO + local BLA *Cald1* KD) suggests that they potentially operate within the same axis (Fig. 4l–q).

This study has two primary limitations. First, the current lack of CreER lines that can specifically distinguish capillary pericytes and vSMCs necessitated the use of the pan-mural *Pdgfrβ-CreER* driver. Although our genetic exclusion experiments using *SMA-CreER* localized the “super-coupled” phenotype to non-arteriolar segments (Supplementary information, Fig. S21), further refinement using emerging cell-type-specific tools will be necessary to definitively parse the relative functional contributions of these cell types. Second, we utilized male cohorts to minimize hormonal variability; future investigations involving female mice are warranted to establish whether these neurovascular mechanisms generalize in a sex-dependent manner.

In conclusion, we found that NVC functions as an active physiological gatekeeper rather than a passive support system. This study establishes NVC as a key target for basic research into neural circuits and mood disorders.

## Materials and methods

### Animal care and subject selection

All animal procedures complied with the Institutional Animal Care and Use Committee (IACUC) guidelines of the School of Life Sciences, Westlake University (Protocol 24013JJM). Mice were housed in the Westlake University Laboratory Animal Resource Center under strictly controlled conditions: a 12-h light/dark cycle at 25 °C, a maximum of six mice per individually ventilated cage, and *ad libitum* access to standard chow and water. To control for potential confounding effects of the estrous cycle on emotional behavior, we exclusively used age-matched male C57BL/6 J mice (60–80 days old at the onset of experiments).^30^

### Rigor and bias control for behavioral assays

We implemented rigorous protocols to minimize behavioral variability arising from habituation or handling. To ensure stable baseline conditions, animals were first acclimated to the facility for at least 2 weeks and then habituated to the testing room environment for at least 90 min prior to each specific assay. All handling and behavioral assays were performed by a single trained experimenter who was strictly blinded to genotypes and group assignments; blinding was maintained by a separate researcher who managed the genotyping results. Furthermore, testing was confined to a fixed daily time window (13:00–19:00). To prevent exposure to olfactory or auditory cues, testing areas were strictly controlled for light, noise, and odor: apparatuses were thoroughly cleaned with 70% ethanol and ventilated between trials, and animals were transported in standardized containers. Finally, animals from different cages were randomly distributed across experimental groups to control for potential cage effects.^30,84,85^

### Home cage noninvasive physiological monitoring

To assess physiological parameters with minimal disturbance, mice were monitored in their home cages. Blood pressure was measured using a noninvasive tail-cuff system (Kent Scientific, Torrington, CT, USA; BIOPAC Systems, Goleta, CA, USA), and fat content was measured using an EchoMRI^TM^-100H system (EchoMRI, Houston, TX, USA) during the fixed experimental time window (13:00–19:00); crucially, mice were habituated to the restrainer for three consecutive days prior to recording to minimize stress artifacts.

For baseline metabolic and excretory profiling, mice were housed in groups of four. Over three consecutive 3-day cycles (specifically, Days 1–3, 4–6, and 7–9), animals were provided with standardized quantities of food (60 g), water (340 g), and bedding (100 g). At the end of each cycle, body weight, ingestive behaviors (food and water intake), fecal output, and urination volume (derived from bedding weight gain) were recorded. During home-cage baseline assessments, individual excreta could not be definitively assigned under group-housed conditions; thus, data were averaged per mouse within each cage. To ensure statistical rigor and avoid pseudoreplication, the cage mean was utilized as the independent experimental unit (*n* = 4 cages). To facilitate cross-group comparisons, weight data were normalized to the littermate control baseline. Specifically, the value of each sample was divided by the mean value of the control group (defined as 100%). To record defecation bouts, mice were individually housed in fresh home cages for a 3-day habituation period prior to recording. Existing fecal pellets were cleared 15 min prior to the assay, and the number of defecation bouts was recorded during the subsequent 15-min interval. Following monitoring, mice were randomly assigned to terminal procedures, including gastrointestinal examination, retro-orbital blood collection for enzyme-linked immunosorbent assay (ELISA), BBB integrity assessment, and brain extraction for c-Fos IF mapping. All assessors remained blinded to group identity throughout data collection and analysis.

### Behavioral assays

We performed all behavioral assays using a rigorous double-blind protocol. One trained experimenter conducted the assays, while a second independent experimenter performed the video scoring and data analysis.

#### OFT

To assess emotional responses under graded stress intensities, the OFT was conducted in square arenas of three specific dimensions: standard (40 cm × 40 cm), intermediate (35 cm × 35 cm), and constricted (10 cm × 10 cm). Mice were placed in the center of the arena, and behavior was recorded for 15 min using an overhead camera. Unsupported rearing — defined as standing on hindlimbs without contacting the arena walls — was quantified during the first 5 min. Defecation metrics (bouts, rate, and latency), running distance, and area preference were measured across the entire 15-min session. For zonal analysis, the arena floor was digitally divided into 25 equal squares (5 grid × 5 grid); the center zone was defined as the inner 9 squares, excluding the 16 peripheral squares adjacent to the walls.

After testing, mice were transferred to clean, individual cages. Retro-orbital blood samples for ELISA were collected 5 min after the assay. Mice designated for IF were perfused 90 min after the assay to capture peak c-Fos expression. For linear correlation analyses, behavioral and physiological data were paired within the same individuals.

To adhere to the 3Rs principle (Replacement, Reduction, and Refinement) and minimize animal usage, 40-cm OFT data (including whisker stimulation data) for *SMA-creER;Grin1*^*f/f*^ mice were obtained through the retrospective reanalysis of unpublished datasets independently generated in our laboratory. This approach enhances the reproducibility of our findings, as these data were collected by a different experimenter at a distinct time point using identical experimental parameters. Due to the nature of the original experimental design, which was restricted to behavioral videos and whisker-evoked hemodynamic measurements, c-Fos and serum corticosterone data were not available for the *SMA-creER;Grin1*^*f/f*^ line.

For fiber photometry and optogenetic-based OFT, only the 40-cm OFT was used. Tamoxifen induction was performed 21 days prior to formal testing. For real-time NVC monitoring, AAV2/9-hSyn-GCaMP8f was stereotaxically injected into the BLA (AP: −1.6 mm, ML: ±3.45 mm, DV: −4.00 mm), and optic fibers were implanted immediately above the injection site (DV: −3.95 mm). Following a 2-week recovery and three days of fiber-connection habituation, mice received a retro-orbital injection of Rhodamine B (100 μL, 5 mg/mL for formal assay; Cat# R9379; Millipore-Sigma, Burlington, MA, USA) 15 min prior to the 40-cm OFT. The experimental timeline comprised three sequential recording phases: pretest (home cage), test (40-cm OFT), and posttest (home cage).

To rigorously isolate genuine functional signals from hemodynamic and motion-related artifacts, we employed a multi-wavelength fiber photometry system (Thinkertech, Nanjing, China) for all fiber photometry assays, as previously described.^86,87^ Fluorescence signals were acquired via 473-nm excitation for GCaMP8f (Ca^2+^-dependent) and 580-nm excitation for Rhodamine B (blood volume). A 405-nm channel was utilized as an isosbestic reference; at this wavelength, GCaMP8f fluorescence remains Ca^2+^-insensitive, serving as a precise proxy for nonspecific absorption and motion artifacts that affect both the green and red emission spectra. To decontaminate the functional data, the 405-nm reference trace was independently fitted to the 473-nm and 580-nm raw signals using least-squares linear regression. These fitted components were then subtracted from their respective raw traces. This dual-correction strategy effectively neutralized common-mode noise and ensured that the reported △F/F for both GCaMP8f and Rhodamine B reflects true physiological dynamics — neuronal activity and functional blood volume changes, respectively — undistorted by physical movement or nonfunctional optical fluctuations. These data-processing and normalization protocols were consistently applied to optogenetic experiments to ensure cross-experimental comparability (details and the formula shown below).

For optogenetic manipulation of vascular contractility,^43,44,59^ optic fibers were implanted in *SMA-creER;Ai32* (ChR2-EYFP) and control *SMA-creER;Ai47* (EYFP) mice using the coordinates provided above (without AAV2/9-hSyn-GCaMP8f injection). Mice underwent tamoxifen induction 21 days prior to testing. Following habituation and Rhodamine B injection, blue light pulses (473 nm) were delivered to induce optogenetic stimulation while simultaneously monitoring CBV via Rhodamine B fluorescence (580 nm). To capture the full hemodynamic response profile, recordings were segmented into three phases: prestimulation baseline, optical stimulation, and poststimulation recovery. The optogenetic stimulation cycle consisted of an 85-ms stimulation pulse followed by a 5-ms interval and a 5-ms recording window (10 Hz, 0.939 mW laser power).

For isosbestic correction of hemodynamic artifacts, as in BLA photometry recordings, signal fluctuations at the 405-nm isosbestic point are calcium-independent and primarily reflect changes in light scattering and absorption associated with dynamic hemoglobin concentration changes (functional hyperemia). We applied a linear regression correction algorithm. As mentioned above, the 405-nm signal was fitted to the 473-nm signal, and the predicted hemodynamic/motion component was subtracted. This correction confirms that the CBV signal during optogenetic stimulation reflects genuine hemodynamics.

Two distinct normalization strategies were applied to quantify CBV dynamics:

1. Static Baseline Comparison (Cross-group): To allow for cross-group comparisons of basal vascular hemodynamics, data were normalized to the mean raw value of the control group. Specifically, the raw value of each individual sample was divided by the mean raw value of the littermate control group (defined as 100%). This approach standardizes the readout, ensuring that reported values reflect specific genotypic differences in perfusion capacity relative to a physiological baseline.
2. Dynamic Response Quantification (Time-series): For time-series recordings of CBV (as well as Ca^2+^ signal recording and LSCI recording), a temporal normalization strategy was applied. The baseline signal (F_baseline_) was defined as the mean value recorded during the defined prestimulation period (e.g., the 30-s baseline window). The relative change at each time point (F_t_) was calculated as a percentage of its own baseline using the formula:

$$\Delta {{{\rm{CBV}}}}\left( \% \right)\,{{{\rm{or}}}}\,\Delta {{{{\rm{Ca}}}}}^{2+}( \% )=({{{{\rm{F}}}}}_{{{{\rm{t}}}}}-{{{{\rm{F}}}}}_{{{{\rm{baseline}}}}})/{{{{\rm{F}}}}}_{{{{\rm{baseline}}}}}\times 100 \%$$

This self-normalization approach isolates the evoked hemodynamic response from background variations. This peak-aligned averaging strategy ensures that quantification of the metabolic plateau is temporally synchronized to the individual hemodynamic kinetics of each animal.

Fourier transform plots were generated in MATLAB (The MathWorks, Natick, MA, USA), with frequency and power spectral density represented on the *X* and *Y* axes, respectively. The analysis was conducted using the acquisition parameters specified in the 40-cm OFT optogenetic stimulation section. To resolve the spectral characteristics of the BLA hemodynamic response, Fast Fourier Transform (FFT)^88–90^ was applied to the CBV time series. For visualization, the low-frequency range (0–1 Hz) is presented in the representative spectral panel. For statistical quantification, the area under the curve (AUC) was calculated specifically for the ultra-low frequency band (0–0.3 Hz) to represent oscillatory power across experimental groups.^91,92^ The relevant code is available on GitHub: https://github.com/JialabEleven/Functional_hyperemia_regulates_emotion-master/tree/master/Part7_Frequency_analysis.

#### Home cage optogenetic stimulation and recording

To determine whether BLA vasoconstriction is sufficient to induce negative emotion in a nonaversive environment, simultaneous optogenetics and photometry were performed in the home cage. *SMA-creER;Ai32* (ChR2-EYFP) and control *SMA-creER;Ai47* (EYFP) mice underwent tamoxifen induction 21 days prior to testing. Optic fibers were implanted above the BLA (AP: −1.6 mm, ML: ±3.45 mm, DV: −3.95 mm) 2 weeks before the assay, and mice were habituated to the recording tether for three days. The experimental procedures for optogenetic stimulation in the home cage were identical to those conducted in the 40-cm OFT, except for the testing environment.

On the test day, Rhodamine B (100 μL, 5 mg/mL) was administered via retro-orbital injection 15 min prior to recording. The session comprised three 15-min epochs: baseline, stimulation, and recovery. To ensure signal fidelity and prevent spectral crosstalk, an optimized interleaved illumination sequence was employed: (1) 405 nm for isosbestic control, (2) 580 nm for continuous CBV recording, and (3) 473 nm for optogenetic stimulation (delivered exclusively during the second epoch). The duty cycle consisted of an 85-ms stimulation pulse followed by a 5-ms interval and a 5-ms recording window (10 Hz, 0.939 mW laser power). To standardize baseline conditions, all existing fecal pellets were removed immediately before recording, and fresh fecal output was quantified at the end of the session. Data analysis and processing for optogenetic stimulation in the home cage were similar to those used in the 40-cm OFT.

#### Tube stress assay

Restraint stress was induced using modified 50 mL conical tubes (Cat# 430290; Corning, Glendale, AZ, USA).^93,94^ To prevent hypoxia and hyperthermia, tubes were equipped with multiple ventilation apertures drilled into the cap and body, ensuring adequate airflow while strictly restricting physical movement.

For the acute restraint stress (ARS) paradigm, mice were restrained for a single 15-min session, and fecal pellet output was quantified immediately upon release. To interrogate the causal role of BLA activity in stress responses, we utilized a chemogenetic BLA neural silencing strategy. AAV2/5-mCaMKIIa-hM4D(Gi)-EGFP-ER2-WPRE-pA (inhibitory DREADD) or the control virus AAV2/5-mCaMKIIa-EGFP-ER2-WPRE-pA was bilaterally injected into the BLA (AP: −1.6 mm, ML: ±3.45 mm, DV: −4.0 mm). Following a 2-week recovery period, mice were subjected to behavioral testing (including the 40-cm OFT described above). To ensure peak DREADD activation during the stressor, the agonist C21 (0.1 mg/kg, intraperitoneal (i.p.); Cat# HY-100234; MCE, Monmouth Junction, NJ, USA) was administered 45 min prior to initiating restraint. Following ARS, mice were individually housed in clean cages. Retro-orbital blood samples for ELISA were collected 5 min after the assay. To capture stress-induced neuronal activation, mice were perfused 90 min after the assay for c-Fos IF mapping.

For the CRS paradigm, mice were subjected to daily restraint sessions for 28 consecutive days. To prevent habituation to the stressor, both the duration (randomized between 120 and 180 min) and the daily onset time (within the light phase) were varied. The physiological impact of chronic stress was assessed weekly (at the end of each 7-day block) by recording fecal output and stool consistency (Bristol Score)^95^ during a 60-min monitoring period in a clean home cage. Bristol score analysis was conducted by an independent researcher blinded to the mouse genotypes and treatment groups. Mice designated for the EPM test, FST, BBB integrity assessment, ELISA, and IF analysis were sacrificed 24 h after the final CRS session.

#### c-OFT

To simulate an environment of high visual contrast and frustrated escape, the c-OFT was conducted in a specialized chamber divided into two compartments: a high-illumination white zone (aversive) and a low-illumination black zone.^29,96^ Crucially, these zones were separated by a barred metal partition that permitted visual contact with the safe environment but precluded physical entry. Mice were placed in the center of the white compartment at trial onset, and behavioral responses to this context of unattainable safety were continuously recorded for 20 min. Defecation bouts were assessed after the assay. To capture stress-induced neuronal activation, mice were perfused 90 min after the assay for c-Fos IF mapping.

#### Looming assay

Innate defensive behaviors against aerial predators were assessed by adapting established protocols,^64^ with specific optimizations implemented to standardize threat perception. Mice were first acclimated to the arena for 10 min to stabilize baseline anxiety levels. To ensure that defensive responses (freezing or flight) were distinct from baseline immobility or thigmotaxis, a locomotion-dependent triggering protocol was employed. Unlike continuous stimulation paradigms, the looming stimulus — a rapidly expanding overhead black disk — was manually triggered only when a mouse met specific behavioral criteria: spontaneous locomotion (speed 13–15 cm/s) and positioning within the arena center (away from the walls). Behavioral responses were recorded using a high-resolution overhead camera. The baseline of locomotion speed was defined as the mean locomotion speed between 30 s and 10 s before stimulation. Each mouse was subjected to a single stimulation trial to prevent habituation. Immediately following the assay, mice were housed individually in clean cages for 90 min prior to perfusion for c-Fos analysis.

#### Virtual reality eagle (VR_eagle_) assay

To simulate an aerial predator attack within a highly controlled, multimodal environment, we employed a custom-built virtual reality (VR) flight simulation system. The assay design was modified from previous work.^64,65^ The apparatus consisted of an air-supported spherical treadmill (float ball) allowing unrestricted locomotion, surrounded by a panoramic display system to create an immersive visual field. Two weeks prior to testing, mice were implanted with titanium headplates. To strictly decouple restraint stress from predator-induced responses, animals underwent a rigorous daily protocol for habituation to the head-fixation apparatus and the floating ball mechanism (1 week) until they demonstrated stable voluntary locomotion. On the testing day, mice were head-fixed and allowed a 5-min adaptation period with a neutral visual landscape. To ensure capture of active defensive responses, a locomotion-dependent triggering protocol was enforced: the predator stimulus was triggered only after the mouse initiated spontaneous locomotion following the adaptation phase. The stimulus consisted of a high-fidelity eagle avatar diving rapidly from the dorsal to the proximal visual field (3-s duration), temporally synchronized with an eagle call (audiovisual integration, ~75 dB during the first 1.5 s). Pupil diameter was tracked via an infrared camera, and locomotion speed was recorded via rotary encoders sampling the spherical treadmill rotation. Data acquisition continued for 2 min poststimulation to assess recovery kinetics. The baseline locomotion speed and pupil diameter were defined as the mean locomotion speed and pupil diameter between 30 s and 10 s before stimulation. Each mouse underwent a single stimulation trial to prevent habituation. Immediately following the assay, mice were housed individually in clean cages for 90 min prior to perfusion for c-Fos analysis.

#### EPM test

To assess anxiety-like behavior, the EPM test was conducted using an apparatus elevated 50 cm above the floor, consisting of two open arms (65 cm × 5 cm) and two closed arms (65 cm × 5 cm) intersecting at a central platform.^97,98^ At the trial onset, mice were placed in the center zone facing an open arm and allowed to explore freely for 5 min. The duration of time spent in the open arms was recorded.

#### FST

To evaluate behavioral despair, the FST was performed in a transparent glass cylinder filled with water (25 °C) to a depth sufficient to preclude caudal support.^99^ Behavior was recorded for a 5-min session. The duration of immobility — defined as floating with the absence of active struggling and exhibiting only minimal movements necessary to maintain the head above water — was quantified. Immediately after the assay, mice were dried and returned to warmed home cages.

### ELISA

To assess physiological stress levels, blood samples were allowed to clot at controlled room temperature (22–25 °C) for 2 h. Serum was isolated by centrifugation at 3000 rpm for 20 min at 4 °C, followed by a secondary clarification spin at 8000 rpm for 3 min to remove residual debris. Corticosterone concentrations were quantified using a commercial ELISA kit (Cat# abs554164-96T; Absin, Shanghai, China) strictly following the manufacturer’s protocol. Optical density (OD) was measured using a Cytation 5 microplate reader (Model MS-SP104; Agilent, Santa Clara, CA, USA).

### Stereotaxic viral transduction

Mice were anesthetized with isoflurane and secured in a stereotaxic frame. To minimize bias, surgical procedures were performed by investigators who were blinded to the group allocations. For manipulations targeting the BLA, viral vectors were microinjected at the following coordinates relative to bregma: AP: −1.6 mm, ML: ±3.45 mm, DV: −4.0 mm. For chemogenetic inhibition, mice received bilateral injections (150 nL per side) of the neural inhibitory DREADD virus AAV2/5-mCaMKIIa-hM4D(Gi)-EGFP-ER2-WPRE-pA (2.3 × 10^12^ VG/mL) or the control virus AAV2/5-mCaMKIIa-EGFP-ER2-WPRE-pA (2.3 × 10^12^ VG/mL).^37^ For neuronal calcium recording, the calcium indicator AAV2/9-hSyn-jGCaMP8f-WPRE-pA (2.0 × 10^12^ VG/mL) was injected bilaterally (150 nL per side) using the same coordinates. To label glycinergic neurons in the medullary reticular formation, rAAV2/4-Glyt2-EGFP-WPRE-hGHpolyA (5 × 10^12^ VG/mL) was injected bilaterally (500 nL per side) at the coordinates AP: −3.5 mm, ML: ±0.84 mm, DV: −2.8 mm.^100^

Following surgery, mice were allowed to recover for at least 2 weeks to ensure optimal viral expression and physiological recovery before behavioral testing or brain collection.

### IF staining and microscopy

To preserve tissue integrity and antigenicity, mice were deeply anesthetized with 1% pentobarbital sodium (80 mg/kg, i.p.) and transcardially perfused with ice-cold phosphate-buffered saline (PBS; 5 min) followed by 4% paraformaldehyde (PFA) in PBS (5 min). Brains and peripheral organs were harvested, post-fixed in 4% PFA for 6 h at 4 °C, and sectioned at 50 μm using a vibratome (Histo-VT1200S; Leica, Wetzlar, Germany). Cultured cells were washed with PBS and subsequently fixed in 4% PFA for 10 min at room temperature.

For immunolabeling, free-floating sections were permeabilized and blocked in PBS containing 1% Triton X-100 and 0.25% normal donkey serum (NDS) for 60 min at room temperature. Following three washes in PBS, sections were incubated overnight at 4 °C with primary antibodies in a buffer containing 0.1% Triton X-100. After extensive washing, sections were incubated with species-specific fluorescence-conjugated secondary antibodies (in 0.1% Triton X-100) for 2 h at room temperature. Nuclei were counterstained with DAPI (1:1000; Cat# C1002; Beyotime, Shanghai, China). Sections were mounted using anti-fade medium (Cat# 0100-01; SouthernBiotech, Birmingham, AL, USA) and imaged using an LSM800 confocal microscope (Zeiss, Oberkochen, Germany).

To explicitly quantify the spatial distribution of proteins relative to the vasculature (e.g., GlyRβ distribution), orthogonal line-scan profiling was performed. Regions of interest (ROIs) were selected where vessels appeared in longitudinal cross-section in brain slices or at appropriate locations along the boundaries of cultured human umbilical vein smooth muscle cells. Line profiles (5-pixel width) were drawn to capture the fluorescence intensity gradient. In addition, to validate the robustness of these spatial associations and control for selection bias, Pearson’s correlation coefficient (PCC) was calculated across the entire ROI using the ImageJ Coloc 2 plugin.^101^ This global metric confirmed the degree of pixel-by-pixel colocalization between the target protein and the vascular marker, independent of specific line-scan trajectories.

For cellular quantification and normalization, distinct quantification and normalization strategies were applied based on the specific biological metrics:

1. Cellular intensity (e.g., c-Fos): The total number of NeuN-positive nuclei was identified within the defined ROI. Preliminary analysis confirmed that the density of NeuN-positive neurons was consistent across groups, validating the use of area-based normalization. Activation intensity was calculated by dividing the positive neuronal count by the total area of the ROI, expressed as c-Fos-positive neurons per mm^2^. Additionally, to facilitate comparison with alternative quantification methodologies, the proportion of c-Fos-positive neurons relative to the total neuronal population within the BLA and LHb was also calculated. These data are provided in Supplementary information, Fig. S25.
2. Relative protein expression (e.g., GluN2D, HIF-1α, and IgG): Mean fluorescence intensity (MFI) was measured specifically within the somatic ROIs of NeuN-positive neurons. To facilitate cross-group comparisons, data were normalized to the littermate control baseline. Specifically, the MFI of each sample was divided by the mean MFI of the littermate control group (defined as 100%).
3. Signal colocalization (e.g., COX-2): Background fluorescence in the COX-2 channel (546 nm) was first subtracted across the field of view. The colocalization between the COX-2 signal and the NeuN signal (488 nm) within NeuN-positive regions was then quantified using the PCC (Coloc 2 plugin in ImageJ software).

### Pharmacological interventions

To induce Cre-mediated recombination, tamoxifen (Cat# HY-13757A; MCE) was dissolved in corn oil (Cat# C116023; Aladdin, Riverside, CA, USA) at a concentration of 10 mg/mL. The solution was administered via oral gavage (200 μL daily). Dosing schedules were tailored to specific genotypes: *Acta2*-cKO mice were treated from P21 to P24, whereas all other transgenic lines received treatment from P60 to P63, unless otherwise specified.

For acute pharmacological manipulations, two distinct delivery routes were employed:

1. Systemic administration: Different concentrations of HET0016 were administered via i.p. injection at a standardized volume of 200 μL per mouse. Behavioral assays commenced 20 min post-infusion to target the efficacy window.
2. Local BLA injection: To target the BLA specifically, a stainless-steel guide cannula (Cat# 800-00245-00; RWD Life Science, San Diego, CA) was stereotaxically implanted at the coordinates AP: −1.6 mm, ML: ±3.45 mm, DV: −4.0 mm. Drugs were microinfused (1 μL total volume) at a controlled rate of 250 nL/min using an automated syringe pump. HET0016 (10 μM) was microinfused locally into the BLA under a light isoflurane anesthesia. Behavioral assays commenced 5 min post-infusion to target the efficacy window.

### Cell isolation and culture

To characterize SMC-specific molecular mechanisms, primary cultures were established from the SMCs sorted from the P0 brain parenchymal tissue of *SMA-creER;Ai47* mice. Cortical parenchymal tissue was dissected following hypothermia-induced anesthesia and digested in 0.25% trypsin (Cat# 25200072; Thermo Fisher Scientific, Waltham, MA, USA) for 15 min at 37 °C. Digestion was terminated with fetal bovine serum (FBS; Cat# 10099; Thermo Fisher Scientific). The suspension was passed through a 40-μm cell strainer (Cat# 352340; Corning) and centrifuged at 1600 × *g* for 5 min at 4 °C. Pelleted cells were resuspended in Smooth Muscle Cell Medium (SMCM; Cat# 1101; ScienCell, Carlsbad, CA, USA) and plated in T25 flasks (Cat# 430639; Corning).

To purify SMC lineages, primary cultures ( ~80%) were treated with tamoxifen (5 μM in DMSO; Cat# D8371; Solarbio, Beijing, China) to induce EGFP expression specifically in α-SMA-positive cells. Four days post-induction, EGFP-positive cells were purified using an MA-900 cell sorter (FCCF-MBR; Sony, San Jose, CA, USA). Sorted cells were maintained in SMCM for downstream assays. The purity of SMCs isolated via this protocol has been previously validated.^102^

To capture the in vivo transcriptional profile of mural cells (e.g., validation of *Glrb* expression), freshly isolated cells rather than cultured populations were used. Pdgfrβ-positive cells were sorted directly from whole-brain single-cell suspensions dissociated from P60 mice using an anti-Pdgfrβ antibody and immediately lysed in TRIzol reagent (Cat# B511311-0100; Sangon Biotec, Shanghai, China) to minimize transcriptional alterations. For the assay in Fig. 7g, human umbilical vein smooth muscle cells (HU-vSMC; Cat# 8020; ScienCell) were cultured under standard conditions recommended by the manufacturer.

### Semi-quantitative reverse transcription PCR (RT-PCR)

Total RNA was extracted from two distinct sources using TRIzol reagent: (1) cultured cells: sorted EGFP-positive SMCs harvested at ~90% confluence, and (2) brain tissue: cortices or cerebella dissected from P60/P0 mice under 1% pentobarbital sodium (80 mg/kg, i.p.)-induced (P60) or hypothermia-induced (P0) anesthesia.

First-strand cDNA was synthesized (50 ng/μL) using a HiScript III 1st Strand cDNA Synthesis Kit (Cat# R312-02; Vazyme, Jiangsu, China). For transcript abundance analysis, semi-quantitative PCR amplification was performed using Green Taq Mix (Cat# P131-AA; Vazyme). The reaction mixture contained 10 μL Taq Mix, 2 μL cDNA, 2 μL primer pairs (10 μM), and 6 μL RNase-free water. PCR amplicons were resolved on 2% agarose gels (Cat# 111860; BIOWEST, Nuaillé, France) via electrophoresis (220 V for 30 min) and imaged using a Tanon 1600 system (Shanghai, China).

To quantify relative mRNA abundance, the mean gray value of electrophoretogram bands was quantified using ImageJ software: band intensities were first normalized to the corresponding internal loading control (e.g., 18 s) to correct for variations in lane-to-lane loading. Then, to determine relative expression levels across groups, these normalized values were subsequently divided by the mean value of the littermate control group (defined as 100%).

### RNAscope in situ hybridization

To validate cell-type-specific gene expression, 15-μm horizontal brain sections were prepared using a CM1950 cryostat (Leica). Genetically modified lines were utilized to map specific targets: *SMA-creER;Ai14* mice for *Grin2d* expression in arteriolar SMCs and *Pdgfrβ-creER;Ai14* mice for *Glrb* expression in mural cells. WT mice were used for probe specificity validation (*Grin1* and *Grin2d*). Multiplex fluorescent in situ hybridization was performed using the RNAscope Multiplex Fluorescent Reagent Kit v2 (Cat# 323100; ACDbio, Newark, CA, USA), strictly adhering to the published literature and manufacturer guidelines.^38,97^ Specific probes included *Grin2d* (Cat# 425951-C3; ACDbio), *Grin1* (Cat# 431611; ACDbio), and *Glrb* (Cat# 1225261-C1; ACDbio). Spatial quantification, including orthogonal line-scan profiling and colocalization assessment, was performed identically to the IF protocol described above. Briefly, ROIs were selected where vessels appeared in longitudinal cross-section. Line profiles (5-pixel width) were drawn to capture the fluorescence intensity gradient. In addition, to validate the robustness of these spatial associations and control for selection bias, PCC was calculated across the entire ROI using the ImageJ Coloc 2 plugin.

### Western blot analysis

Protein extraction was performed on two distinct sample types: (1) brain tissue: dissected regions were homogenized in ice-cold radioimmunoprecipitation assay (RIPA) buffer (Cat# 89901; Thermo Fisher Scientific) supplemented with a protease inhibitor cocktail (Cat# CW22008; CWBIO, Taizhou, China), and (2) cultured cells: cells were lysed in the same buffer at ~90% confluence. Lysates were centrifuged at 12,000× *g* for 5 min at 4 °C to remove insoluble debris. Protein concentrations were determined using a Micro BCA Protein Assay Kit (Cat# CW2011S; CWBIO). Samples were denatured by heating at 100 °C for 10 min in sodium dodecyl sulfate-polyacrylamide gel electrophoresis (SDS-PAGE) loading buffer. Proteins were resolved on 8% SDS-polyacrylamide gels and transferred onto 0.45 μm polyvinylidene fluoride (PVDF) membranes (Cat# IPVH00010; Millipore-Sigma). Following blocking with 5% nonfat milk in tris-buffered saline with Tween 20 (TBST), membranes were incubated overnight at 4 °C with primary antibodies, washed, and subsequently incubated with HRP-conjugated secondary antibodies for 45 min at room temperature. Immunoreactive bands were visualized using electrochemiluminescence (ECL) reagents (Cat# BMU102-CN; Abbkine, Wuhan, China) and captured on an AI680RGB imaging system (GE Healthcare, Little Chalfont, UK).

To quantify relative protein abundance, the mean gray value of immunoreactive bands was quantified using ImageJ software. A rigorous dual-normalization strategy identical to that in the RT-PCR protocol was applied: band intensities were first normalized to the corresponding internal loading control (e.g., GAPDH) to correct for variations in lane-to-lane loading. Then, to determine relative expression levels across groups, these normalized values were subsequently divided by the mean value of the littermate control group (defined as 100%).

### siRNA administration

To achieve broad cerebrovascular knockdown, siRNA was delivered via injection into the cisterna magna following established protocols.^38^ A mixture containing 0.8 nmol siRNA and fluorescein isothiocyanate (FITC)-dextran (10 mg/mL; Millipore-Sigma) was prepared to allow visual tracking of distribution (20 μL total volume, 1 μL/min infusion rate). At 10 min post-injection, successful delivery was rigorously verified by visualizing perivascular FITC fluorescence through the open scalp using an Axio Zoom V16 microscope (Zeiss). Importantly, only mice exhibiting confirmed widespread perivascular perfusion were included in downstream analyses. Functional outcomes (whisker stimulation and 40-cm OFT) were assessed 48 h post-injection to align with the window of peak knockdown efficiency.

For local BLA and S1BF knockdown, siRNA solution (1 nM, 150 nL) was bilaterally microinjected directly into the BLA at the coordinates AP: −1.55 mm, ML: ±3.40 mm, DV: −4.0 mm or into the S1BF at DV: −0.5 mm. The 40-cm OFT and ARS were conducted 48 h post-injection.

### Whisker stimulation and LSCI

To assess functional hyperemia, mice were anesthetized with 1% sodium pentobarbital (80 mg/kg, i.p.). The assay design was as described previously.^38,50,62^ Following anesthesia, the head was secured in a stereotaxic frame, and the scalp was retracted to expose the skull. We stimulated whiskers longitudinally using a motor‑driven brush (12 Hz, 1 min) and recorded CBF dynamics in the contralateral barrel cortex using an LSCI system (RFLSI ZW/RFSLI III, RWD Life Science).^103^ Images were acquired at a resolution of 512 pixels × 512 pixels with an exposure time of 5 ms, optimized for 10 frames per second (fps) sampling. Each LSCI recording session spanned 120 s, comprising a 30-s prestimulation baseline, a 60-s whisker stimulation period, and a 30-s poststimulation recovery. For HET0016-related experiments, a sequential within-animal design was employed: mice were first administered saline (200 µL, i.p.), followed by HET0016 (1 mg/kg, 200 µL, i.p.) after a 1-h interval to allow for physiological recovery. LSCI acquisition commenced 20 min after each injection, facilitating rigorous paired statistical analysis.

The LSCI system provides data in “Perfusion Units (PU)”, which represent a relative blood flow index derived from the spatial blurring of laser speckle patterns. This index is related to the product of the average speed and concentration of moving red blood cells in the tissue sample volume. While these raw PU values were used to compare basal perfusion levels between groups, functional hyperemia data were normalized to correct for interindividual variations in cranial optical properties. Specifically, the CBF response was calculated as the percentage change relative to baseline:

$$\Delta {{{\rm{CBF}}}}( \% )=({{{{\rm{CBF}}}}}_{{{{\rm{t}}}}}-{{{{\rm{CBF}}}}}_{{{{\rm{baseline}}}}})/{{{{\rm{CBF}}}}}_{{{{\rm{baseline}}}}}\times 100 \%$$

where CBF_t_ is defined as the perfusion value of each frame and CBF_baseline_ is defined as the mean perfusion value during the 30‑s prestimulation period.

To characterize the temporal dynamics of functional hyperemia, the peak response time was identified using a custom MATLAB algorithm (Signal Processing Toolbox). Specifically, local maxima in the △CBF time-series were detected using the findpeaks function, with empirical constraints applied to reject signal noise (minimum peak height threshold > 2.2%; minimum peak separation > 50 frames). The global maximum detected within the stimulation window was defined as the peak functional hyperemia. To quantify the magnitude of the sustained vascular response, we calculated the mean △CBF over a period from the identified peak latency to 90 s (which marks the conclusion of the whisker stimulation). Statistical analyses of baseline cerebral perfusion, calculated as the mean value during the 30-s prestimulation period across all LSCI datasets, are summarized in Supplementary information, Fig. S11.

### Tissue clearing and 3D vascular reconstruction

To visualize the 3D vascular architecture, mouse brains were washed in PBS (3 h × 2 h) on a shaker (40 rpm) at room temperature. Tissue clearing was performed using a commercial kit (Cat# NH-CR-210701; Nuohai, Shanghai, China), adhering strictly to the manufacturer’s protocol. Cleared whole-brain samples were imaged using light-sheet fluorescence microscopy (LSFM; LS18, Nuohai) with a 561-nm excitation laser. For structural analysis, raw datasets were stitched using LS18 software and downsampled (0.25×) in ImageJ.

The datasets, comprising 1500 sagittal slices, were registered to the Allen Mouse Brain Common Coordinate Framework (CCFv3) using the BIRDS plugin. Specifically, nine reference slices were selected, and their corresponding atlas indices were manually defined to guide the registration algorithm. The automated registration for the entire dataset was then executed based on these reference points. To ensure spatial accuracy, each slice was monitored during processing, followed by randomized spot checks post-registration. Any misalignments were resolved through manual adjustment or by re-running the registration pipeline. Based on this refined registration, Imaris Surface modules were generated for specific brain regions (e.g., BLA). Vascular structures were segmented based on fluorescence signal intensity, and vascular volume was quantified using Imaris software.

### HFD challenge

To evaluate NVC integrity under clinically relevant metabolic stress, a diet-induced obesity model was employed. Mice were randomized into HFD (60% kcal from fat; Cat# PD6001; SYSE Bio-Technology, Jiangsu, China) or normal fat diet control groups for 4 weeks. Body weight and food intake were monitored weekly. The physiological impact was assessed by quantifying home-cage fecal output as described above. Following the 4-week modeling period, mice underwent the 40-cm OFT and whisker stimulation assays to assess emotional and hemodynamic function, respectively. For ELISA, serum was collected 5 min after the 40-cm OFT. For c-Fos IF staining, brain tissues were harvested 90 min after the 40-cm OFT.

### IP assay

To map the GluN2D interactome, cultured SMCs sorted from P0 *SMA-creER;Ai14* mouse brain parenchymal tissue (via tdTomato-based sorting) were collected and subsequently expanded in SMCM. At ~90% confluence, cells were transduced with lentivirus encoding Grin2d-3XFlag (*Lenti-EF1-EGFP-CMV-Grin2d-3XFlag-WPRE*, 5 × 10^12^ VG/mL). One week post-transduction, infected cells (EGFP-tdTomato double positive) were purified using an MA-900 sorter and expanded in 10-cm dishes. Upon reaching 90% confluence, cells were lysed in RIPA buffer. Lysates were incubated with anti-GluN2D or IgG control antibodies conjugated to Protein G Magnetic Beads (Cat# YJ002; Epizyme, Cambridge, MA, USA) to pull down the protein complex. Eluates were analyzed via western blot and mass spectrometry.

### BBB permeability assay

To evaluate BBB integrity, the Evans Blue dye extravasation assay was performed.^104^ Mice were injected retro-orbitally with 2% Evans Blue (4 μL/g; Cat# E2129; Millipore-Sigma (Merck)) dissolved in saline. At 45 min post-injection, mice were anesthetized, and whole brains were harvested. Macroscopic dye leakage was first assessed qualitatively using an Axio Zoom V16 microscope. For quantitative assessment, brains were weighed and homogenized in 1 mL of 50% trichloroacetic acid (Cat# 190780; Millipore-Sigma (Merck)). Following centrifugation at 10,000× g for 20 min at 4 °C, the supernatant was collected, and the Evans Blue concentration was quantified by measuring absorbance at 620 nm. To detect microscopic leakage, IF staining for immunoglobulin G (IgG) was performed as described in the IF staining section. We utilized brain sections from mice subjected to nonperfusion as positive controls — defining their mean fluorescence intensity as the 100% benchmark — to quantify the IgG intensity.

### Colorectal endoscopy

To assess stress-induced gastrointestinal pathology, representative images of the distal colon were captured using the ENDOQ colonoscopy system (KU Leuven, Leuven, Belgium). To ensure unbiased evaluation, a gastroenterologist blinded to the experimental groups scored the mucosal morphology and signs of inflammation based on the endoscopic images.

### Hematoxylin and eosin (H&E) staining

For histological analysis, mice were anesthetized and perfused as described above. Harvested tissues were post-fixed in 4% PFA for 6 h at 4 °C. Following standard paraffin embedding, 8-μm sections were cut (RM2255 Histo. Microtome; Leica, Wetzlar, Germany), and H&E staining was performed to visualize gross morphological structures.

### Topological characterization of BLA activation patterns

To objectively classify emotional states based on the spatial configuration of neuronal ensembles, a topology-based estimation framework was developed, integrating the SSIM with unsupervised hierarchical clustering.

To transition from discrete cellular signals to a continuous representation of regional activation, raw c-Fos IF staining images were preprocessed in MATLAB. Images were converted to grayscale, inverted, and normalized to a (0, 1) intensity scale. A multi-scale Gaussian smoothing protocol (σ1 = 6 pixels, σ2 = 12 pixels) was applied to generate a continuous spatial intensity map representing the macro-scale topography of neuronal activation within the BLA. This step effectively minimizes high-frequency noise from individual cell variations while preserving the global spatial structure.

To quantify the topological similarity between experimental conditions, pairwise SSIM values were computed between the activation intensity maps. Unlike traditional metrics that rely on absolute cell counts or pixel-by-pixel subtraction, SSIM assesses the preservation of structural information by integrating comparisons of luminance (*l*), contrast (*c*), and structure (*s*). The similarity index between image *x* and *y* was calculated as:

$${{{\rm{SSIM}}}}(x,y)={(l(x,{y}))}^{{{{\rm{\alpha }}}}}{{{\rm{\cdot }}}}{(c(x,y))}^{{{{\rm{\beta }}}}}{{{\rm{\cdot }}}}{(s(x,y))}^{{{{\rm{\gamma }}}}}$$

This metric provides a robust measure of spatial fidelity, converting image-based topographical correlations into a numerical similarity matrix.

The resulting SSIM similarity matrix was utilized for cluster analysis in R. We defined the topological distance (*D*) between any two conditions as *D* = 1 - SSIM. Based on this distance matrix, unsupervised hierarchical clustering was performed using Ward’s minimum variance method (method = “ward.D2”). This algorithm minimizes total within-cluster variance to construct a dendrogram, effectively grouping behavioral contexts into distinct clusters based purely on topological proximity. These naturally emerging clusters were then mapped to discrete stress levels (Levels 0–3), defining the subject’s state based on the intrinsic topology of its neuronal response rather than arbitrary thresholds.

To ensure rigor, a double-blind workflow was implemented: image processing and algorithmic analysis were conducted by two independent analysts, distinct from the personnel performing wet-lab experiments. Statistical analyses were performed using GraphPad Prism (v9.4.1; GraphPad Software, San Diego, CA) and custom MATLAB scripts. All source code is available at: https://github.com/JialabEleven/Functional_hyperemia_regulates_emotion-master.

### Serial block-face scanning electron microscopy (SBF-SEM)

To visualize the ultrastructure of the neurovascular interface, we prepared specimens according to our previous protocols.^38^ Briefly, we identified ascending venules using X-ray microscopy (Xradia 520 Versa, Zeiss) at 4× magnification (80 kV). We mounted the specimen on an aluminum pin using conductive epoxy (Cat# CW2400; Chemtronics, Kennesaw, GA, USA) and trimmed it into a 600 × 600 × 700 μm^3^ block using a glass knife and a diamond trimming knife (Trim 90, DiATOME, Biel, Switzerland). Prior to imaging, we sputter-coated the sample with gold-palladium (EM ACE200, Leica). We acquired images using a BSE detector on a Gemini 300 scanning electron microscope (Zeiss) equipped with a 3View ultramicrotome (Gatan, Pleasanton, CA, USA). Imaging parameters were set to 2 kV acceleration voltage under high vacuum ( ~ 1.8 × 10^–3 ^mbar) with focal charge compensation. We performed serial sectioning at a 50–75 nm thickness with a cutting speed of 0.1 mm/s.

### Stroke modelling

To validate the absence of hypoxic injury in our optogenetic model, we generated a positive control for hypoxia using the SIMPLE method.^60–62^ Briefly, we injected magnetic microparticles into the bloodstream and positioned a magnet over the MCA to induce targeted occlusion. This permanent ischemia triggers robust upregulation of HIF-1α within 24 h. We utilized brain sections from mice subjected to the SIMPLE stroke model as positive controls — defining their mean fluorescence intensity in the stroke infarct area as the 100% benchmark — to quantify HIF-1α expression.

## Supplementary information

Supplementary information, Video S1

Supplementary information, Video S2

Supplementary information, Figure S1

Supplementary information, Figure S2

Supplementary information, Figure S3

Supplementary information, Figure S4

Supplementary information, Figure S5

Supplementary information, Figure S6

Supplementary information, Figure S7

Supplementary information, Figure S8

Supplementary information, Figure S9

Supplementary information, Figure S10

Supplementary information, Figure S11

Supplementary information, Figure S12

Supplementary information, Figure S13

Supplementary information, Figure S14

Supplementary information, Figure S15

Supplementary information, Figure S16

Supplementary information, Figure S17

Supplementary information, Figure S18

Supplementary information, Figure S19

Supplementary information, Figure S20

Supplementary information, Figure S21

Supplementary information, Figure S22

Supplementary information, Figure S23

Supplementary information, Figure S24

Supplementary information, Figure S25

Supplementary information, Table S1

Supplementary information, Table S2

Supplementary information, Table S3

Supplementary information, Table S4

Supplementary information, Table S5

## Data Availability

Detailed information on the mice, antibodies, and primers used in this study is provided in Supplementary information, Tables S3, S4, S5, respectively. Fluorescence images were processed using Zen Lite (v3.4; Zeiss) and ImageJ (v1.53 u). For fiber photometry, signal demodulation and linear regression correction were performed using the system’s built-in proprietary software (Thinkertech). Whisker stimulation data were acquired and analyzed using the LSCI software (v5.0; RWD Life Science). Trajectory tracking for the OFT, c-OFT, and EPM test was performed using the EthoVision XT system (v18; Noldus, Wageningen, the Netherlands), which was also utilized to generate locomotion heatmaps. For the Looming and VR_eagle_ assays, tracking was conducted using custom scripts (Bayonne). For the physiological and topology analysis, custom MATLAB scripts were developed to analyze CBF peak kinetics, map aggregate defecation coordinates, and generate neural activation heatmaps for the topology-based estimation framework. Kyoto Encyclopedia of Genes and Genomes (KEGG) pathway enrichment analysis and SSIM-based hierarchical clustering were executed using custom R algorithms. Statistical analyses were conducted using GraphPad Prism (v9.4.1) and MATLAB. Figures were assembled using Illustrator 2024 (v28.3; Adobe, San Jose, CA, USA). Outliers were identified and excluded using the interquartile range (IQR) method (values falling outside the range of Q1 — 1.5 × IQR to Q3 + 1.5 × IQR).^105^ Data are expressed as mean ± standard error of the mean (SEM). The normality of data distribution was assessed using the Shapiro-Wilk test. Specific statistical tests employed for each experiment are detailed in the corresponding figure legends. Exact *P* values are reported to four decimal places. A *P* value < 0.05 was considered indicative of statistical significance. A comprehensive data resource checklist, detailing figure attributions and sample sources, is provided in Supplementary information, Table S2 for reference. Furthermore, to ensure maximum transparency and statistical robustness, the percentage of c-Fos-positive neurons in the BLA across all experimental cohorts is quantified and presented in Supplementary Fig. S25. All custom MATLAB and R code generated for the topology-based estimation framework and hemodynamic analysis is available on GitHub: https://github.com/JialabEleven/Functional_hyperemia_regulates_emotion-master.
