# Supplementary material for: Neurovascular coupling in the basolateral amygdala modulates negative emotions
Source: Cell Res. 2026 May 27;36(9):656–79. doi: 10.1038/s41422-026-01256-2 (PMC13503848; doi:10.1038/s41422-026-01256-2)
Supplement: Supplementary file 1 [file 41422_2026_1256_MOESM10_ESM.pdf]

Figure S8

Ruan et al.

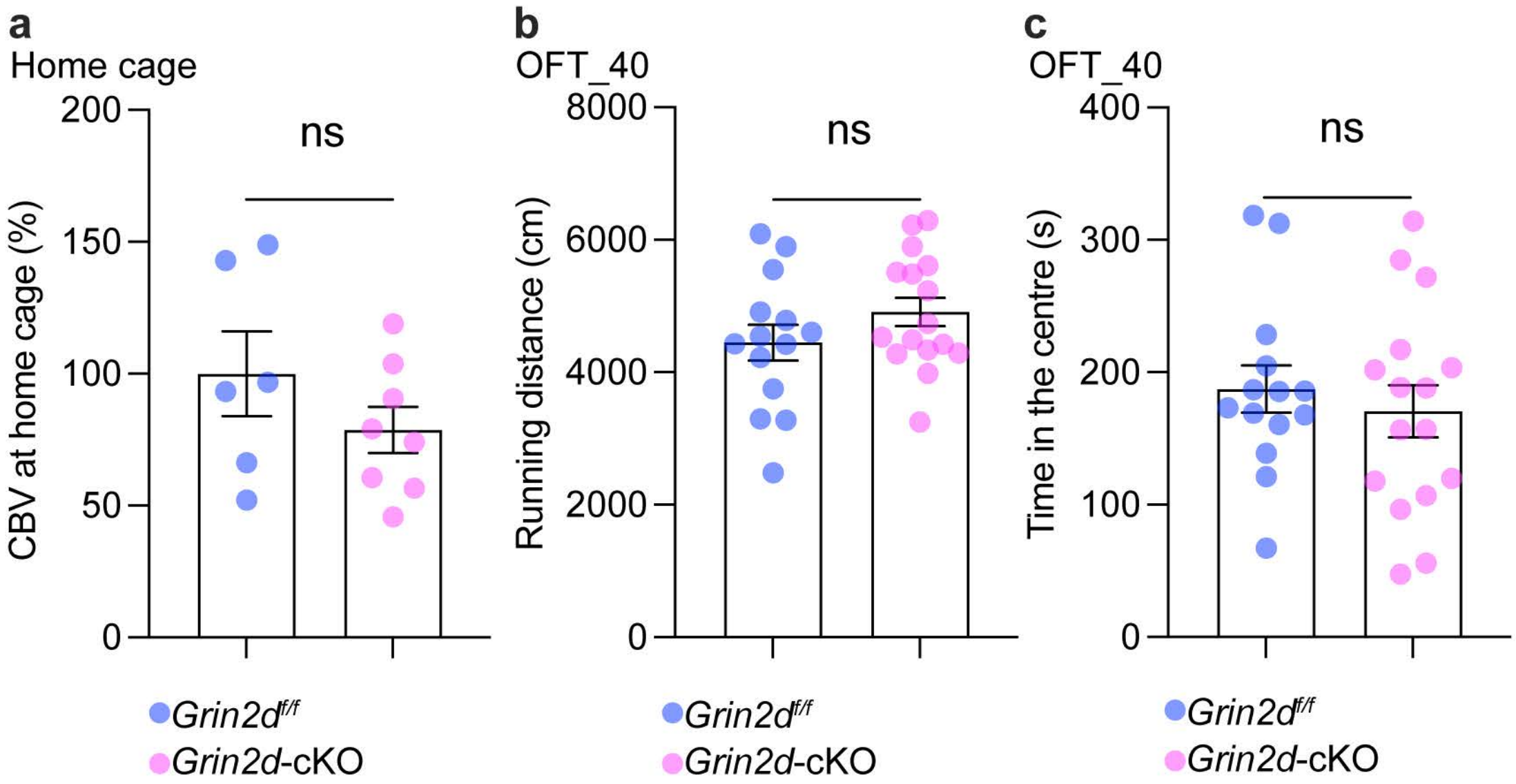

Fig.S8 Baseline CBV and OFT performance in *Grin2d*-cKO mice. a Baseline CBV in the BLA during the home cage stage. Each dot represents one mouse. Student's *t*-test. **b, c** Running distance (**b**) and time spent in the center zone (**c**) in the 40 cm OFT. Each dot represents one mouse. Student's *t*-test. Data are presented as mean ± SEM.
