# Supplementary material for: Neurovascular coupling in the basolateral amygdala modulates negative emotions
Source: Cell Res. 2026 May 27;36(9):656–79. doi: 10.1038/s41422-026-01256-2 (PMC13503848; doi:10.1038/s41422-026-01256-2)
Supplement: Supplementary file 2 [file 41422_2026_1256_MOESM11_ESM.pdf]

Figure S9

Ruan et al.

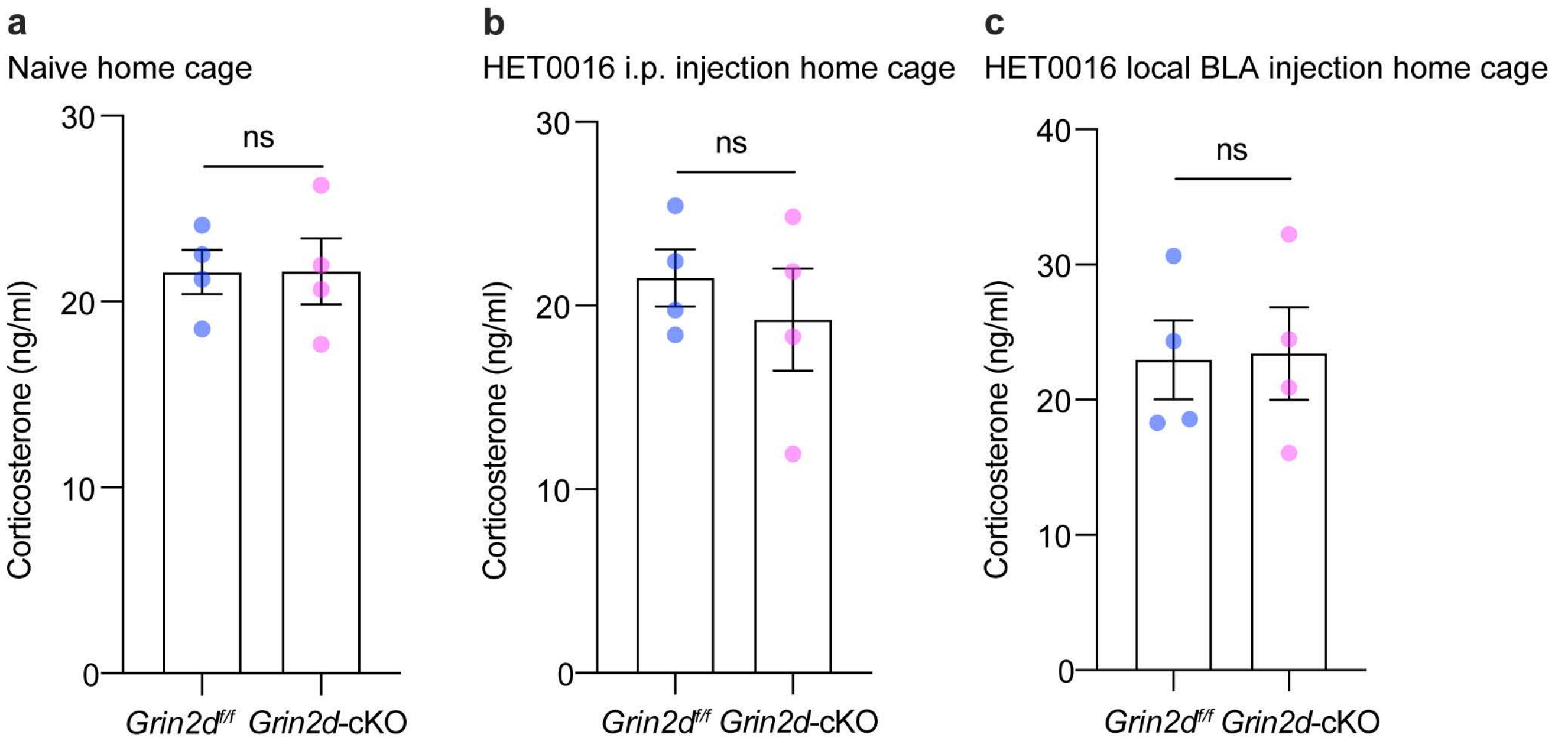

Fig.S9 Serum corticosterone concentrations under baseline conditions. a-c Serum corticosterone concentrations in mice at the home cage condition (**a**), following i.p. injection of HET0016 (**b**), and following local BLA microinjection of HET0016 (**c**). Each dot represents one mouse. Student's *t*-test. Data are presented as mean ± SEM.
