# Supplementary material for: Neurovascular coupling in the basolateral amygdala modulates negative emotions
Source: Cell Res. 2026 May 27;36(9):656–79. doi: 10.1038/s41422-026-01256-2 (PMC13503848; doi:10.1038/s41422-026-01256-2)
Supplement: Supplementary file 3 [file 41422_2026_1256_MOESM12_ESM.pdf]

Figure S10

Ruan et al.

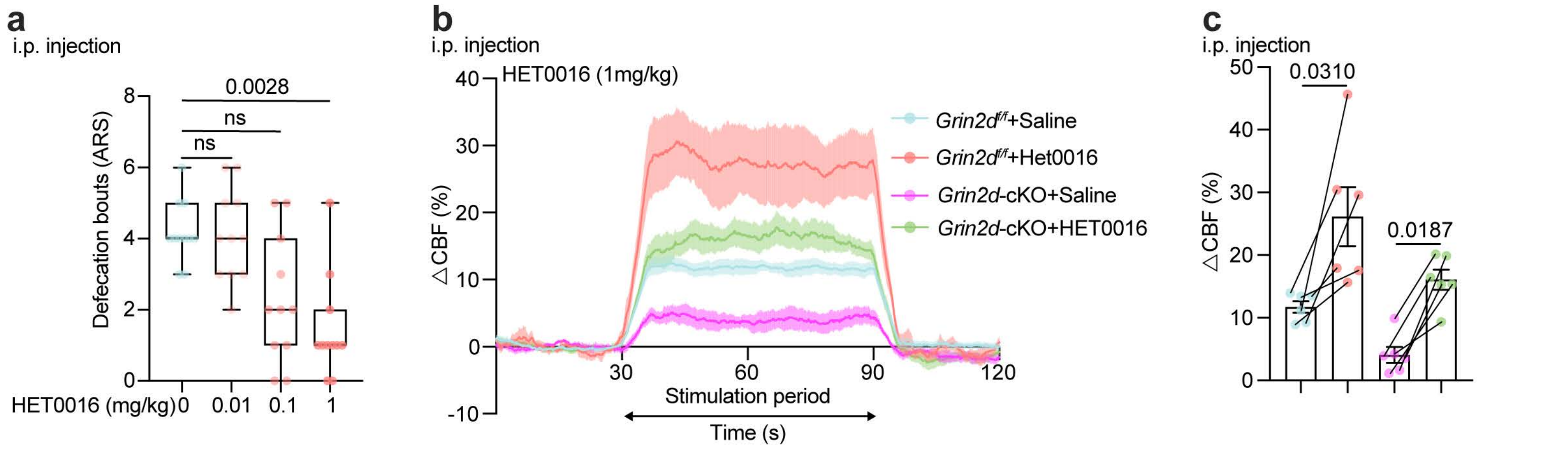

Fig.S10 Dose-dependent and functional validation of HET0016. a Effects of different HET0016 concentrations on defecation bouts in ARS. Each dot represents one mouse. Kruskal-Wallis test followed by Dunn's multiple comparisons test (P = 0.0005). **b, c** Functional hyperemia in the S1BF during whisker stimulation (**b**) and quantification of the corresponding plateau phase (**c**). Shaded areas represent SEM across mice. Each dot represents one mouse. Paired Student's *t*-test. Data are presented as mean ± SEM.
