# Supplementary material for: Neurovascular coupling in the basolateral amygdala modulates negative emotions
Source: Cell Res. 2026 May 27;36(9):656–79. doi: 10.1038/s41422-026-01256-2 (PMC13503848; doi:10.1038/s41422-026-01256-2)
Supplement: Supplementary file 4 [file 41422_2026_1256_MOESM13_ESM.pdf]

Figure S11

Ruan et al.

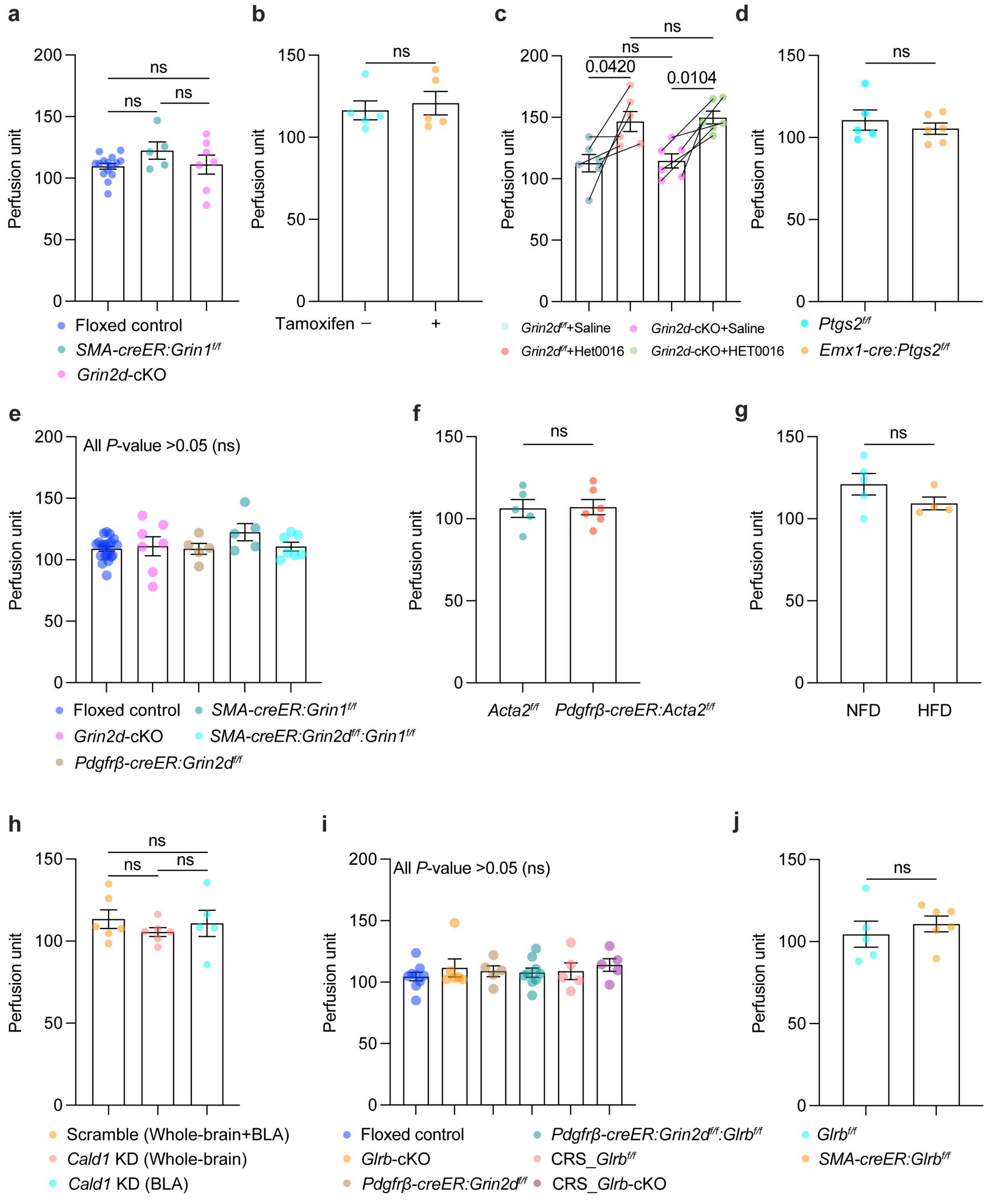

Fig.S11 Comprehensive quantification of baseline cerebral perfusion across all LSCI datasets. a-j Statistical analysis of baseline mean cerebral perfusion corresponding to the original data presented in Fig. 2g (**a**), Fig. S5a (**b**), Fig. S10b (**c**), Fig. S12d (**d**), Fig. S12j (**e**), Fig. S12u (**f**), Fig. S13g (**g**), Fig. 4j (**h**), Fig. 7l (**i**), and Fig. S21a (**j**). Sample sizes (*n*) for each cohort are identical to those indicated in the respective original panels. Student's *t*-test (**b**, **d**, **f**, **g**, **j**), paired Student's *t*-test (**c**), or One-way ANOVA followed by Tukey's multiple comparisons test (**a**, **e**, **h**, **i**). *P*-values for One-way ANOVA are indicated as follows: **a**, $P = 0.2225$; **e**, $P = 0.3025$; **h**, $P = 0.5480$; **i**, $P = 0.4447$. Each dot indicates one mouse. Data are presented as mean ± SEM.
