# Supplementary material for: Neurovascular coupling in the basolateral amygdala modulates negative emotions
Source: Cell Res. 2026 May 27;36(9):656–79. doi: 10.1038/s41422-026-01256-2 (PMC13503848; doi:10.1038/s41422-026-01256-2)
Supplement: Supplementary file 5 [file 41422_2026_1256_MOESM14_ESM.pdf]

Figure S12

Ruan et al.

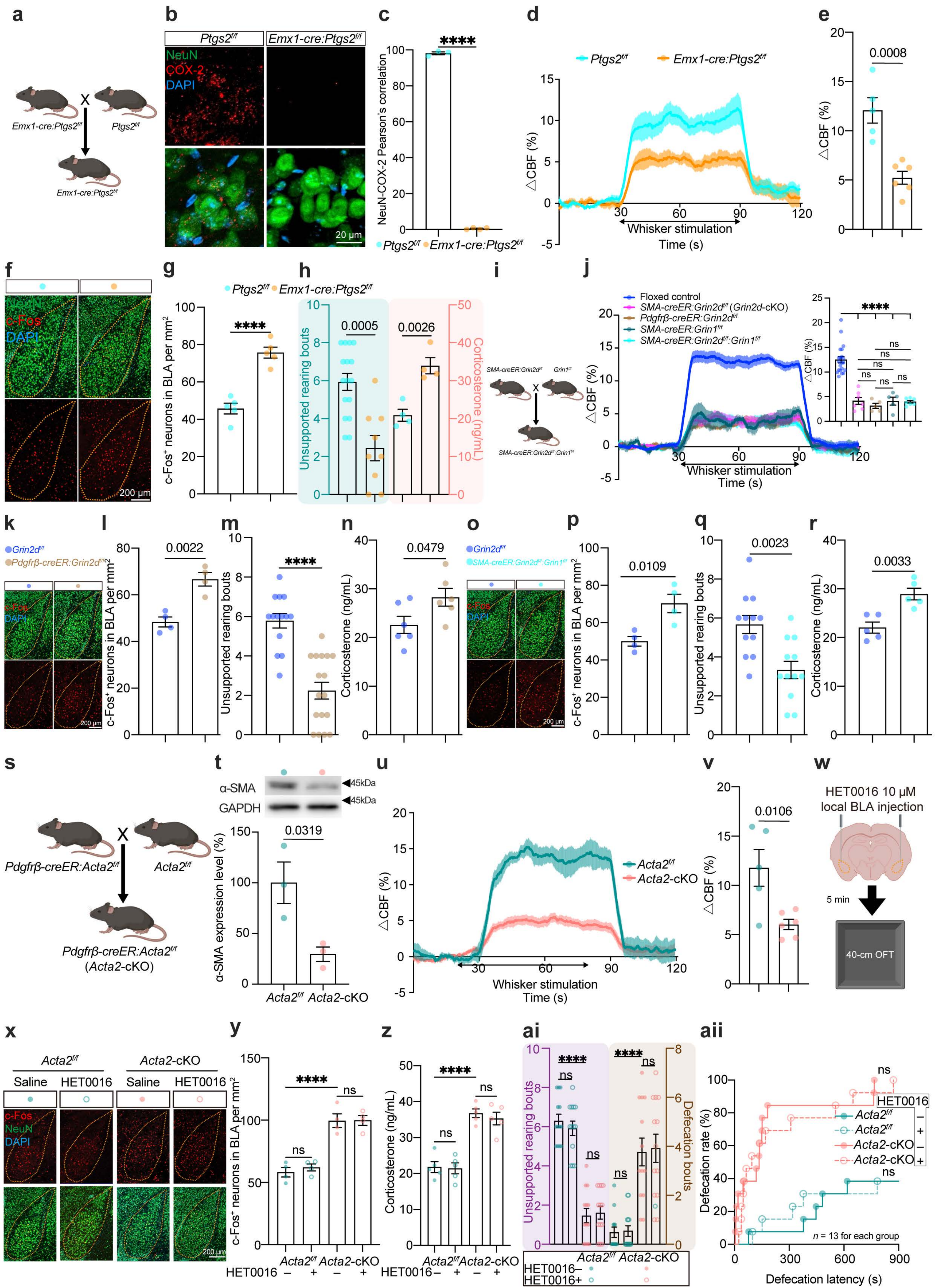

Fig.S12 NVC dysfunction across multiple models drives negative emotionality. a-h Validation of the *Emx1-cre:Ptgs2* model. Experimental schematic of mating strategy (**a**). Representative images (**b**) and quantification of neuronal COX-2 (encoded by *Ptgs2*) expression (**c**). Functional hyperemia in the S1BF (**d, e**), BLA neuronal activation (**f, g**), unsupported rearing bouts (**h, left**), and serum corticosterone (**h, right**) in the 40 cm OFT. **i-r** Validation of the mural cell-based NMDA conditional knock-out models. Mating strategy (**i**), functional hyperemia (**j**), BLA neuronal activation (**k, l**, and **o, p**). Unsupported rearing (**m, q**) and serum corticosterone (**n, r**) in the 40 cm OFT. **s-v** Validation of the *Acta2*-cKO model. Mating strategy (**s**), α-SMA immunoblot quantification (**t**), and functional hyperemia in the S1BF (**u, v**). **w-aii** Effects of local BLA HET0016 injection in *Acta2*-cKO mice. Experimental design (**w**), BLA neuronal activation (**x, y**), serum corticosterone concentrations (**z**), unsupported rearing bouts (**ai, left**), and defecation bouts (**ai, right**), and defecation rate and latency in the 40 cm OFT (**aii**) with local BLA HET0016 injection. Each dot represents one mouse. Shaded areas represent SEM across mice in panel (**d**, **j**, and **u**). Floxed control mice group in (**j**) including comparable 8 *Grin2d*$^{f/f}$ mice, 7 *Grin1*$^{f/f}$ mice, and 3 *Grin2d*$^{ff/}$*: Grin1*$^{f/f}$ mice. Control data (*Grin2d*$^{f/f}$, *Grin1*$^{f/f}$, *SMA-creER:Grin2d*$^{f/f}$, and *SMA-creER:Grin1*$^{f/f}$) are shared with **Fig. 2g**. Student's *t*-test (**c**, **e**, **g**, **h right**, **l**, **n**, **p**, **r**, **t**, and **v**). Mann-Whitney test (**h left**, **m**, and **q**). Two-way ANOVA followed by Tukey's multiple comparisons test for (**y,** P = 0.6564; **z**, P = 0.7144). Two-way ANOVA on rank-transformed data followed by

Sidak's multiple comparisons test (**ai left**, $P = 0.4442$; **ai right**, $P = 0.7932$). One-way ANOVA followed by Tukey's multiple comparisons test (**j**, $P < 0.0001$). Gehan-Breslow-Wilcoxon test (**aii**). Sample sizes (*n*) are indicated in the panel (**aii**). **** indicates $P < 0.0001$. Data are presented as mean ± SEM.
