# Supplementary material for: Neurovascular coupling in the basolateral amygdala modulates negative emotions
Source: Cell Res. 2026 May 27;36(9):656–79. doi: 10.1038/s41422-026-01256-2 (PMC13503848; doi:10.1038/s41422-026-01256-2)
Supplement: Supplementary file 6 [file 41422_2026_1256_MOESM15_ESM.pdf]

Figure S13

Ruan et al.

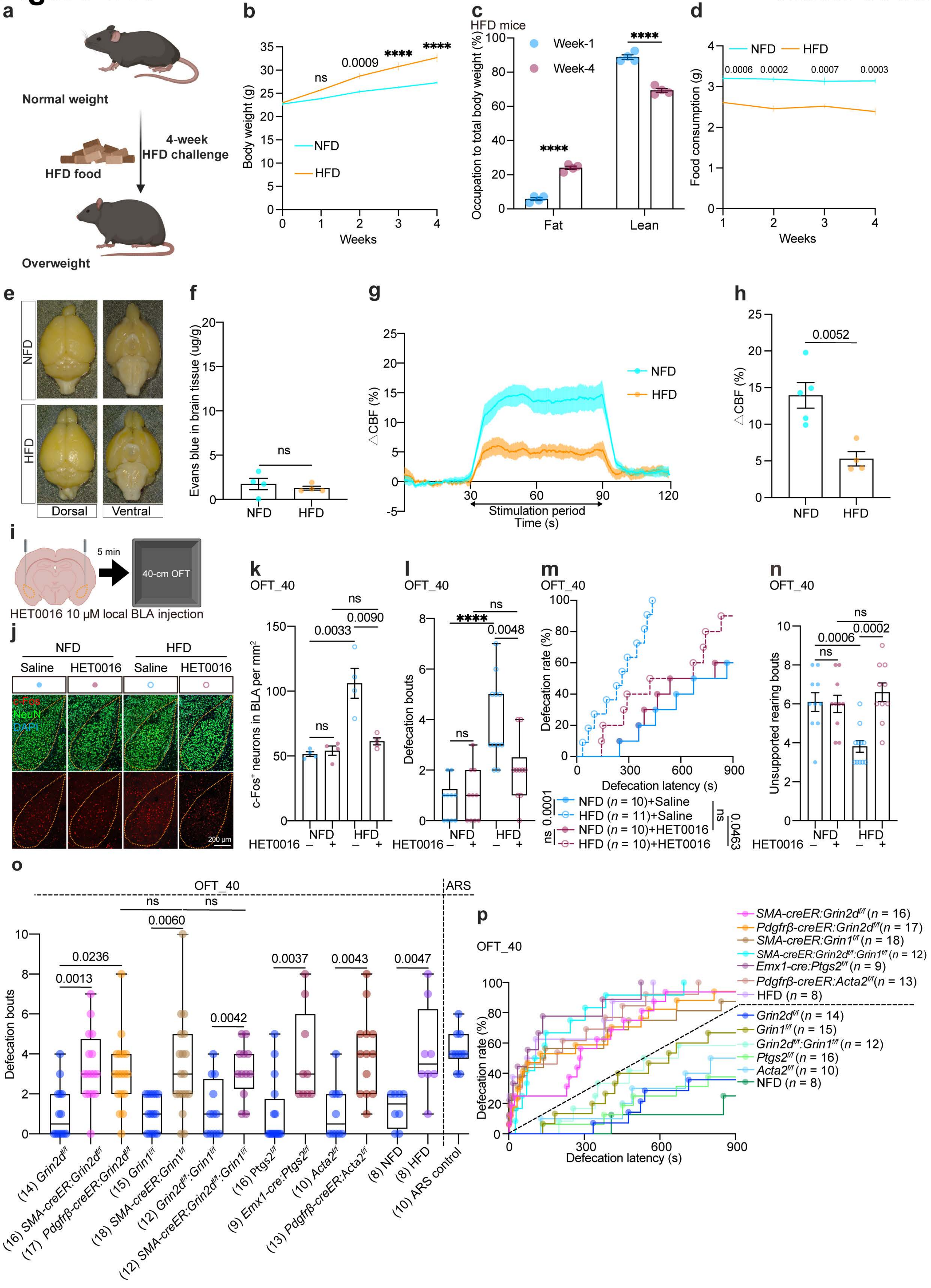

Fig.S13 HFD-induced NVC dysfunction and its impact on emotional distress. a-d High-fat diet (HFD) model validation. Schematic (**a**), body weight changes (**b**), body composition (fat and lean mass) changes (**c**), and food consumption (**d**) of mice with HFD or normal-fat diet (NFD). Student's *t*-test. n = 16 mice per group for **b** and **d**, two-way ANOVA followed by Tukey's multiple comparisons test ($P < 0.0001$). **e-f** Assessment of BBB integrity via Evans Blue injection (**e**) and quantification (**f**). Student's *t*-test. **g, h** Functional hyperemia in the S1BF (**g**) and quantification of the plateau phase (**h**). The shaded areas represent SEM across mice in (**g**). Student's *t*-test. **i-n** Effects of local BLA HET0016 injection in HFD mice. Experimental design (**i**), BLA neural activation (**j, k**), defecation bouts (**l**), defecation rate and latency (**m**), and unsupported rearing (**n**) of HFD mice in the 40 cm OFT with local BLA HET0016 injection. Two-way ANOVA followed by Tukey's multiple comparisons test for (**k**), P = 0.0001. Two-way ANOVA on rank-transformed data followed by Sidak's multiple comparisons test (**l** and **n**), $P < 0.0001$ for (**l**), and $P = 0.0009$ for (**n**). Gehan-Breslow-Wilcoxon test (**m**). Sample sizes (N) are indicated in the panel (**m**). **o, p** Defecation bouts (**o**), and defecation rate and latency (**p**) in the 40 cm OFT and ARS (panel **o** only). Kruskal-Wallis test followed by Dunn's multiple comparisons test for (**o**) (P < 0.0001). Gehan-Breslow-Wilcoxon test for (**p**). Sample sizes (N) are indicated in the panels (**o** and **p**). Control data (*Grin2d*$^{f/f}$and *SMA-creER:Grin2d*$^{f/ff}$) are shared with **Fig. 3a-c**. Detailed statistical data for panel (**p**) are shown in the Supplementary information, Table

S1, sub-table 6. Dotted line in (**p**) used to separate two clusters of distribution pattern between NVC-deficiency groups and their littermate controls. Each dot represents one mouse. **** indicates $P < 0.0001$. Data are presented as mean ± SEM.
