# Supplementary material for: Neurovascular coupling in the basolateral amygdala modulates negative emotions
Source: Cell Res. 2026 May 27;36(9):656–79. doi: 10.1038/s41422-026-01256-2 (PMC13503848; doi:10.1038/s41422-026-01256-2)
Supplement: Supplementary file 7 [file 41422_2026_1256_MOESM16_ESM.pdf]

Figure S14

Ruan et al.

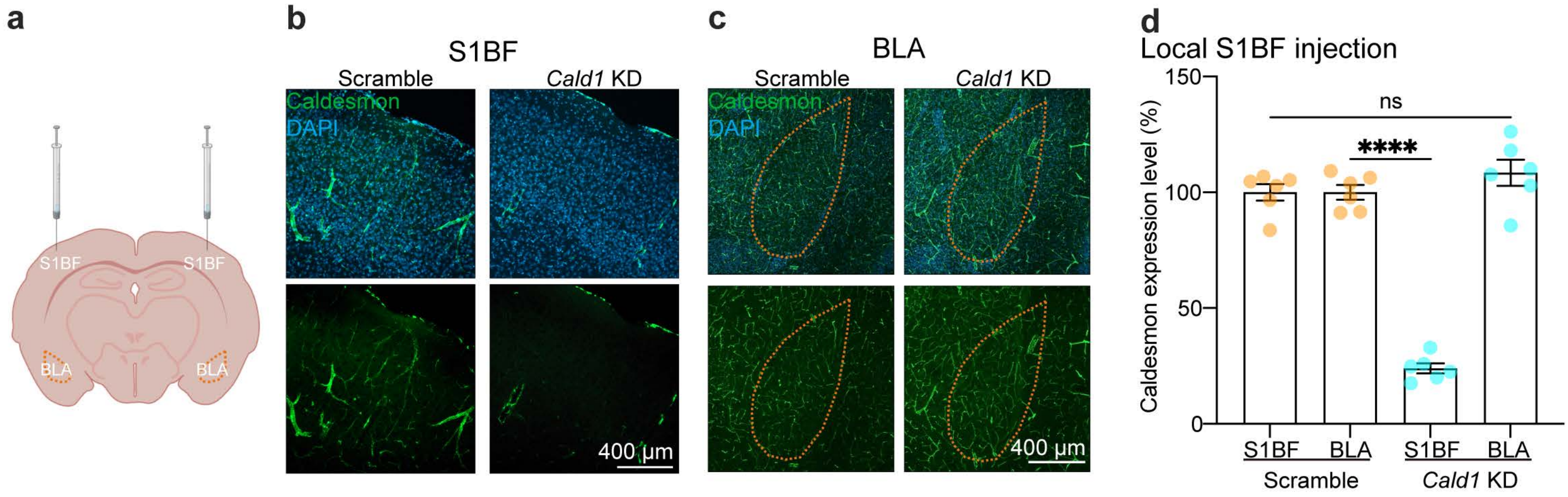

Fig.S14 Specificity of S1BF *Cald1* knockdown. a Schematic showing the siRNA injection site in the S1BF and its spatial relationship with the BLA (orange dotted line). **b-d** Representative images of caldesmon IF staining in the S1BF (**b**) and BLA (**c**) following local S1BF *Cald1* siRNA injection, with quantification in (**d**). Each dot represents one mouse. Two-way ANOVA followed by Tukey's multiple comparisons test ($P < 0.0001$). **** indicates $P < 0.0001$. Data are presented as mean ± SEM.
