# Supplementary material for: Neurovascular coupling in the basolateral amygdala modulates negative emotions
Source: Cell Res. 2026 May 27;36(9):656–79. doi: 10.1038/s41422-026-01256-2 (PMC13503848; doi:10.1038/s41422-026-01256-2)
Supplement: Supplementary file 8 [file 41422_2026_1256_MOESM17_ESM.pdf]

Figure S15

Ruan et al.

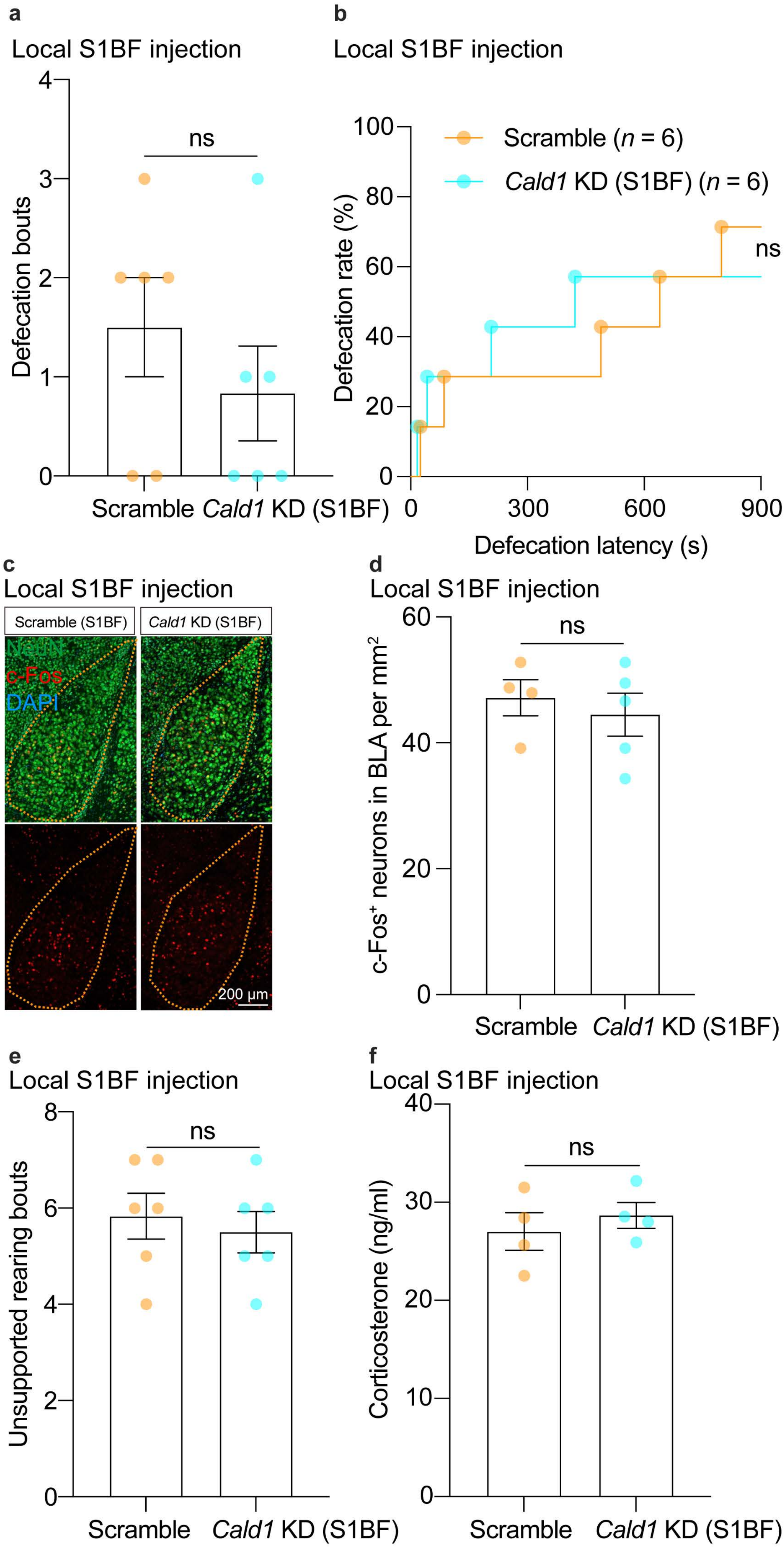

Fig.S15 Impact of S1BF *Cald1* knockdown on emotional responses. a, b Defecation bouts (**a**), and defecation rate and latency (**b**) in the 40 cm OFT following S1BF *Cald1* KD in the 40 cm OFT. Each dot represents one mouse. Mann-Whitney test in (**a**). Sample sizes (*n*) are indicated in the panel (**b**). Gehan-Breslow-Wilcoxon test in (**b**). **c, d** Representative images (**c**) and quantification (**d**) of BLA neuronal activation in the 40 cm OFT. Each dot represents one mouse. Student's *t*-test. **e, f** Unsupported rearing bouts (**e**) and serum corticosterone concentrations (**f**) in the 40 cm OFT. Each dot represents one mouse. Mann-Whitney test in (**e**), and Student's *t*-test in (**f**). Data are presented as mean ± SEM.
