# Supplementary material for: Neurovascular coupling in the basolateral amygdala modulates negative emotions
Source: Cell Res. 2026 May 27;36(9):656–79. doi: 10.1038/s41422-026-01256-2 (PMC13503848; doi:10.1038/s41422-026-01256-2)
Supplement: Supplementary file 9 [file 41422_2026_1256_MOESM18_ESM.pdf]

Figure S16

Ruan et al.

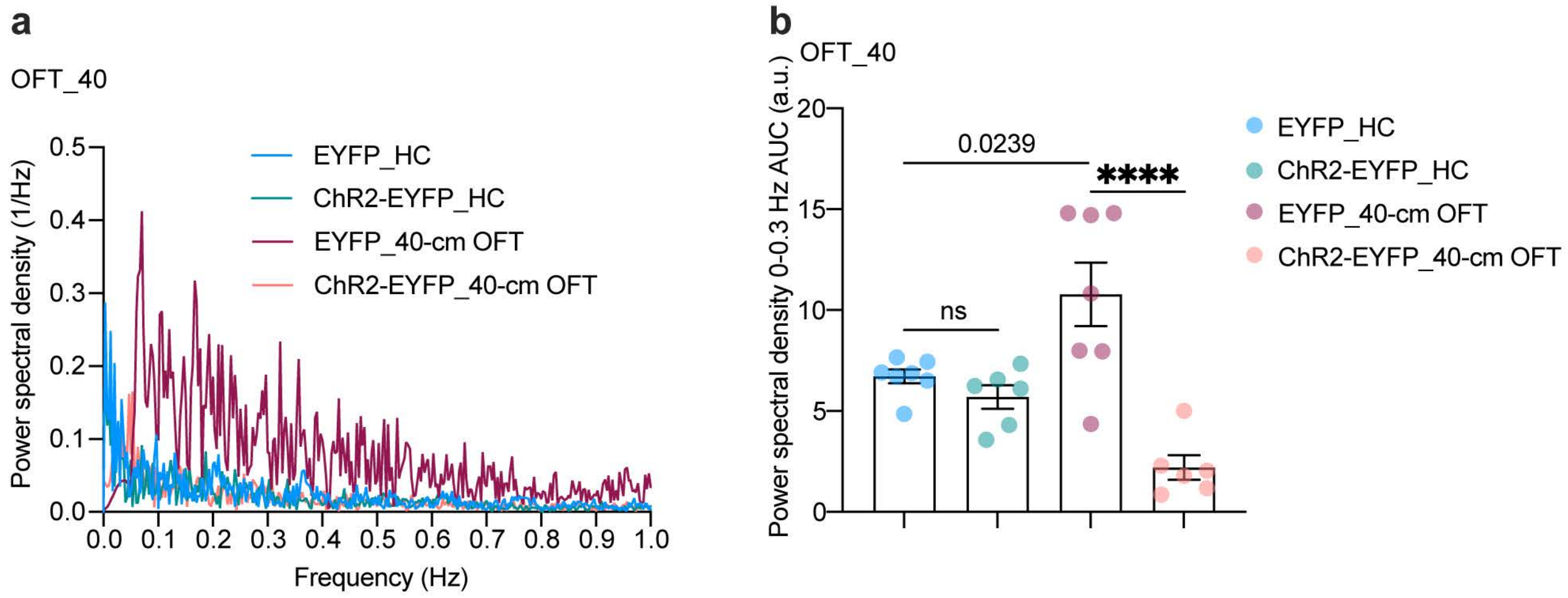

Fig.S16 Spectral energy analysis of BLA blood flow signals. a, b Representative Fourier plot of BLA CBV signals from 0-1 Hz (**a**) and quantification of ultra-low-frequency oscillations (0-0.3 Hz) (**b**) in the 40 cm OFT. Each dot represents one mouse. One-way ANOVA followed by Tukey's multiple comparisons test ($P < 0.0001$). **** indicates $P < 0.0001$. Data are presented as mean ± SEM.
