# Supplementary material for: Neurovascular coupling in the basolateral amygdala modulates negative emotions
Source: Cell Res. 2026 May 27;36(9):656–79. doi: 10.1038/s41422-026-01256-2 (PMC13503848; doi:10.1038/s41422-026-01256-2)
Supplement: Supplementary file 10 [file 41422_2026_1256_MOESM19_ESM.pdf]

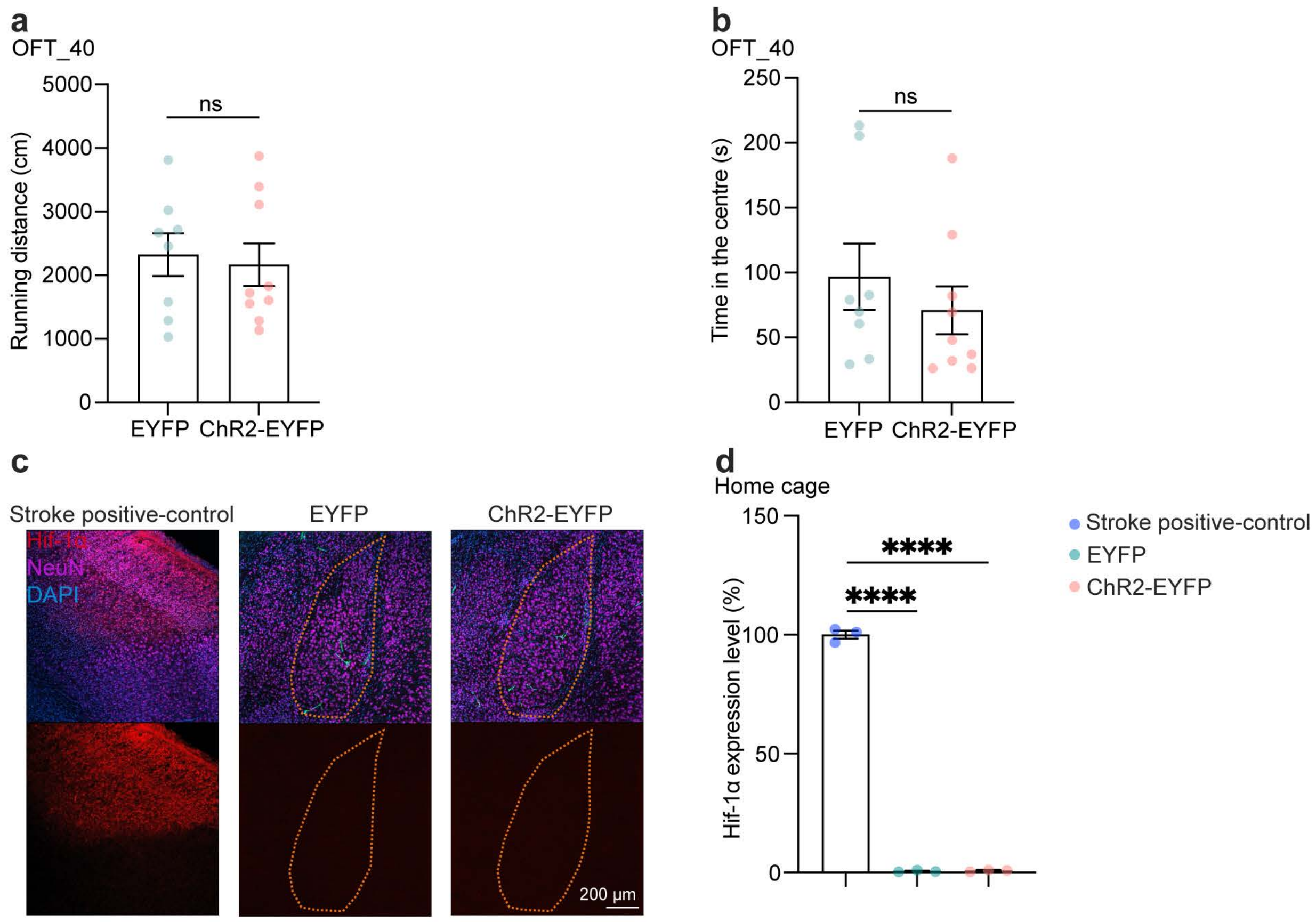
a
OFT_40
Running distance (cm)
5000
4000
3000
2000
1000
0
ns
EYFP
ChR2-EYFP
b
OFT_40
Time in the centre (s)
250
200
150
100
50
0
ns
EYFP
ChR2-EYFP
c
Stroke positive-control
EYFP
ChR2-EYFP
Hif-1α
NeuN
DAPI
200 μm
d
Home cage
Hif-1α expression level (%)
150
100
50
0
****
****
Stroke positive-control
EYFP
ChR2-EYFP

**Fig.S17 Supplementary data for BLA optogenetic stimulation. a, b** Running distance (**a**) and time spent in the center zone (**b**) during optogenetic stimulation in the 40 cm OFT. Each dot represents one mouse. Student's *t*-test. **c, d** Representative images (**c**) and quantification (**d**) of Hif-1α immunofluorescence in the BLA during the optogenetic stimulation in the home cage. Stroke model tissue serves as a positive control. Each dot represents one mouse. One-way ANOVA followed by Tukey's multiple comparisons test ($P < 0.0001$). **** indicates $P < 0.0001$. Data are presented as mean ± SEM.
