# Supplementary material for: Neurovascular coupling in the basolateral amygdala modulates negative emotions
Source: Cell Res. 2026 May 27;36(9):656–79. doi: 10.1038/s41422-026-01256-2 (PMC13503848; doi:10.1038/s41422-026-01256-2)
Supplement: Supplementary file 11 [file 41422_2026_1256_MOESM20_ESM.pdf]

Figure S18

Ruan et al.

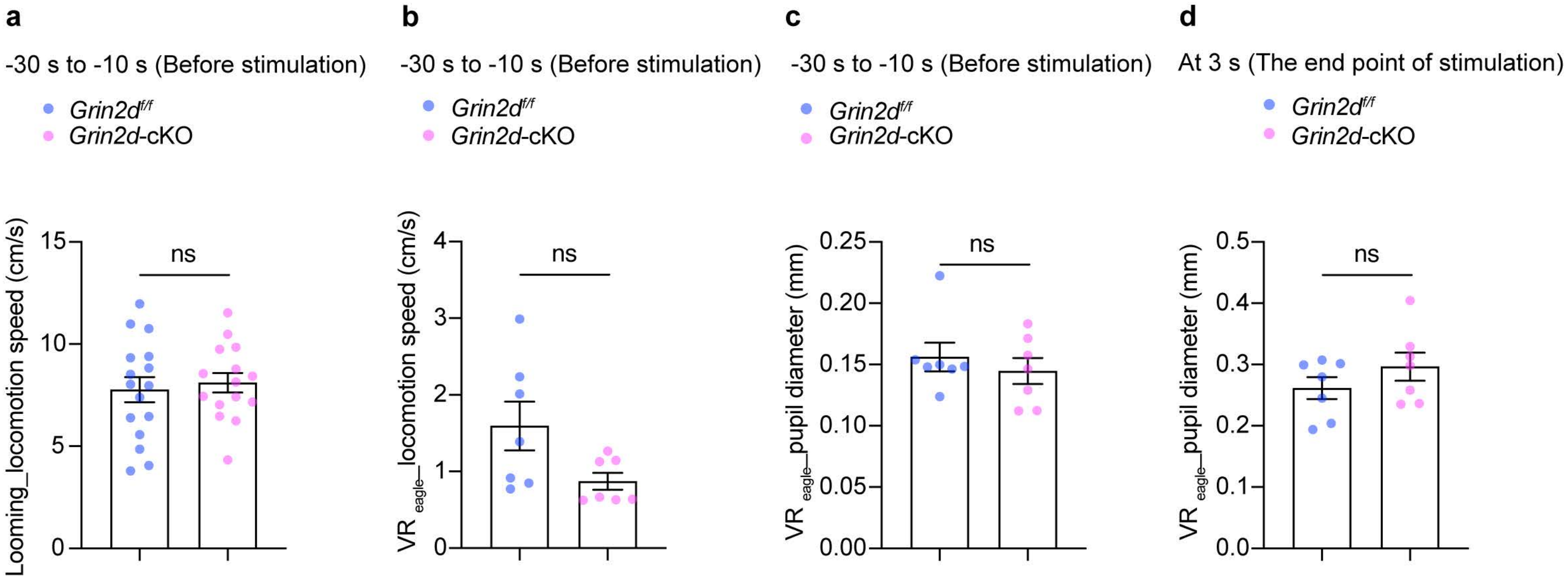

Fig.S18 Supplementary locomotion speed and pupil diameter in looming and VR_{eagle} assays. a-d Quantification of pre-stimulation locomotion speed in looming (**a**) and VR_{eagle} (**b**) assays, and pupil diameter before (**c**) and at the conclusion (**d**) of VR_{eagle} stimulation. Each dot represents one mouse. Student's *t*-test. Data are presented as mean ± SEM.
