# Supplementary material for: Neurovascular coupling in the basolateral amygdala modulates negative emotions
Source: Cell Res. 2026 May 27;36(9):656–79. doi: 10.1038/s41422-026-01256-2 (PMC13503848; doi:10.1038/s41422-026-01256-2)
Supplement: Supplementary file 12 [file 41422_2026_1256_MOESM21_ESM.pdf]

Figure S19

Ruan et al.

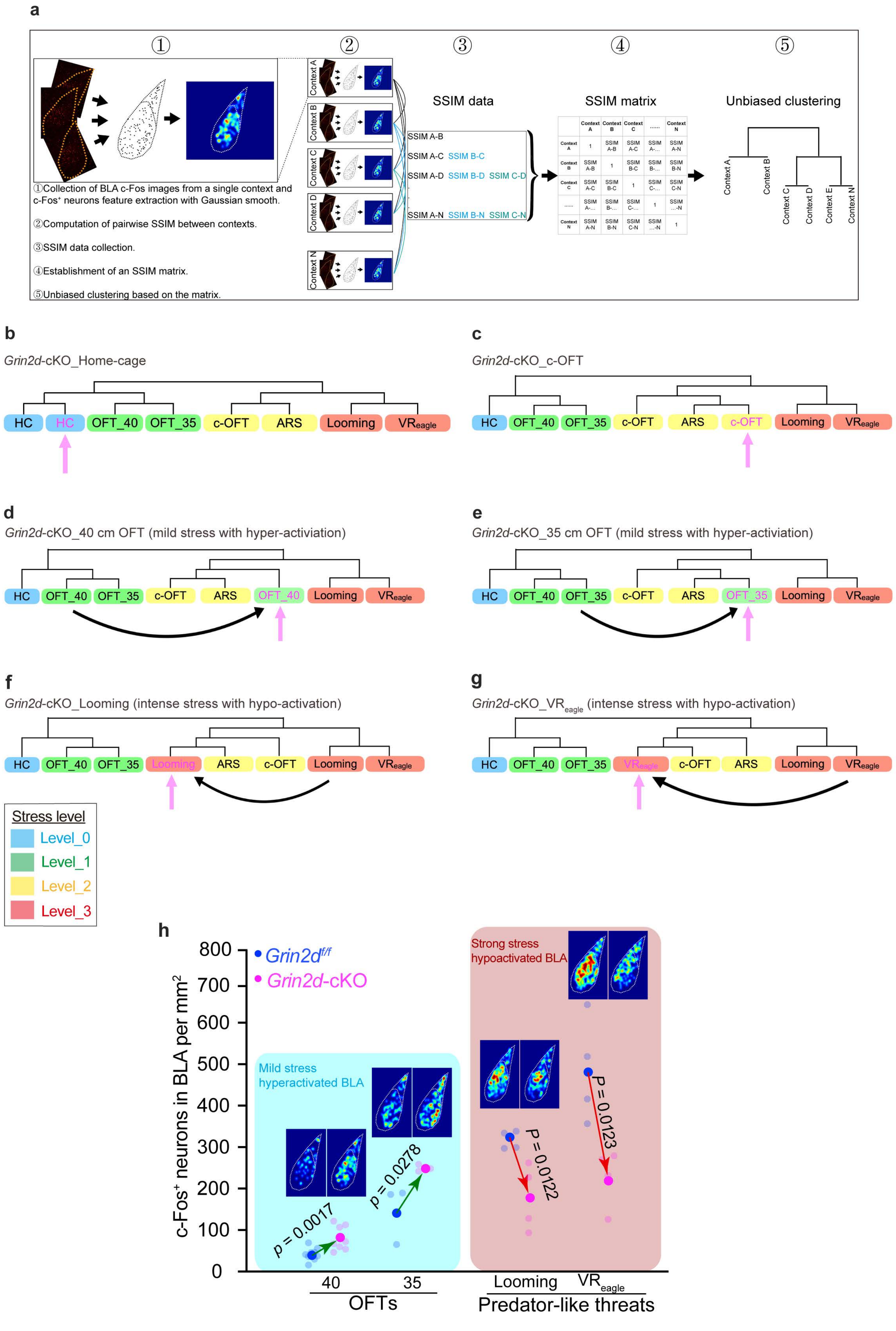

Fig.S19 Methodological framework for topological clustering and clustering coordinates of *Grin2d*-cKO mice. a Schematic workflow for clustering data processing and generation. **b-g**, Clustering coordinates of *Grin2d*-cKO mice mapped against the unbiased classification library of littermate controls across individual contexts. **h** The summary of the load-dependent biphasic pattern. Quantification data shared with **Fig. 6j**, and heatmap shared with **Fig. 6l, m**. Each 50% transparent dot represents one mouse. The non-transparent dot represents the mean value. Student's *t*-test. Data are presented as mean ± SEM.
