# Supplementary material for: Neurovascular coupling in the basolateral amygdala modulates negative emotions
Source: Cell Res. 2026 May 27;36(9):656–79. doi: 10.1038/s41422-026-01256-2 (PMC13503848; doi:10.1038/s41422-026-01256-2)
Supplement: Supplementary file 13 [file 41422_2026_1256_MOESM22_ESM.pdf]

Figure S20

Ruan et al.

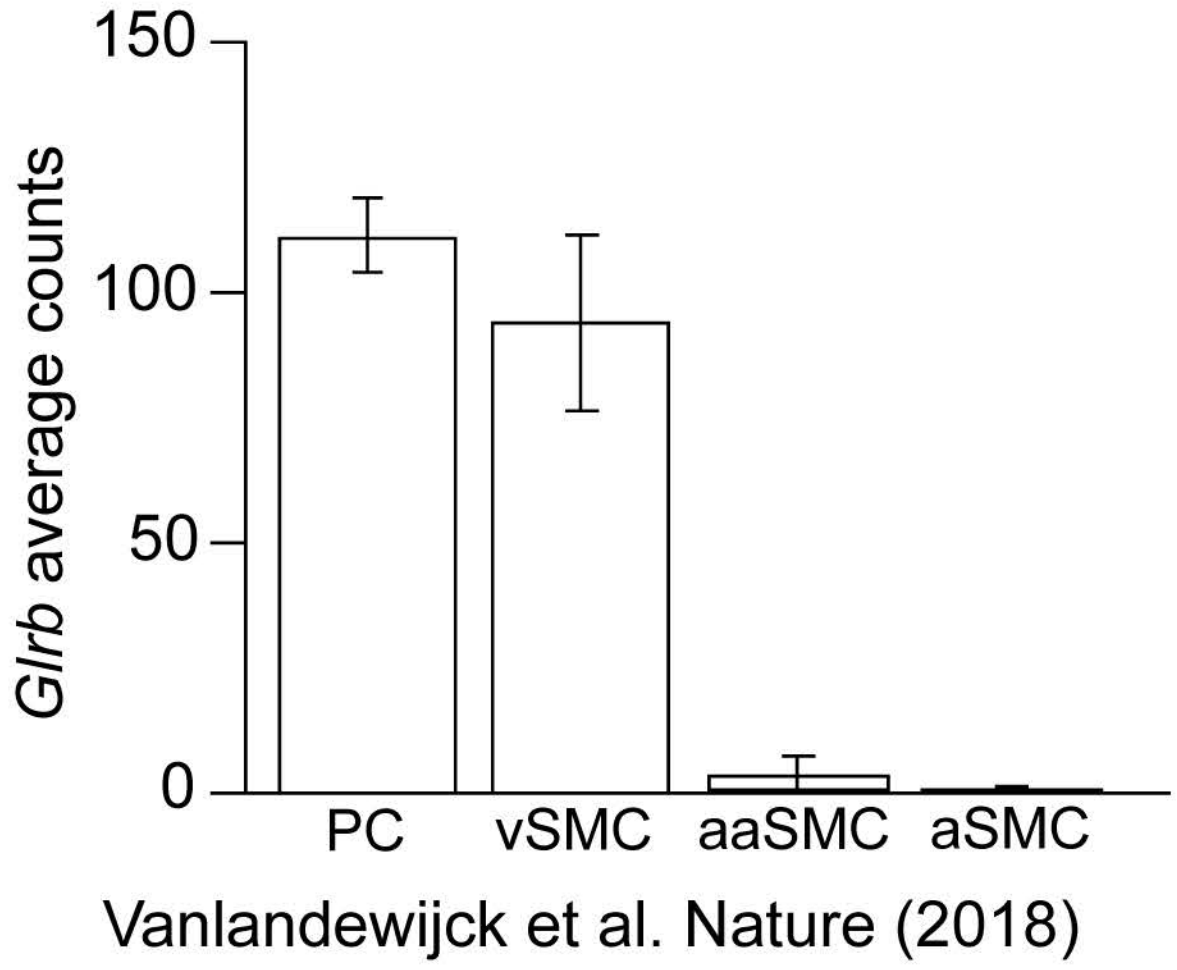

Fig.S20 *Glrb* expression profile in the vascular transcriptome database. Transcriptional levels of *Glrb* in pericytes (PC), venous SMCs (vSMCs), arteriolar SMCs (aaSMCs), and arterial SMCs (aSMCs). Data are presented as mean ± SEM.
