# Supplementary material for: Neurovascular coupling in the basolateral amygdala modulates negative emotions
Source: Cell Res. 2026 May 27;36(9):656–79. doi: 10.1038/s41422-026-01256-2 (PMC13503848; doi:10.1038/s41422-026-01256-2)
Supplement: Supplementary file 14 [file 41422_2026_1256_MOESM23_ESM.pdf]

Figure S21

Ruan et al.

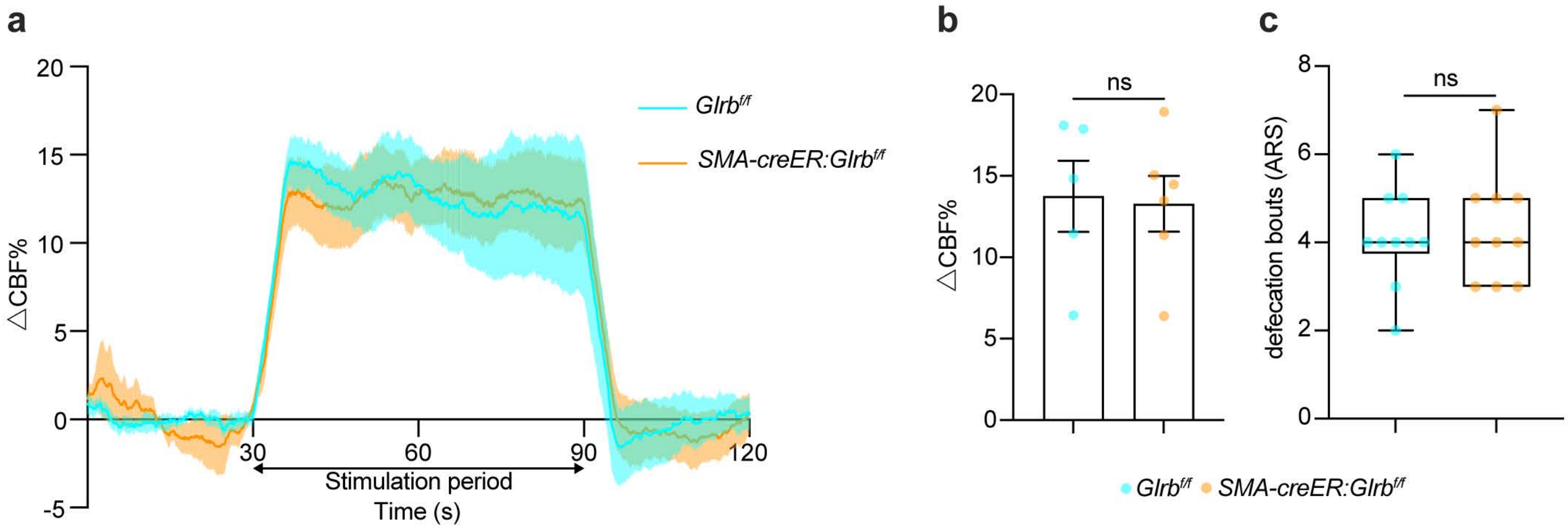

Fig.S21 Functional and behavioral validation of SMC-specific *Glrb*-cKO mice. **a, b** Functional hyperemia in the S1BF (**a**) and quantification of the plateau phase (**b**). **c** Defecation bouts of mice during the ARS. Each dot represents one mouse. Shaded areas represent SEM across mice in (**a**). Student's *t*-test (**b**) and Mann-Whitney test (**c**). Data are presented as mean ± SEM.
