# Supplementary material for: Neurovascular coupling in the basolateral amygdala modulates negative emotions
Source: Cell Res. 2026 May 27;36(9):656–79. doi: 10.1038/s41422-026-01256-2 (PMC13503848; doi:10.1038/s41422-026-01256-2)
Supplement: Supplementary file 15 [file 41422_2026_1256_MOESM24_ESM.pdf]

Figure S22

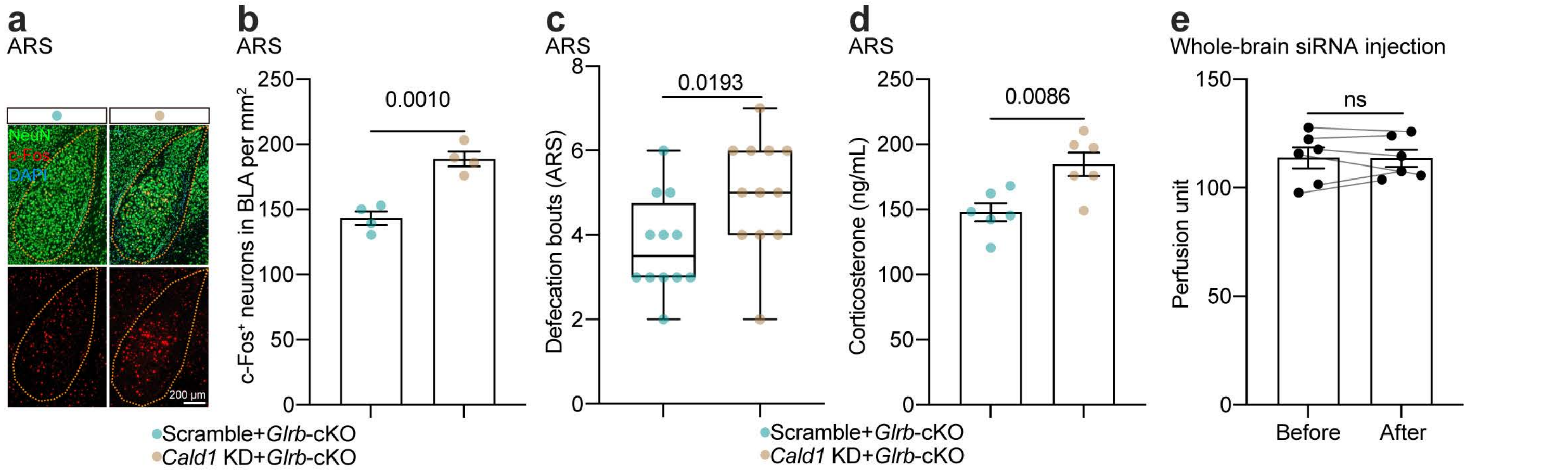
a
ARS
NeuN
c-Fos
DAPI
200 μm
b
ARS
c-Fos+ neurons in BLA per mm2
250
200
150
100
50
0
0.0010
Scramble+Glrb-cKO
Cald1 KD+Glrb-cKO
c
ARS
Defecation bouts (ARS)
8
6
4
2
0
0.0193
d
ARS
Corticosterone (ng/mL)
250
200
150
100
50
0
0.0086
Scramble+Glrb-cKO
Cald1 KD+Glrb-cKO
e
Whole-brain siRNA injection
Perfusion unit
150
100
50
0
ns
Before
After

Fig.S22 Local BLA *Cald1* knockdown occludes the NVC enhancement in *Glrb*-cKO mice. a, b Representative images (**a**) and quantification (**b**) of BLA neuronal activation of *Glrb*-cKO mice with local BLA *Cald1* KD in ARS. Student's *t*-test. **c, d** Defecation bouts (**c**) and serum corticosterone (**d**) in *Glrb*-cKO mice with local BLA *Cald1* KD in ARS. Mann-Whitney test (**c**) and Student's *t*-test (**d**). **e** Baseline cerebral perfusion analysis before and after *Cald1* siRNA cisterna magna injection in the *Glrb*-cKO mice. Paired Student's *t*-test. Each dot represents one mouse. Data are presented as mean ± SEM.
