# Supplementary material for: Neurovascular coupling in the basolateral amygdala modulates negative emotions
Source: Cell Res. 2026 May 27;36(9):656–79. doi: 10.1038/s41422-026-01256-2 (PMC13503848; doi:10.1038/s41422-026-01256-2)
Supplement: Supplementary file 16 [file 41422_2026_1256_MOESM25_ESM.pdf]

Figure S23

Ruan et al.

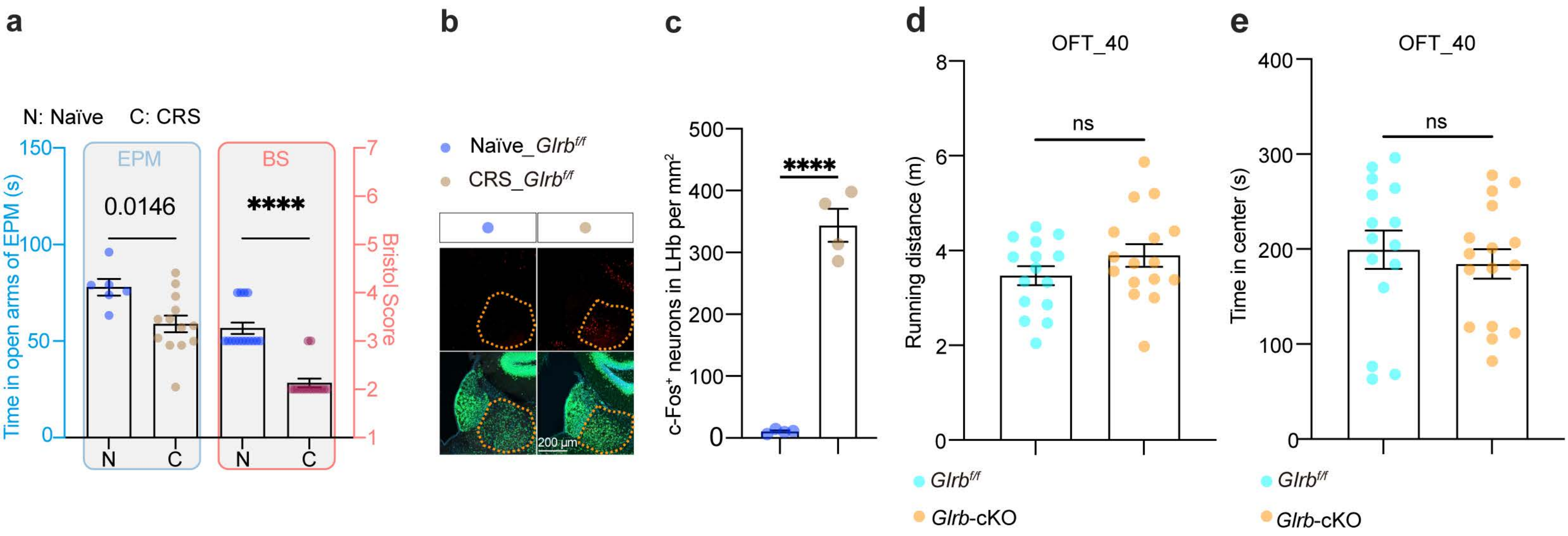

Fig.S23 CRS modeling and *Glrb*-cKO mice naïve behaviors in the 40-cm OFT. **a** Time spent in the open arms of the elevated plus maze after CRS modeling (EPM, left) and fecal quality (Bristol score, right, data similar to that in **Fig. 8m**) at day-28 of CRS. Student's *t*-test (left) and Mann-Whitney test (right). **b, c** Representative images (**b**) and quantification (**c**) of neuronal activation in the lateral habenula (LHb) after CRS modeling. Student's *t*-test. **d, e** Total running distance (**d**) and time spent in the center zone (**e**) in the 40 cm OFT. Naïve indicates mice without CRS modeling as controls. Each dot represents one mouse. Student's *t*-test. **** indicates $P < 0.0001$. Data are presented as mean ± SEM.
