# Supplementary material for: Neurovascular coupling in the basolateral amygdala modulates negative emotions
Source: Cell Res. 2026 May 27;36(9):656–79. doi: 10.1038/s41422-026-01256-2 (PMC13503848; doi:10.1038/s41422-026-01256-2)
Supplement: Supplementary file 17 [file 41422_2026_1256_MOESM26_ESM.pdf]

Figure S24

Ruan et al.

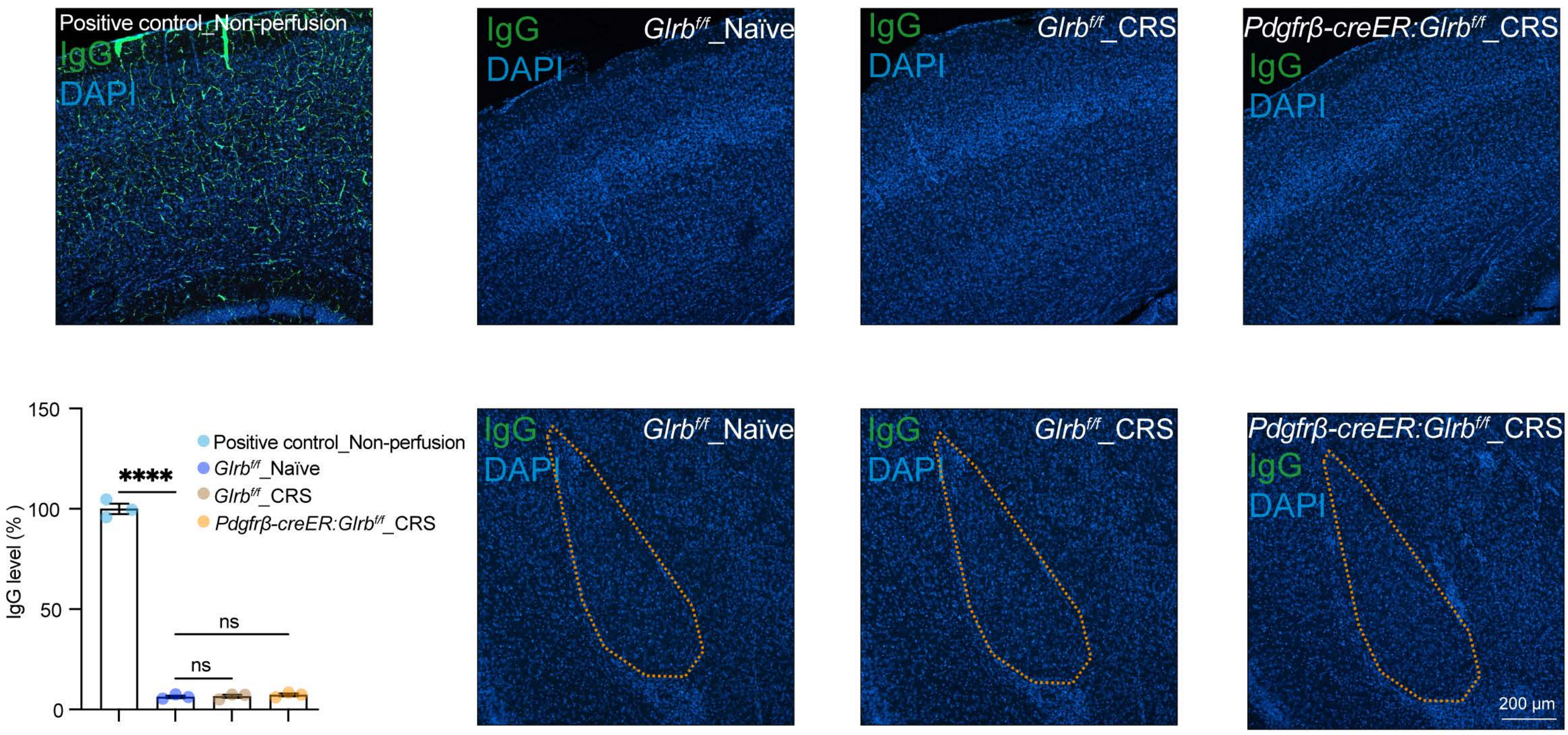

Fig.S24 Assessment of BBB integrity following CRS. Representative images (including non-perfusion positive control) and quantification of IgG fluorescence in the S1BF and BLA after CRS modeling. Each dot represents the average IgG level per mouse, calculated from three ROIs in both the cortex and the BLA. One-way ANOVA followed by Tukey’s multiple comparisons test ($P < 0.0001$). **** indicates $P < 0.0001$. Data are presented as mean ± SEM.
