# Supplementary material for: Neurovascular coupling in the basolateral amygdala modulates negative emotions
Source: Cell Res. 2026 May 27;36(9):656–79. doi: 10.1038/s41422-026-01256-2 (PMC13503848; doi:10.1038/s41422-026-01256-2)
Supplement: Supplementary file 18 [file 41422_2026_1256_MOESM27_ESM.pdf]

Figure S25

Ruan et al.

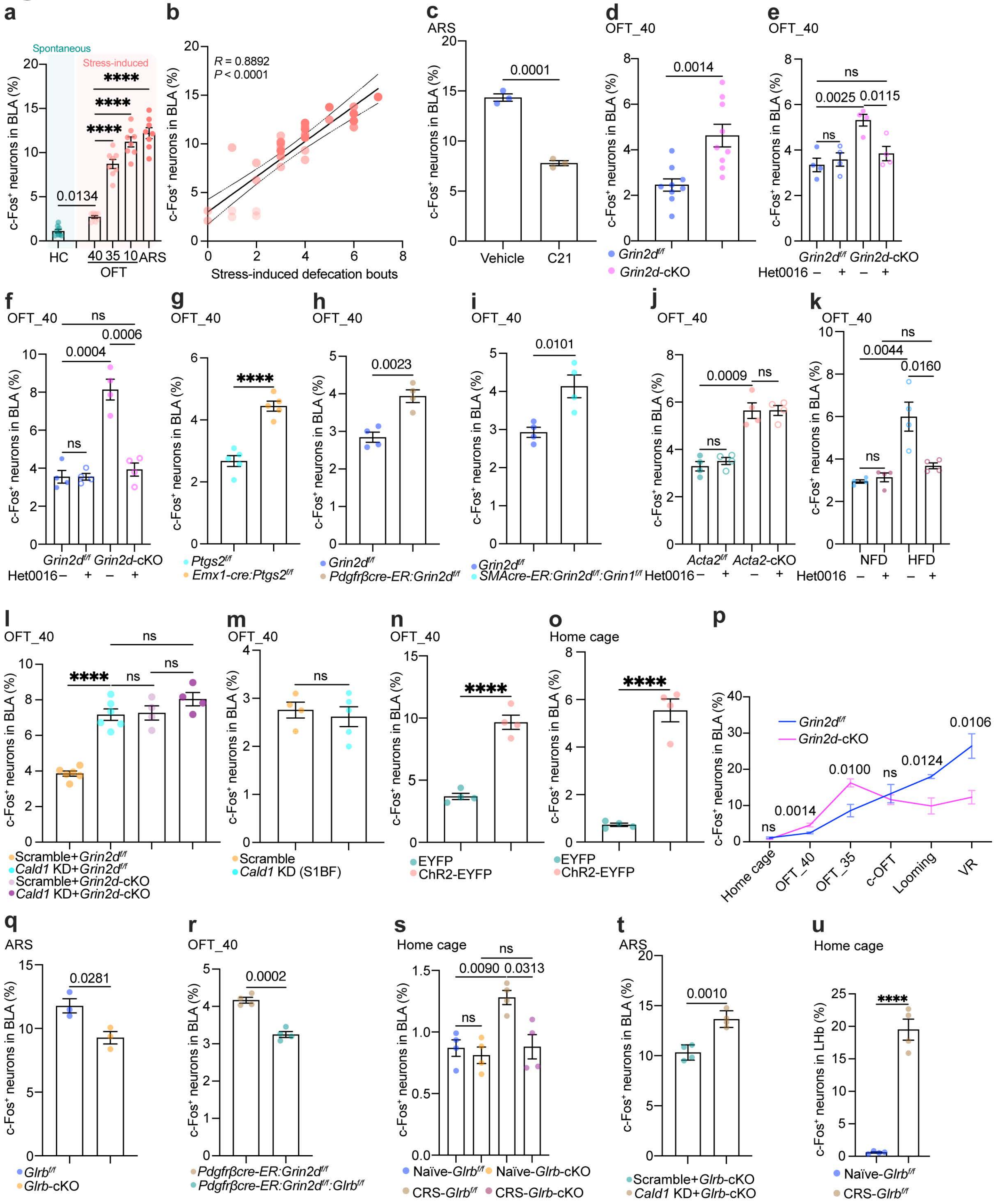

Fig.S25 Statistical quantification of the percentage of c-Fos positive neurons in the BLA. a-u Re-quantification of c-Fos positive neuron ratios (normalized to total NeuN positive neurons in the BLA) corresponding to the original intensity-based data presented in Fig. 1c (**a**), Fig. 1e (**b**), Fig. 1o (**c**), Fig. 2m (**d**), Fig. 3g (**e**), Fig. 3m (**f**), Fig. S12g (**g**), Fig. S12l (**h**), Fig. S12p (**i**), Fig. S12y (**j**), Fig. S13k (**k**), Fig. 4m (**l**), Fig. S15d (**m**), Fig. 5i (**n**), Fig. 5r left (**o**), Fig. 6j (**p**), Fig. 8b (**q**), Fig. 8h (**r**), Fig. 8p left (**s**), Fig. S22b (**t**), and Fig. S23c (**u**), respectively.
