# Supplementary material for: Neurovascular coupling in the basolateral amygdala modulates negative emotions
Source: Cell Res. 2026 May 27;36(9):656–79. doi: 10.1038/s41422-026-01256-2 (PMC13503848; doi:10.1038/s41422-026-01256-2)
Supplement: Supplementary file 19 [file 41422_2026_1256_MOESM28_ESM.pdf]

Supplementary Table S1. P_{value} of Defecation rate-latency curve

Sub table 1 for **Fig. 3c**

P	1	2	3	4	5	6
1	1	0.0031	0.0019	****	****	****
2		1	0.1934	0.3581	0.1384	0.1189
3			1	0.8685	0.3193	0.3458
4				1	0.1585	0.1083
5					1	0.9278
6						1

1. OFT_40 *Grin2d*$^{f/f}$
2. OFT_35 *Grin2d*$^{f/f}$
3. OFT_10 *Grin2d*$^{f/f}$
4. OFT_40 *Grin2d*-cKO
5. OFT_40 *Grin2d*-cKO
6. OFT_40 *Grin2d*-cKO

**** indicates $P < 0.0001$

Sub table 2 for **Fig. 3i**

P	1	2	3	4
1	1	0.1650	****	0.6470
2		1	****	0.2934
3			1	****
4				1

1. *Grin2d*$^{f/f}$+Saline
2. *Grin2d*$^{f/f}$+HET0016
3. *Grin2d*-cKO+Saline
4. *Grin2d*-cKO+HET0016

**** indicates $P < 0.0001$

Sub table 3 for **Fig. 3o**

P	1	2	3	4
1	1	0.0679	0.0003	0.2123
2		1	****	0.3173
3			1	****
4				1

5. *Grin2d*$^{f/f}$+Saline

6. *Grin2d*$^{f/f}$+HET0016

7. *Grin2d*-cKO+Saline

8. *Grin2d*-cKO+HET0016

**** indicates $P < 0.0001$

Sub table 4 for **Fig. 4q**

P	1	2	3	4	5
1	1	****	****	0.0009	****
2		1	0.5745	0.8881	0.9999
3			1	0.99990	0.6926
4				1	0.6353
5					1

1. Scramble (Whole-brain+BLA)

2. *Cald1* KD (Whole-brain)

3. *Cald1* KD (BLA)

4. Scramble (BLA)+*Grin2d*-cKO

5. *Cald1* KD (BLA)+*Grin2d*-cKO

**** indicates $P < 0.0001$

Sub table 5 for **Fig. 8f**

P	1	2	3	4
1	1	0.0012	0.6285	0.4703
2		1	0.0035	0.0244
3			1	0.2637
4				1

1. Floxed control mice
2. *Pdgfrβ-creER:Grin2d*$^{f/f}$
3. *Pdgfrβ-creER:Glrb*$^{f/f}$
4. *Pdgfrβ-creER:Grin2d*$^{f/f}$*:Glrb*$^{f/f}$

**** indicates $P < 0.0001$

Sub table 6 for **Fig. S13p**

P	1	2	3	4	5	6	7	8	9	10	11	12
1	1	****	0.0002	0.0823	0.0005	0.8480	****	****	0.4906	****	0.5680	****
2		1	0.7333	0.0092	0.7209	0.0002	0.0786	0.2073	0.0043	0.4279	0.0010	0.2679
3			1	0.0047	0.7747	0.0001	0.2580	0.3542	0.0028	0.7549	0.0010	0.4218

4				1	0.0130	0.1038	****	0.0001	0.2961	0.0009	0.0548	0.0005
5					1	0.0004	0.2596	0.3170	0.0078	0.9480	0.0022	0.5377
6						1	****	****	0.5970	****	0.4793	****
7							1	0.4699	****	0.2935	0.0003	0.8118
8								1				
9								****	1	0.0008	0.2695	0.0005
10								0.3463		1	0.0003	0.5583
11								****			1	0.0005
12								0.9373				1

1. *Grin2d*$^{f/f}$
2. *SMA-creER:Grin2d*$^{f/f}$
3. *Pdgfrβ-creER:Grin2d*$^{f/f}$
4. *Grin1*$^{f/f}$
5. *SMA-creER:Grin1*$^{f/f}$
6. *Ptgs2*$^{f/f}$
7. *Emx1-cre:Ptgs2*$^{f/f}$
8. *SMA-creER:Grin1*$^{f/f}$*:Grin2d*$^{f/f}$
9. *Acta2*$^{f/f}$
10. *Pdgfrβ-creER:Acta2*$^{f/f}$
11. NFD

12. HFD

**** indicates $P < 0.0001$
