# Supplementary material for: Neurovascular coupling in the basolateral amygdala modulates negative emotions
Source: Cell Res. 2026 May 27;36(9):656–79. doi: 10.1038/s41422-026-01256-2 (PMC13503848; doi:10.1038/s41422-026-01256-2)
Supplement: Supplementary file 20 [file 41422_2026_1256_MOESM29_ESM.pdf]

Supplementary Table S2. Sample source checklist

No.	Location	Sample type	Source
1	Fig. 1b	Immunofluorescence staining	Brain slice
2	Fig. 1n	Immunofluorescence staining	Brain slice
3	Fig. 2b	Semi-quantified PCR	Sorted cultured cell
4	Fig. 2c	RNAscope	Brain slice
5	Fig. 2d	Immunofluorescence staining	Isolated MCA
6	Fig. 2l	Immunofluorescence staining	Brain slice
7	Fig. S1a	RNAscope	Brain slice
8	Fig. S1c	Western blot	Cerebellum lysates
9	Fig. S1d	Western blot	Sorted cultured cell
10	Fig. S1e	Western blot	Cortex lysates
11	Fig. S2a	Immunofluorescence staining	Tissue slice
12	Fig. S3i	Hematoxylin and eosin staining	Intestine slice
13	Fig. S3o	Absorbance measurement	Brain lysates
14	Fig. S4a	Western blot	Cortex lysates
15	Fig. S4b	Immunofluorescence staining	Isolated MCA

16	Fig. 3f	Immunofluorescence staining	Brain slice
17	Fig. 3l	Immunofluorescence staining	Brain slice
18	Fig. S12b	Immunofluorescence staining	Brain slice
19	Fig. S12f	Immunofluorescence staining	Brain slice
20	Fig. S12k	Immunofluorescence staining	Brain slice
21	Fig. S12o	Immunofluorescence staining	Brain slice
22	Fig. S12t	Western blot	Cortex lysates
23	Fig. S12x	Immunofluorescence staining	Brain slice
24	Fig. S13e	Absorbance measurement	Brain lysates
25	Fig. S13j	Immunofluorescence staining	Brain slice
26	Fig. 4b	Western blot	Sorted culture cell
27	Fig. 4d	Immunofluorescence staining	Brain slice
28	Fig. 4f	Semi-quantified PCR	Cortex lysate
29	Fig. 4g	Immunofluorescence staining	Brain slice
30	Fig. 4h	Immunofluorescence staining	Brain slice
31	Fig. 4l	Immunofluorescence staining	Brain slice
32	Fig. S14b	Immunofluorescence staining	Brain slice

33	Fig. S14c	Immunofluorescence staining	Brain slice
34	Fig. S15c	Immunofluorescence staining	Brain slice
35	Fig. 5a	Immunofluorescence staining	Brain slice
36	Fig. 5h	Immunofluorescence staining	Brain slice
37	Fig. 5q	Immunofluorescence staining	Brain slice
38	Fig. S17c	Immunofluorescence staining	Brain slice
39	Fig. 7a	RNAscope	Brain slice
40	Fig. 7c	Immunofluorescence staining	Brain slice
41	Fig. 7f	Semi-quantified PCR	Acute sorted cell
42	Fig. 7g	Immunofluorescence staining	Cultured cell line
43	Fig. 7h	Immunofluorescence staining	Brain slice
44	Fig. 7i	Electron microscopy image	Brain slice
45	Fig. 8a	Immunofluorescence staining	Brain slice
46	Fig. 8g	Immunofluorescence staining	Brain slice
47	Fig. 8o	Immunofluorescence staining	Brain slice
48	Fig. S22a	Immunofluorescence staining	Brain slice
49	Fig. S23b	Immunofluorescence staining	Brain slice

50	Fig. S24	Immunofluorescence staining	Brain slice
