# Supplementary material for: Neurovascular coupling in the basolateral amygdala modulates negative emotions
Source: Cell Res. 2026 May 27;36(9):656–79. doi: 10.1038/s41422-026-01256-2 (PMC13503848; doi:10.1038/s41422-026-01256-2)
Supplement: Supplementary file 21 [file 41422_2026_1256_MOESM30_ESM.pdf]

Supplementary Table S3. Mice information

Abbreviation	Full name	Source
Ai32	*B6;129S-Gt(ROSA)26Sortm32(CAG-COP4*H134R/EYFP)Hze/J*	*Hailan Hu, Zhejiang University*
Nestin-cre	*B6.Cg-Tg(Nes-cre)1Kln/J*	*Yanmei Tao and Xu Hu, Hangzhou Normal University/ Southeast University*
Emx1-cre	*B6.129S2-Emx1tm1(cre)Krj/J*	*Yanmei Tao and Xu Hu, Hangzhou Normal University/ Southeast University*
SMA-creER	*Tg(Acta2-cre/ERT2)12Pcn*	*Bo Zhou, ShanghaiInstitutes for Biological Sciences, Chinese Academy of Sciences*
Pdgfrβ-creER	*Tg(Pdgfrb-cre/ERT2)6096Rha*	*Xiang Yu, Peking University*

Ai47	*Gt(ROSA)26Sortm47(CAG-EGFP*)Hze*	*Zilong Qiu, Chinese Academy of Sciences Center for Excellence in Brain Science and Intelligence Technology, Chinese Academy of Sciences.*
Ai14	*B6.Cg-Gt(ROSA)26Sortm14(CAG-tdTomato)Hze/J*	*Woo-Ping Ge, Chinese Institute of Brain Research (CIBR)*
Grin2d	*B6/JGpt-Grin2dem1Cflox/Gpt*	*GemPharmatech*
Grin1	*Grin1tm2Stl*	*Yangang Sun, Chinese Academy of Sciences Center for Excellence in Brain Science and Intelligence Technology, Chinese Academy of Sciences*

Ptgs2	*C57BL/6JCya-Ptgs2em1flox/Cya*	*Cyagen*
Acta2	*C57BL/6JGpt-Acta2em1Cflox/Gpt*	*GemPharmatech*
Glrb	*C57BL/6JGpt-Glrbem1Cflox/Gpt*	*GemPharmatech*
