# Supplementary material for: Neurovascular coupling in the basolateral amygdala modulates negative emotions
Source: Cell Res. 2026 May 27;36(9):656–79. doi: 10.1038/s41422-026-01256-2 (PMC13503848; doi:10.1038/s41422-026-01256-2)
Supplement: Supplementary file 22 [file 41422_2026_1256_MOESM31_ESM.pdf]

Supplementary Table S4. Antibodies information

Name	Host	Cat#	Dilution	Company
Anti-NMDAR2D Antibody	Mouse	MAB5578	1:400 (IF) 1:2,500 (WB) 1:1,000 (IP)	Merck
Anti-Smoothelin antibody	Rabbit	ab219652	1:400 (IF)	abcam
α-Smooth Muscle Actin (ACTA2) Rabbit mAb	Rabbit	A17910	1:2,500 (WB)	ABclonal
ACE2 Recombinant Rabbit Monoclonal Antibody (SN0754)	Rabbit	MA5-32307	1:400 (IF)	Invitrogen
Gephyrin Polyclonal Antibody	Rabbit	PA5-29036	1:400 (IF)	Invitrogen
BD Pharmingen™ Purified Rat Anti-Mouse CD31	Rat	557355	1:200 (IF)	BD
Anti-NeuN purified Antibody	Guinea Pig	ABN90P	1:800 (IF)	Merck
COX2/PTGS2 Rabbit pAb	Rabbit	A1253	1:400 (IF)	ABclonal
c-Fos antibody	Rabbit	226 003	1:1,000 (IF)	SYSY

Recombinant Alexa Fluor® 488 Anti-Caldesmon/CDM antibody	Rabbit	ab208116	1:400 (IF) 1:2,500 (WB)	abcam
iFluor™ 488 Conjugated Goat anti-mouse IgG polyclonal Antibody	Goat	HA1125	1:400 (IF)	HUABIO
Mouse Aminopeptidase N/CD13 Antibody	Goat	AF2335	1:200 (IF)	R&D
CD140b (PDGFRB) Monoclonal Antibody (APB5), APC, eBioscience™	Rat	17-1402-82	1:50 (FCM)	Invitrogen
Mice IgG	Mouse	A7028	1:1,000 (IP)	Beyotime
Donkey anti-Mouse IgG (H+L) ReadyProbes™ Secondary Antibody, Alexa Fluor™ 488	Donkey	R37114	1:1,000 (IF)	Invitrogen
Donkey anti-Rabbit IgG (H+L) ReadyProbes™ Secondary Antibody, Alexa Fluor™ 488	Donkey	R37118	1:1,000 (IF)	Invitrogen

Donkey anti-Rabbit IgG (H+L) Highly Cross-Adsorbed Secondary Antibody, Alexa Fluor™ 546	Donkey	A10040	1:1,000 (IF)	Invitrogen
Donkey anti-Rat IgG (H+L) Highly Cross-Adsorbed Secondary Antibody, Alexa Fluor™ 488	Donkey	A21208	1:1,000 (IF)	Invitrogen
Donkey anti-Rat IgG (H+L) Highly Cross-Adsorbed Secondary Antibody, Alexa Fluor™ 555	Donkey	A78945	1:1,000 (IF)	Invitrogen
Donkey anti-Rat IgG (H+L) Highly Cross-Adsorbed Secondary Antibody, Alexa Fluor™ Plus 647	Donkey	A48272	1:1,000 (IF)	Invitrogen
Alexa Fluor® 647 AffiniPure™ Donkey Anti-Guinea Pig IgG (H+L)	Donkey	706-605-148	1:1,000 (IF)	Jackson ImmunoResearch
β-Tubulin Mouse mAb	Mouse	AC021	1:5,000 (WB)	ABclonal
Goat Anti-Mouse IgG, HRP Conjugated	Goat	CW0102	1:10,000 (WB)	CWBIO

Goat Anti-Rabbit IgG, HRP Conjugated	Goat	CW0103	1:10,000 (WB)	CWBIO
DDDDK-Tag Rabbit mAb	Rabbit	AE063	1:400 (IF)	ABclonal
Hif-1α	Rabbit	ER1802-41	1:100 (IF)	HUABIO
