# Supplementary material for: Neurovascular coupling in the basolateral amygdala modulates negative emotions
Source: Cell Res. 2026 May 27;36(9):656–79. doi: 10.1038/s41422-026-01256-2 (PMC13503848; doi:10.1038/s41422-026-01256-2)
Supplement: Supplementary file 23 [file 41422_2026_1256_MOESM32_ESM.pdf]

Supplementary Table S5. Primer pairs information

Name		Sequence
Grin1	F	AAGAACCTGCAGCAGTACCA
	R	GCAGCAGGACTCATCAGTGT
Grin2a	F	TCAGGGACTTTGGAAAACGCT
	R	AAGTCATGCAGGGTTGGTTG
Grin2b	F	ACTAACTATCAATGAAGAACGGT
	R	CCAGACCCCAGAGTAACCA
Grin2c	F	TGTGGGCCTTCTTCGCTGTCATCT
	R	TGCCATTAGGTACCGTGCCAAAAC
Grin2d	F	TGGAGGAGTACGACTGGACAT
	R	CGCACTGACACTACGGAGC
Grin3a	F	CTGCACACCAGTCAGAGGTT
	R	TTCGTTGGTTGTCGTGACTC
Grin3b	F	TGGGGGTCTACCACTTGTCA
	R	TGGATCTTCTGGCTCGTGTG
	F	TTCAAAGGCATTCCTGTTGATGT

Glrb	R	TGAGCCTCTGAAGTCACTAGG
Cald1	F	GCCGTTCAAGTGCTTCACTC
	R	TGTCAATCTTGGAGACTACTGCT
18s	F	CGGACACGGACAGGATTGACA
	R	CCAGACAAATCGCTCCACCAACTA
Cald1 *(siRNA)*	F	GUUUGAUCCGACCAUAACAGA
	R	UGUUAUGGUCGGAUCAAACUC
