# Supplementary material for: Neurovascular coupling in the basolateral amygdala modulates negative emotions
Source: Cell Res. 2026 May 27;36(9):656–79. doi: 10.1038/s41422-026-01256-2 (PMC13503848; doi:10.1038/s41422-026-01256-2)
Supplement: Supplementary file 24 [file 41422_2026_1256_MOESM3_ESM.pdf]

Figure S1

Ruan et al.

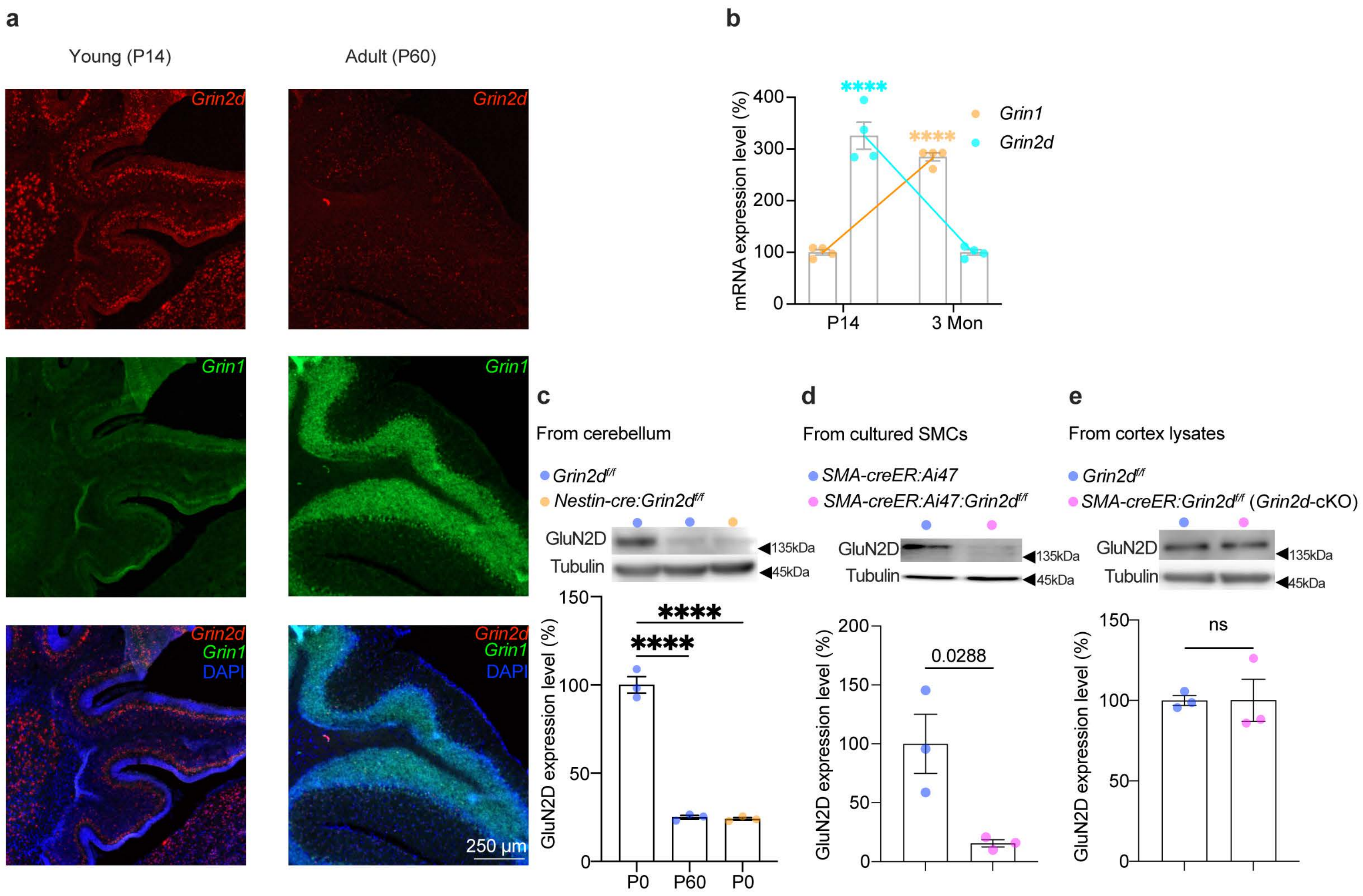

Fig.S1 Validation of successful GluN2D conditional Knock-out. a, b Representative images (**a**) and quantification (**b**) of *Grin1* and *Grin2d* mRNA expression via RNAscope in the cerebellum. Each dot represents one region of interest (ROI) from a single brain slice per mouse. Student's *t*-test. **c-e** Representative immunoblot (top) and quantification (bottom) of GluN2D protein levels in P0/P60 cerebellum lysates (**c**), cultured SMCs sorted from P0 parenchymal tissue (**d**), and P60 cortex lysates(**e**). Each dot represents one mouse. One-way ANOVA followed by Tukey's multiple comparisons test for (**c,** $P < 0.0001$), and Student's *t*-test for (**d**, **e**). **** indicates $P < 0.0001$. Data are presented as mean ± SEM.
