# Supplementary material for: Neurovascular coupling in the basolateral amygdala modulates negative emotions
Source: Cell Res. 2026 May 27;36(9):656–79. doi: 10.1038/s41422-026-01256-2 (PMC13503848; doi:10.1038/s41422-026-01256-2)
Supplement: Supplementary file 25 [file 41422_2026_1256_MOESM4_ESM.pdf]

Figure S2

Ruan et al.

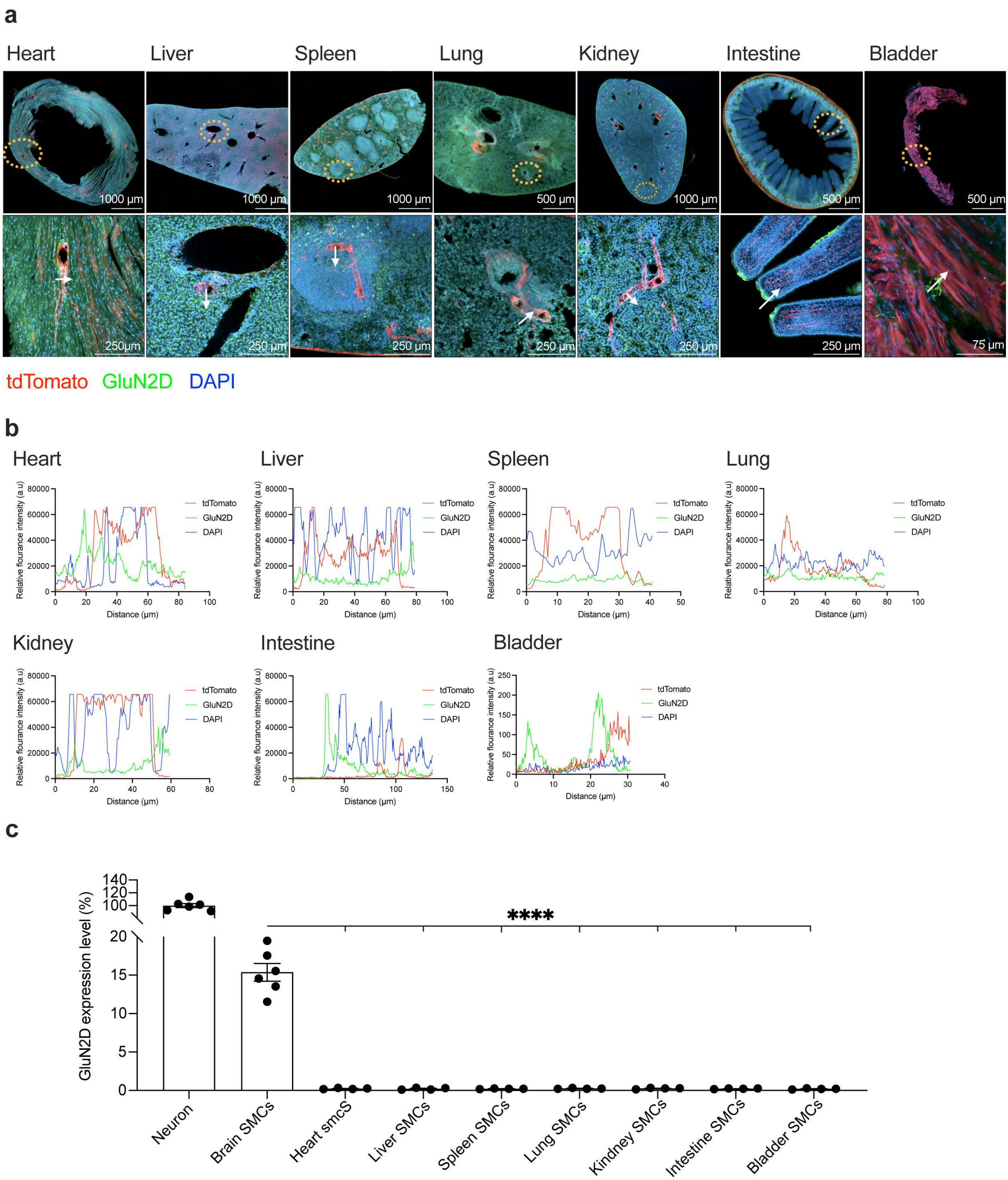

Fig.S2 Validation of GluN2D expression in peripheral organs' SMCs. a Representative low-magnification images of tissue sections from the heart, liver, spleen, lung, kidney, small intestine, and bladder of *SMA-creER:Ai14* mice (top). Yellow dotted boxes indicate the magnified areas shown below. White arrows indicate ROIs for fluorescence intensity line-scan analysis. **b** Fluorescence intensity distribution (line-scan) across the ROIs indicated by white arrows in (**a**). **c** Quantification of GluN2D immunofluorescence (IF) staining intensity of GluN2D in SMCs across tissues. Each dot represents one mouse. One-way ANOVA followed by Tukey's multiple comparisons test (P < 0.0001). **** indicates P < 0.0001. Data are presented as mean ± SEM.
