# Supplementary material for: Neurovascular coupling in the basolateral amygdala modulates negative emotions
Source: Cell Res. 2026 May 27;36(9):656–79. doi: 10.1038/s41422-026-01256-2 (PMC13503848; doi:10.1038/s41422-026-01256-2)
Supplement: Supplementary file 26 [file 41422_2026_1256_MOESM5_ESM.pdf]

Figure S3

Ruan et al.

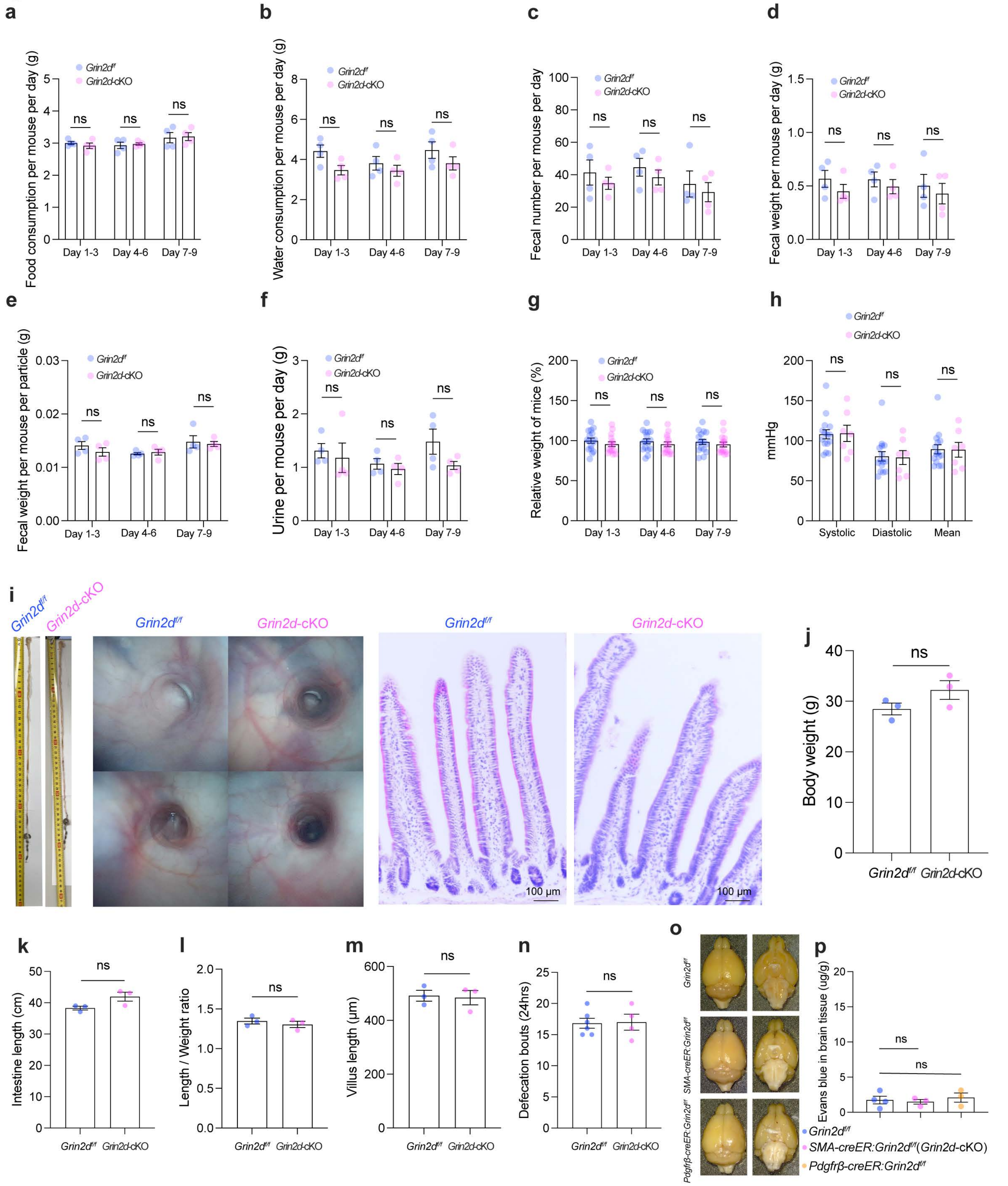

Fig.S3 Verification of mouse metabolic status, gut health, and blood-brain barrier integrity. a-h Quantification of food consumption (**a**), water intake (**b**), number of fecal pellets (**c**), daily fecal weight (**d**), weight per pellet (**e**), urine output (**f**), relative body weight changes (**g**) in the home cage during three consecutive 3-day detection cycles (specifically Days 1-3, 4-6, and 7-9), and blood pressure measurement (**h**). For (**a-f**), each dot represents the average value per cage (4 mice per cage). For (**g, h**), each dot represents an individual mouse. Mann-Whitney test (**c**) and Student's *t*-test (except **c**). **i** Representative images of intestinal morphology (appearance and length) and H&E staining of intestinal sections. **j-n** Quantification of data from (**i**), including mice body weight (**j**), intestinal length (**k**), intestinal length-to-body weight ratio (**l**), villus length (**m**), and total 24-hour defecation count (**n**) for mice in the home cage. Each dot represents one mouse. Student's *t*-test. **o, p** Representative images of the brain following retro-orbital Evans Blue injection (**o**) and the corresponding quantification (**p**). Each dot represents one mouse. One-way ANOVA followed by Tukey's multiple comparisons test (P = 0.7654). Data are presented as mean ± SEM.
