# Supplementary material for: Neurovascular coupling in the basolateral amygdala modulates negative emotions
Source: Cell Res. 2026 May 27;36(9):656–79. doi: 10.1038/s41422-026-01256-2 (PMC13503848; doi:10.1038/s41422-026-01256-2)
Supplement: Supplementary file 27 [file 41422_2026_1256_MOESM6_ESM.pdf]

Figure S4 **Ruan et al.**

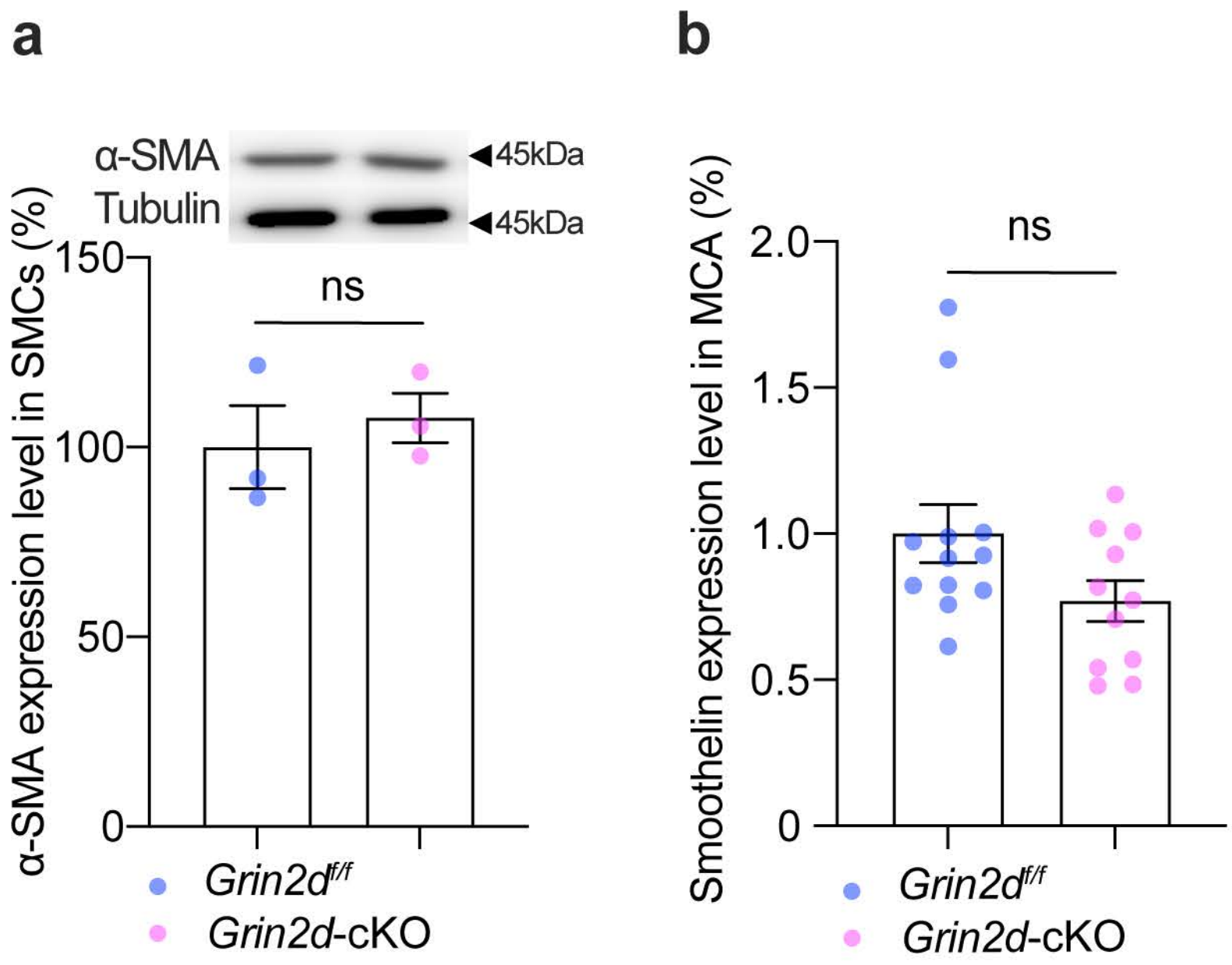

Fig.S4 Validation of SMC marker proteins in *Grin2d*-cKO mice. a Representative immunoblot (top) and quantification (bottom) of α-SMA expression in the cortex lysates. Each dot represents one mouse. Student's *t*-test. **b** Quantification of Smoothelin immunofluorescence (IF) staining in the middle cerebral artery (MCA), corresponding to the representative images in **Fig. 2d**. Each dot represents one analysis region of MCA from a single mouse. *n* = 4 mice per group. Student's *t*-test. Data are presented as mean ± SEM.
