# Supplementary material for: Neurovascular coupling in the basolateral amygdala modulates negative emotions
Source: Cell Res. 2026 May 27;36(9):656–79. doi: 10.1038/s41422-026-01256-2 (PMC13503848; doi:10.1038/s41422-026-01256-2)
Supplement: Supplementary file 28 [file 41422_2026_1256_MOESM7_ESM.pdf]

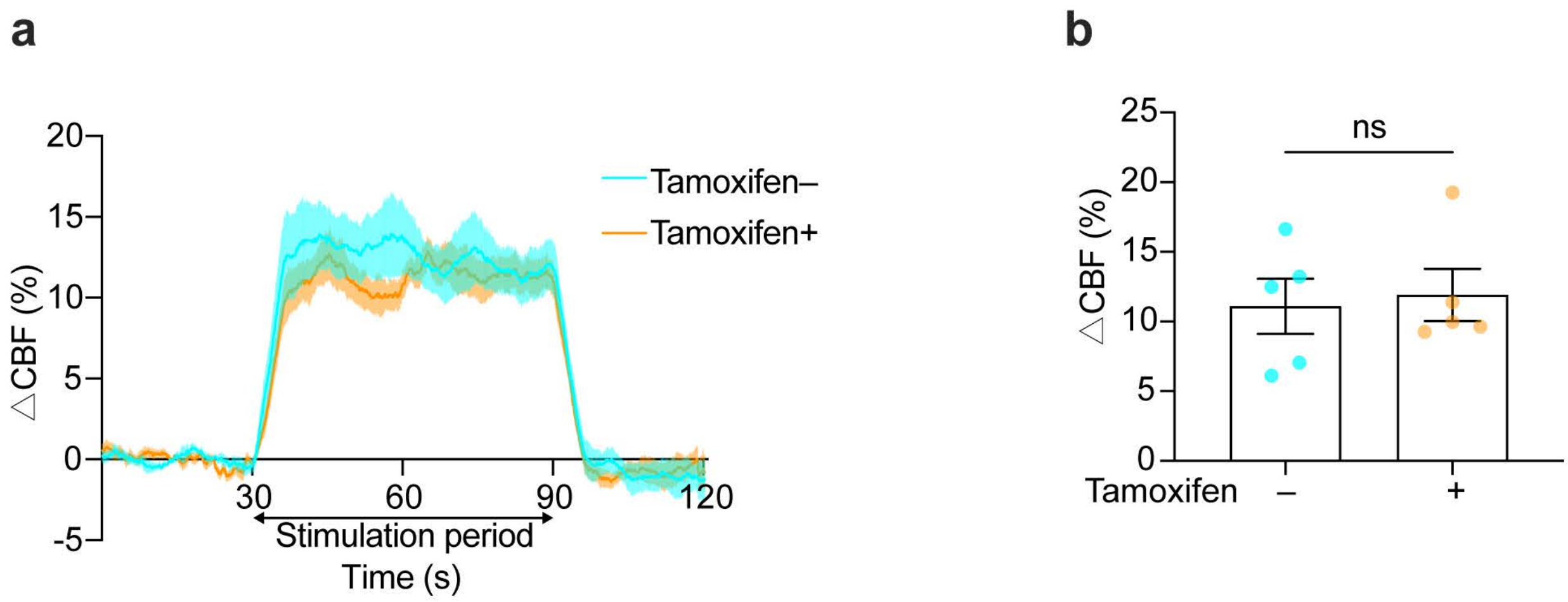
a
20
15
10
5
0
-5
△CBF (%)
Tamoxifen–
Tamoxifen+
30
60
90
120
Stimulation period
Time (s)
b
25
20
15
10
5
0
△CBF (%)
ns
Tamoxifen
–
+

Fig.S5 The effect of tamoxifen on NVC. a Whisker-evoked functional hyperemia in the S1BF of littermate control mice treated with tamoxifen. Shaded areas represent SEM across mice. **b** Quantification of the functional hyperemia plateau phase from the data in (**a**). Each dot represents one mouse. Student's *t*-test. Data are presented as mean ± SEM.
