# Supplementary material for: Neurovascular coupling in the basolateral amygdala modulates negative emotions
Source: Cell Res. 2026 May 27;36(9):656–79. doi: 10.1038/s41422-026-01256-2 (PMC13503848; doi:10.1038/s41422-026-01256-2)
Supplement: Supplementary file 29 [file 41422_2026_1256_MOESM8_ESM.pdf]

a

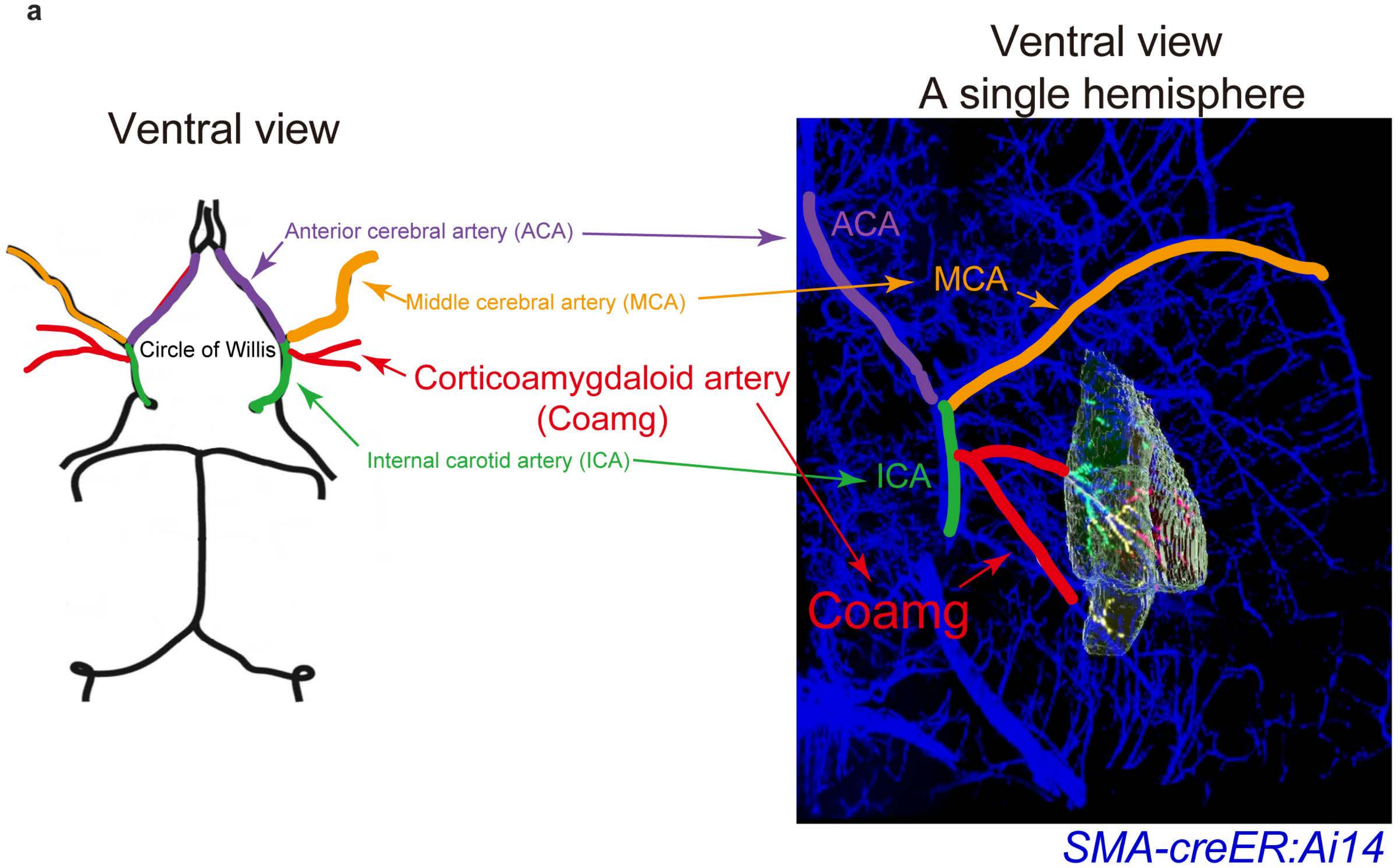
Ventral view
Anterior cerebral artery (ACA)
Middle cerebral artery (MCA)
Circle of Willis
Corticoamygdaloid artery
(Coamg)
Internal carotid artery (ICA)
Ventral view
A single hemisphere
ACA
MCA
ICA
Coamg
SMA-creER:Ai14

b

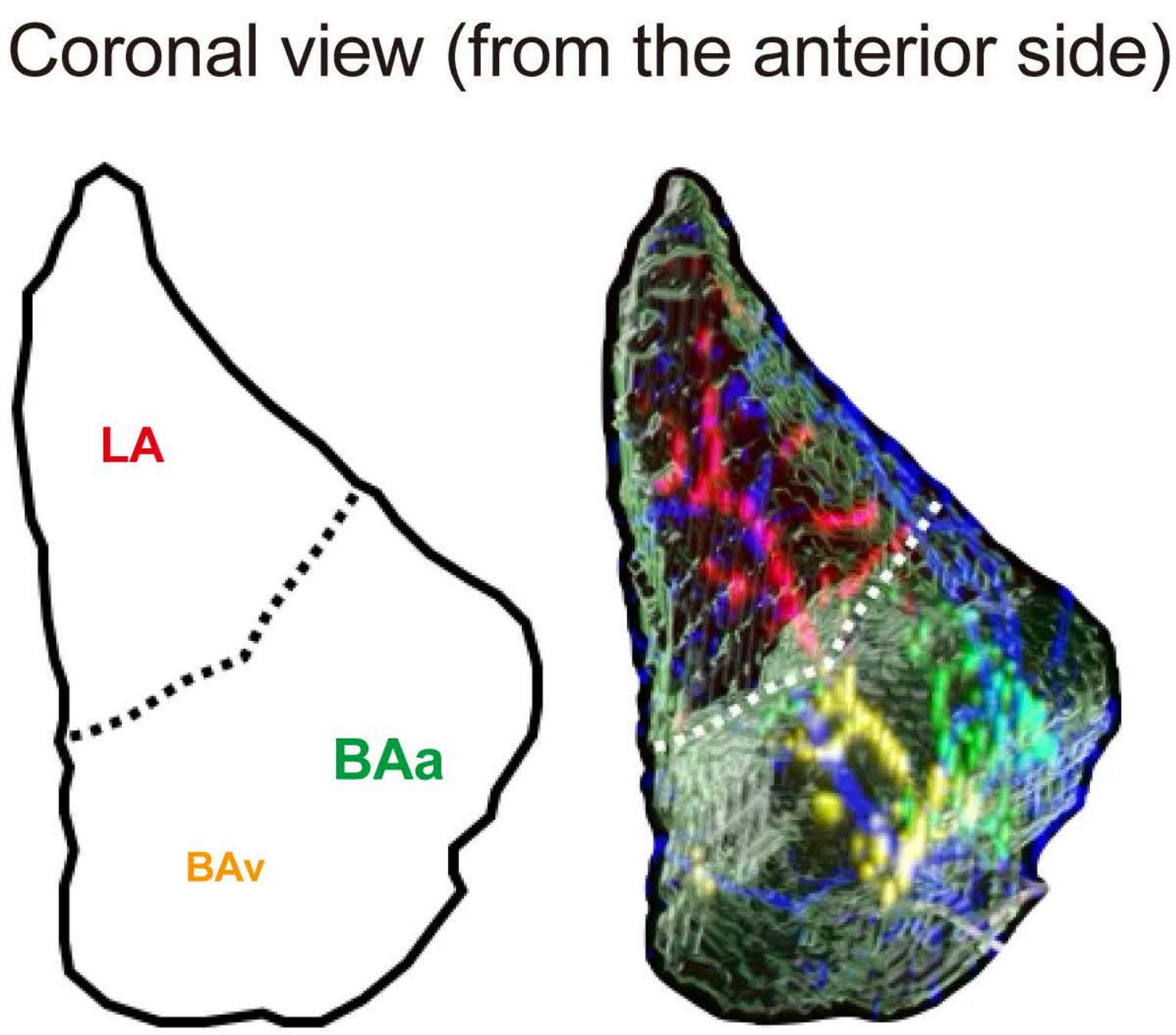
Coronal view (from the anterior side)
LA
BAa
BAv

Fig.S6 3D-Reconstruction of BLA arteriolar topology through correlative integration of the Allen Brain Atlas with tissue-cleared brains from *SMA-creER:Ai14* mouse. a Ventral view of the mouse BLA. Schematic diagram (left) was adapted from *The Mouse Nervous System* (p. 460, Fig. 14.1), and 3D reconstruction (right) was generated in Imaris using the Allen Mouse Brain Common Coordinate Framework plugin. BLA is denoted translucent grey. Blue denotes arteriolar SMCs. Colored lines represent distinct arteriolar segments. **b** Coronal view of the mouse BLA along with arterioles inside. Colored lines indicate arterioles in the basal amygdala anterior part (BAa), basal amygdala ventral part (BAv), and lateral amygdala (LA).
