# Supplementary material for: Neurovascular coupling in the basolateral amygdala modulates negative emotions
Source: Cell Res. 2026 May 27;36(9):656–79. doi: 10.1038/s41422-026-01256-2 (PMC13503848; doi:10.1038/s41422-026-01256-2)
Supplement: Supplementary file 30 [file 41422_2026_1256_MOESM9_ESM.pdf]

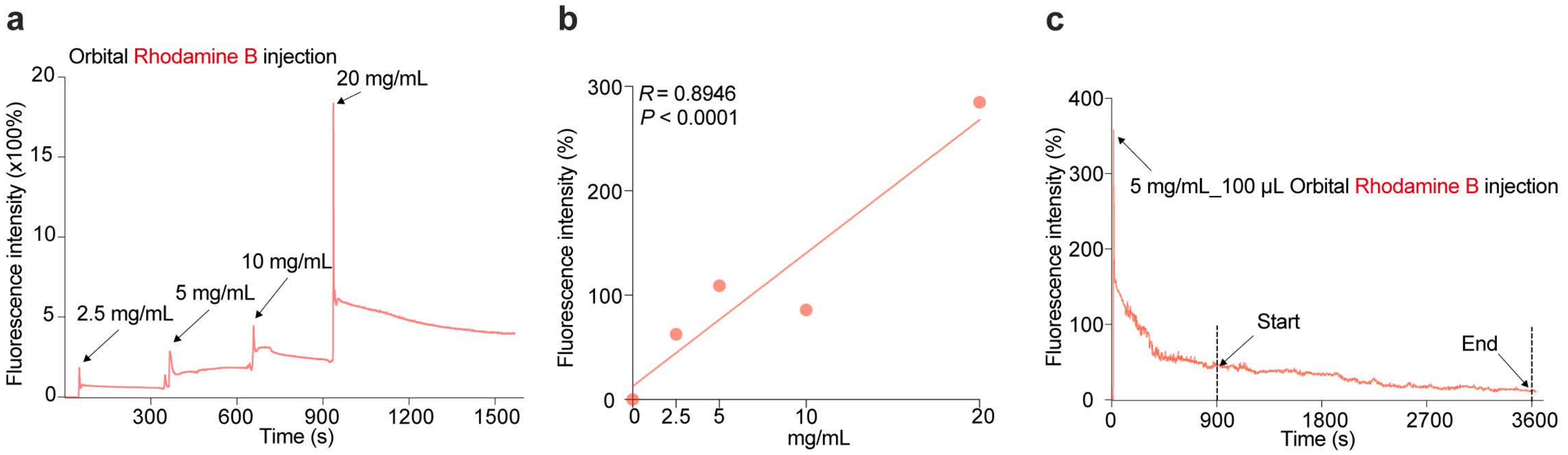
a
Orbital Rhodamine B injection
Fluorescence intensity (x100%)
20 mg/mL
10 mg/mL
5 mg/mL
2.5 mg/mL
Time (s)
b
R= 0.8946
P < 0.0001
Fluorescence intensity (%)
mg/mL
c
Fluorescence intensity (%)
5 mg/mL_100 μL Orbital Rhodamine B injection
Start
End
Time (s)

Fig.S7 Calibration and validation of plasma Rhodamine B fluorescence intensity. **a** Real-time fluorescence intensity measurement following retro-orbital injection of various Rhodamine B concentrations. **b** Linear correlation between Rhodamine B concentration and fluorescence intensity in (**a**). Each dot represents the average intensity from 120-150 s post-injection for a specific concentration. **c** Real-time fluorescence intensity measurements following retro-orbital injection of 5 mg/mL Rhodamine B, showing signal stability from 900-3600 s post-injection.
